# SmartTrap: automated precision experiments with optical tweezers

**DOI:** 10.1038/s41592-026-03129-3

**Published:** 2026-06-18

**Authors:** Martin Selin, Antonio Ciarlo, Giuseppe Pesce, Lars Bengtsson, Joan Camunas-Soler, Vinoth Sundar Rajan, Fredrik Westerlund, L. Marcus Wilhelmsson, Isabel Pastor, Felix Ritort, Steven B. Smith, Carlos Bustamante, Giovanni Volpe

**Affiliations:** 1 Physics Department, University of Gothenburg, Gothenburg, Sweden; 2 Department of Neuroscience, Reproductive Sciences and Dentistry, Università degli Studi di Napoli ‘Federico II’, Naples, Italy; 3 Department of Physics, Università degli Studi di Napoli ‘Federico II’ Complesso Universitario Monte S. Angelo, Naples, Italy; 4 Department of Medical Biochemistry and Cell Biology, Institute of Biomedicine, University of Gothenburg, Gothenburg, Sweden; 5 Wallenberg Centre for Molecular and Translational Medicine, Sahlgrenska Academy, University of Gothenburg, Gothenburg, Sweden; 6 Science for Life Laboratory, Institute of Biomedicine, University of Gothenburg, Gothenburg, Sweden; 7 Department of Life Sciences, Chalmers University of Technology, Gothenburg, Sweden; 8 Department of Chemistry and Chemical Engineering, Chalmers University of Technology, Gothenburg, Sweden; 9 Small Biosystems Lab, Condensed Matter Physics Department, University of Barcelona, Barcelona, Spain; 10 Institut de Nanociència i Nanotecnologia (IN2UB), Universitat de Barcelona, Barcelona, Spain; 11 Reial Acadèmia de Ciències i Arts de Barcelona (RACAB), Barcelona, Spain; 12 Steven B. Smith Engineering, Los Lunas, NM USA; 13 Department of Molecular and Cell Biology, University of California, Berkeley, Berkeley, CA USA; 14 California Institute for Quantitative Biosciences, University of California, Berkeley, Berkeley, CA USA; 15 Jason L. Choy Laboratory of Single-Molecule Biophysics, University of California, Berkeley, Berkeley, CA USA; 16 Department of Physics, University of California, Berkeley, Berkeley, CA USA; 17 Howard Hughes Medical Institute, University of California, Berkeley, Berkeley, CA USA; 18 Kavli Energy Nanoscience Institute, University of California, Berkeley, Berkeley, CA USA; 19 SciLifeLab, Physics Department, University of Gothenburg, Gothenburg, Sweden

**Keywords:** Single-molecule biophysics, Microscopy, DNA, Nanoscale biophysics, Optical tweezers

## Abstract

Optical tweezers are widely used in single-molecule biophysics, cell biomechanics and soft matter physics, but require a human operator, limiting throughput and repeatability. Here we present a smart optical tweezers platform, named SmartTrap, capable of performing complex experiments autonomously by integrating real-time three-dimensional particle tracking, custom electronics and a microfluidics system. Through a series of experiments, we demonstrate it can operate continuously, acquiring high-precision data over extended periods of time. By bridging the gap between manual experimentation and autonomous operation, SmartTrap establishes a robust and open-source framework for the next generation of optical tweezers research, capable of performing large-scale studies in single-molecule biophysics, cell mechanics and colloidal science with minimal experimental overhead and operator bias.

## Abstract (web summary)

SmartTrap is a smart optical tweezers platform that integrates real-time three-dimensional particle tracking, electronics and microfluidics to perform single-molecule biophysics and cell mechanics experiments with minimal human intervention.

## Main

Artificial intelligence (AI) has advanced at a staggering pace and has resulted in novel tools from conversational chatbots to foundation models such as AlphaFold, which has revolutionized automated protein structure prediction and design^1–3^. Although such breakthroughs have benefited many computational domains, the integration of AI into experimental sciences still faces major hurdles, not least due to variability between experimental protocols^4^.

Optical tweezers started with Ashkin’s demonstration of particle trapping by radiation pressure in 1970 (ref. ^5^) and of a single-beam optical trap in 1986 (ref. ^6^). They have found use in a broad range of fields, from physics to biology and chemistry, to exert and measure microscopic forces^7^. For example, in physics, they have been used for trapping and manipulating microscopic particles, enabling precise studies of soft matter and nonequilibrium dynamics^8,9^. In biophysics, they have enabled precision measurements of the forces involved in the stretching of single molecules^10^, of the forces generated by biomolecular motors^11^ and of the mechanical properties of membranes^12^. In colloidal chemistry, optical tweezers have proven invaluable for probing interparticle interactions^13^, critical Casimir forces^14^ and depletion interactions^15^.

Optical tweezers excel at manipulating single objects with high specificity, accuracy and resolution. This makes them the most precise force spectroscopy technique available and is what enables the direct characterization of energy processes and thermodynamics at the single-molecule level, with measurement accuracies reaching about 0.1 kcal mol^−^^1^—exceeding those of alternative high-throughput approaches^16^. However, this inherent focus on single-object manipulation limits throughput and represents one of the main drawbacks of the technique. Experiments are conducted on one particle, molecule or cell at a time, typically requiring continuous supervision by a trained practitioner. Furthermore, this dependence on manual operation greatly increases the time and cost of collecting large datasets, while also introducing the potential for human bias, reducing reproducibility.

The field is starting to move away from manual operation in large part thanks to the rise of deep learning^17^, which has recently been used to enhance optical tweezers^18^. Various aspects of optical tweezers’ experimental procedures have been automated. For example, automated optical tweezers have been used to position particles with high precision to construct crystal-like structures^19^, and a combination of real-time image analysis and machine learning has been used to automatically trap and classify particles^20^. However, to date, precision measurements, such as experiments with single molecules, single cells and individual colloidal interactions, have been out of reach for fully autonomous procedures because of their complexity. Still, it would be valuable to increase throughput, especially for complex experiments such as single-molecule experiments, where data are challenging to gather^10^. Automation would also be ideal for studying rare or transient events, such as misfolding of proteins, and for sampling of heterogeneous distributions.

Here, we present SmartTrap, a smart and open-source optical tweezers platform designed to perform advanced experiments without human intervention. This is achieved through completely digital control over experimental procedures and smart event-driven algorithms. Digital control is made possible by integrating custom-built electronics, microfluidics and optical controls into a single software system. Combining this with real-time deep-learning-based image analysis and closed-loop feedback algorithms enables SmartTrap to react to events and perform complex experimental procedures autonomously. We demonstrate the versatility of SmartTrap across four experiments, which are well known and widely studied in the literature but have never been fully automated: particle size characterization, single-molecule DNA stretching to observe force-induced overstretching transitions, optical deformation of red blood cells to probe membrane stiffness and measurement of electrostatic forces between colloidal particles to characterize short-range interactions. By making both software and hardware open source, we aim to inspire others to use and build on SmartTrap, thus establishing a framework for smart optical tweezers, with a variety of applications in biophysics, cell mechanics and colloidal science.

## Results

### The optical tweezers system

The SmartTrap optical tweezers system utilizes two counterpropagating lasers to create an optical trap, with forces measured directly from the momentum change of the trapping beams^21^. We developed a custom electronics controller for this system, which controls the positioning of the lasers and sample stage while simultaneously measuring the position and deflection of the lasers. SmartTrap also integrates microfluidic pumps, laser power controls and a digital camera into a single control software. A more detailed description of the instrument can be found in the ‘Optical tweezers system’ section of Methods, while the key features are outlined below.

**Fig. 1:**
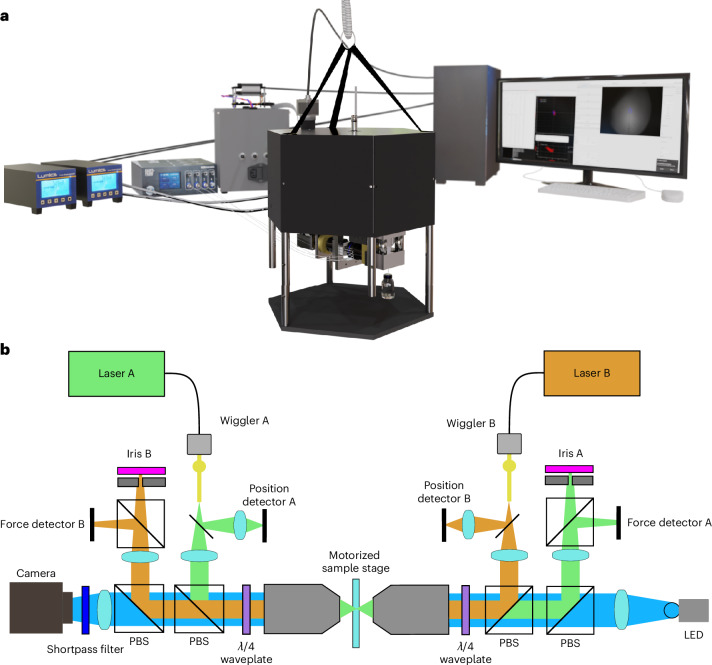
SmartTrap setup. **a**, 3D illustration of the SmartTrap optical tweezers instrument and control system. The instrument is in the front and the control system behind it with the computer and interface on the right and the controllers on the left. **b**, Schematics of the optics. Lasers A and B follow mirrored pathways and form a counterpropagating optical trap in the sample. Along each laser path, there are two two-dimensional PSDs: the first monitors the laser position and the second measures the force from the laser on trapped objects from the scattered light. PBS, polarizing beam splitter; LED, light emitting diode.

The SmartTrap setup is illustrated in Fig. 1. It uses a counterpropagating arrangement, which is best understood by following the path of laser A. The laser exits the optical fiber through a wiggler. This is a custom actuator designed for two-dimensional positioning of the laser by tilting (wiggling) the optical fiber using piezoelectric actuators, thus moving the lasers in the plane of the sample; see the ‘Laser wigglers’ section in Methods. A position-sensitive detector (PSD) placed before the sample (‘Position detector A’) detects the position of the laser, providing a signal that can be exploited in a feedback loop. After the sample, a second PSD (‘Force detector A’) measures the deflection of the laser caused by the optically trapped object allowing the measurement of the lateral momentum transfer and, therefore, the lateral optical force. A photodiode with an iris (‘Iris A’) measures the size of the laser spot, which is used to determine the force acting on the particle along the axial direction. Laser B follows a path that mirrors that of laser A. Lasers A and B together form a counterpropagating optical tweezers that stably traps particles while directly measuring the trapping force^21^. In this configuration, the laser beam diameters are smaller than the back apertures of the objective lenses. This ensures that all the light passes through the lenses and enables the light-momentum approach to force measurement. Conversely, the gradient force is weaker than when the laser beam is larger than the back aperture, a condition that does not permit trapping with a single beam.

**Extended Data Fig. 1:**
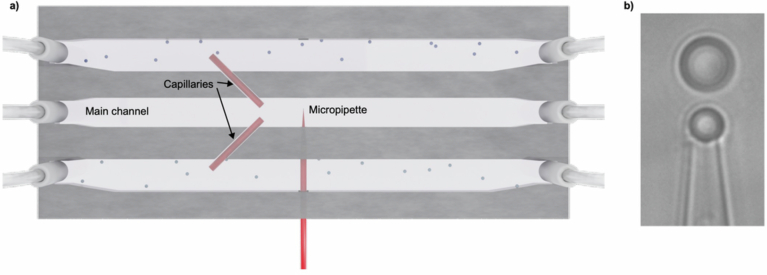
Microfluidic chamber. (**a**) Illustration of the microfluidic chamber. There are three separate channels. The middle channel is used to perform the experiment and contains the tip of the micropipette where one of the particles is held. The bottom and top channels are used to flow particles into the main channel via glass capillaries. The micropipette and the capillaries are colored in red for clarity. The channels are connected to a microfluidic pump with independent operation. (**b**) Picture obtained from an experiment while it is being set up: there is one particle in the optical trap (top) and one in the pipette (bottom).

The system uses a custom microfluidic chamber with three parallel channels (Extended Data Fig. 1a). The experiments are performed in the central channel; there are also two side channels that carry the different particles and are connected to the central channel through capillaries. By increasing the flow in a side channel, particles are dispensed into the central one. The microfluidic chamber also contains a micropipette, connected to a digitally controlled pump, with the tip of the pipette positioned in the middle of the central channel. During experiments with two particles, one of the particles is held by the micropipette using suction (Extended Data Fig. 1b).

The custom electronic controller steers the sample stage and the positions of the lasers, while also reading the various photosensors. This enables us to implement simple feedback algorithms directly on the controller, for example, to move the trap at constant speed until a certain force value is reached. The latency of algorithms run on the controller is very low (circa 0.1 ms), resulting in faster feedback than when sending commands from the computer (circa 10 ms). This controller communicates with the host computer using a serial USB protocol and connects with a standard USB-C cable. The laser power supplies and the microfluidic pump are controlled by separate commercial controllers.

All the controllers, the photodiodes and the camera are connected to a host computer running a custom Python program providing a graphical user interface (GUI). This provides the user with full digital monitoring and control of the SmartTrap and enables synchronized control of its different components. Further details on the software and electronics are outlined in ‘Optical tweezers system’ in Methods.

### Real-time image analysis

To automate the experimental procedures, it is essential to analyze the video feed from the camera. We implemented this analysis on the host computer using deep learning because of its precision, versatility and speed^22^.

**Extended Data Fig. 2:**
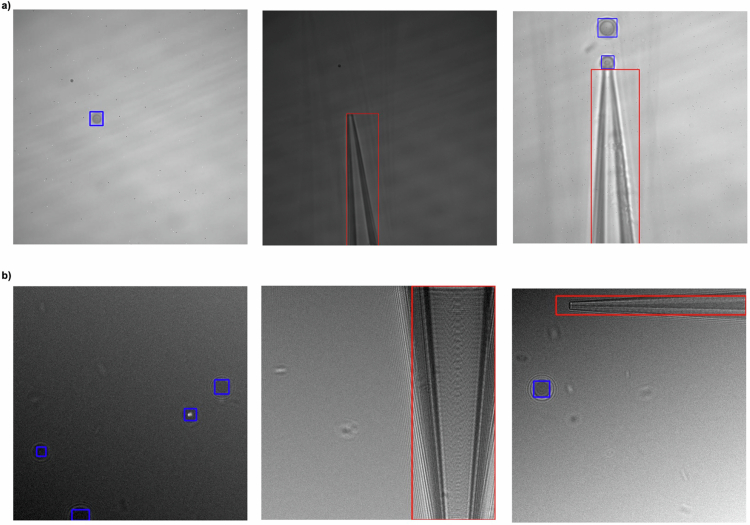
YOLO training data. Example of training data used for the YOLO network. (**a**) Manually-annoted data from experiments and (**b**) simulated training data obtained using the DeepTrack2 Python package. The blue boxes highlight the particles and the red boxes highlight the pipette.

The first step of the image analysis consists of detecting the presence of the particles and the pipette. This is done using an artificial neural network utilizing the You Only Look Once (YOLO) object detection framework^23^ and, in particular, YOLO V5 (ref. ^24^). We chose YOLO because it is the best approach we have tried for detecting both the micropipette and the particles. We trained the network using a dataset consisting primarily of synthetic images simulated using the DeepTrack2 software package^22,25^, for which the ground truth is known exactly. Furthermore, we also included 1,000 manually annotated experimental images because this improved the detection accuracy, which we attribute to challenges in accurately simulating a micropipette. Example images from the training dataset, including the targets, are shown in Extended Data Fig. 2. The network predictions consist of bounding boxes around the objects of interest. The sizes of the boxes give an estimate of the particle sizes and the extent of the micropipette. The centers of the boxes provide the positions of the particles in the *xy-*directions. Also, thanks to the versatility of this approach, the SmartTrap can easily handle nonspherical particles or objects (as illustrated by the pipette detection), requiring only a quick retraining of the YOLO network.

**Extended Data Fig. 3:**
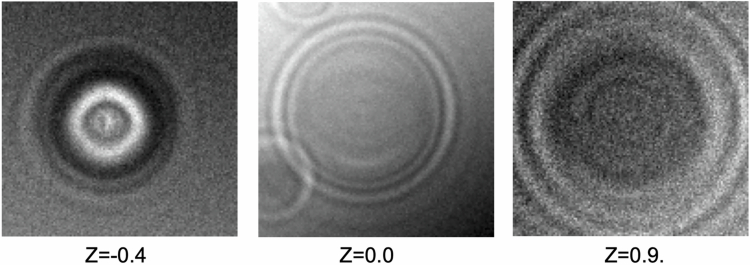
Simulated particles with different focal positions. Examples of simulated images used for training the convolutional neural network to predict the focal position of particles. The images are obtained using the DeepTrack2 Python package. The numbers below the pictures indicate the target z-value.

**Extended Data Fig. 4:**
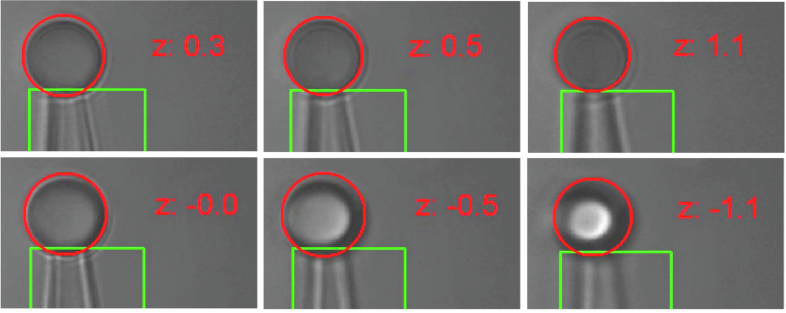
Experimental predictions of the particle and pipette positions. Screenshots from the program predicting the lateral positions of the particle (red circle) and of the pipette (green box). It also predicts the axial position (z-position) of the particle relative to the focus, written in red.

For many experiments, the optically trapped particle and the particle held in the pipette need to be in the same plane along the *z*-direction (axial direction). This means that the relative axial position of the two particles needs to be accurately estimated. We did this using a convolutional neural network trained with images of the particles simulated with the DeepTrack2 package^22,25^ (Extended Data Fig. 3). The convolutional network performs its predictions taking as input a 128 × 128-pixel image of the particle centered on the bounding box determined using the YOLO algorithm. The network predicts the position of the particles in arbitrary units allowing the alignment feedback algorithm to match the predictions of the particle in the trap to the particle held by the pipette by moving the sample along the *z*-direction. Example predictions for particles in the size range used for the experiments (from 2 μm to 4 μm in diameter) are shown in Extended Data Fig. 4 and the performance of the networks is shown in Supplementary Video 1.

The *z*-prediction also serves a second purpose in determining whether a second particle has entered the trap. If a second particle enters the trap, this will displace the trapped particle along the *z* axis in the trap leading to a shift in the *z*-predictions, which is used to determine if the system has accidentally trapped more than one particle.

Finally, we highlight that users can easily implement their own real-time image analysis method in the SmartTrap software with an easy-to-use user interface while maintaining fully automated processes. Further details on the neural networks used and how they are trained are described in ‘Artificial neural networks’ in Methods.

### Feedback algorithms

**Fig. 2:**
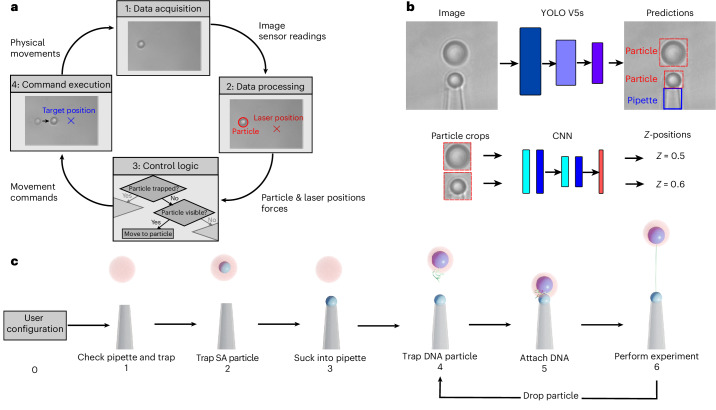
Algorithms used for automation. **a**, Data processing feedback loop. After acquisition (step 1), data are processed (step 2) to extract information about the experiment. This information is then used to make a decision about what to do next (step 3), which is then executed by the instrument (step 4). **b**, Neural networks used in the image analysis. First, the objects of interest are located with YOLO. Then, the *z*-positions of the particles are determined with the help of a convolutional neural network (CNN). **c**, Main steps of an autonomous DNA pulling experiment executed with SmartTrap. It starts with a configuration procedure (step 0), followed by the system investigating whether there is a particle in the pipette and the optical trap (step 1). Step 2 is getting the streptavidin (SA) particle followed by step 3 when the particle is held by suction into the pipette. In step 4, the particle with attached DNA is collected. In step 5, the system tries to attach the DNA by gently pushing the particles together. Once a DNA molecule is detected, the system performs the pulling experiment (step 6). When the experiment is finished, the system replaces the DNA particle by flushing the chamber with buffer solution and returning to step 4. The brightness and contrast of the microscopy images have been increased for greater clarity.

To achieve full autonomous operation, the SmartTrap is controlled by custom algorithms that use the readings from the various sensors for real-time feedback and to keep track of what stage of the experiment is being executed. This forms a closed-loop system consisting of four primary steps, as illustrated in Fig. 2a:

1. **Data acquisition:** Data are acquired by reading the various sensors (PSDs, photodiodes and camera) and the positions of the motors.
2. **Data processing:** The latest data are processed to extract the necessary information, such as the location of the particles, the locations of the lasers and whether a force is acting on the optically trapped particle. Image analysis is performed using the neural networks as illustrated in Fig. 2b.
3. **Control logic:** The processed information is used by the host computer to detect events (for example, a particle entering the trap), enabling it to decide what to do next based on which part of the experimental procedure is being performed, as illustrated for the case of a DNA pulling experiment in Fig. 2c. The decision process is customized for different experiments but many building blocks remain the same (for example, trapping of particles, alignment).
4. **Command execution:** Commands issued by the control logic are executed by the system, for instance to move the sample stage to a target position to trap a particle.

Importantly, the data acquisition and the command execution are performed asynchronously with the control logic by a combination of different threads and processes in Python. This makes it possible to sample and record data from the various sensors, including the camera, at frequencies higher than what the networks can analyze in real time.

When run on a computer with a Nvidia RTX 3090 graphics card, the decision process, including the image analysis, typically operates at about 20 Hz, which is sufficient for real-time feedback and comparable to the camera (Basler a2A5320-23umBAS) frame rate of around 23 frames per second when recording at full frame. The vast majority of the computational time in each decision is spent analyzing the images. Therefore, the feedback rate will depend on the field of view and how many particles are visible, as more particles in view mean that the *z*-network needs to perform more predictions. We also found that a large proportion of the analysis is due to latency in sending images and results to and from the graphics processing unit by observing only a 50% increase in processing time for the YOLO network when two images in a batch are processed compared to one. The process is sufficiently fast to autonomously perform experiments and much faster than the typical human reaction time^26^.

### Particle characterization

Optical tweezers are well suited for precision sorting because they can manipulate diverse samples in a non-contact manner and provide a variety of input signals about the trapped object. Here, we present an event-driven approach that rapidly characterizes particles using real-time image analysis and force measurements. We use a mixture of two particles, where we identify the particles of one type using real-time image analysis and measure their hydrodynamic radius.

The mixture consists of particles with two different size distributions: particles with *r* = 2.12 μm ± 0.05 μm (MicroParticles PS-R 4.2, PS/Q-R-B1198) and particles with a radii range of 1.0−1.5 μm (Spherotech SVP-20-5). The particles are washed before use, and their concentrations adjusted to obtain a ratio of about 5:1 (small:large). The larger particles are then selected with the help of the bounding boxes obtained from YOLO, and their hydrodynamic radius is measured using the Stokes drag method described in ‘Calibration’ in Methods, moving trapped particles at constant speed in the sample using the motors while measuring the force. To calculate the hydrodynamic radius, we use equation (2) from Methods.

To perform this experiment, the SmartTrap is instructed to autonomously follow these main steps:

1. **Trap a particle:** Trapping a particle begins by positioning the fluidics chamber using the motors so that the appropriate capillary tube opening is near the optical trap. The microfluidic pump is then used to create a flow of particles from the capillary into the main channel. Once a particle comes into view, the motors move to position the optical trap on the particle. This happens in a loop, so if the particle moves due to the flow from the capillary, the target position is updated accordingly. If there are several particles in view, the closest one is chosen. Once a particle is trapped, the flow is turned off and the trapped particle is brought back to the pipette.
2. **Select large particles:** If the trapped particle is smaller than a predefined threshold, the system immediately proceeds to step 4 to release it. The size is obtained from the prediction of the YOLO bounding box. This is checked after trapping the particle, since determining the size of particles far from focus is challenging.
3. **Measure the hydrodynamic radius:** The sample is moved between predetermined positions using the motors. This drags the trapped particle through the fluid and generates a drag force. From this force, the hydrodynamic radius is calculated using equation (2). The relative fluid velocity is determined from the recorded motor movement speed.
4. **Drop the particle:** The trapped particle is released by flushing the central chamber with a strong flow. The system then returns to step 1 to repeat the process and measure the radius of another particle.

**Fig. 3:**
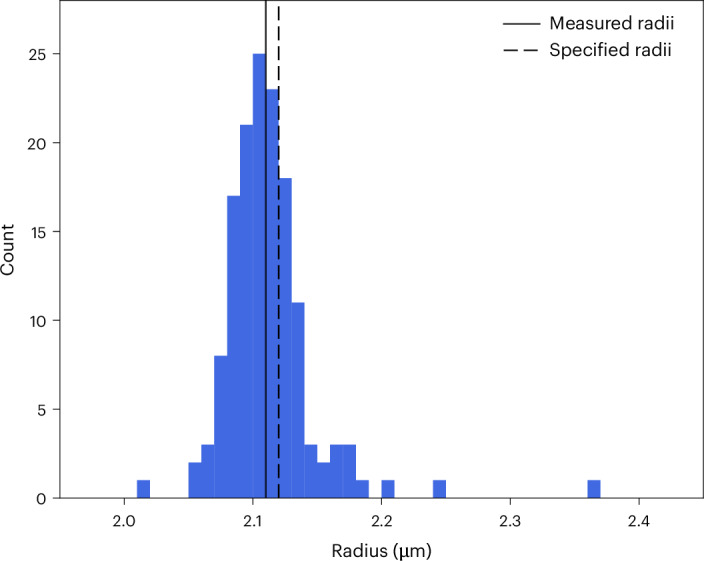
Size distribution of particles. The hydrodynamic radius was measured to be 2.11 μm ± 0.04 μm where the error is the standard deviation of the measurements. The average radius (solid line) and the manufacturer’s specified value (dashed line) are shown in the histogram.

We tested the SmartTrap with this protocol and had it run continuously for 4.5 h. During this time, 938 particles were trapped, 159 of which were large. Of these, 15 measurements failed due to trapping more than one particle, leaving 144 for the analysis. The results of the analysis are shown in Fig. 3. The autonomous protocol was terminated when the system ran out of particles.

There is good agreement between the measured radius of 2.11 μm ± 0.04 μm and the manufacturer’s specification of 2.12 μm ± 0.05 μm, demonstrating that the selection algorithm effectively separates the larger particles from the smaller ones. The selection is fast and typically takes less than a second. If the trapped particle is not correct, it is immediately dropped. The total throughput depends on the parameters of the system (duration of measurement and concentrations of particles being key parameters); in the measurement reported in Fig. 3, more than 200 particles per hour were investigated and the hydrodynamic radius was measured for about 30 particles per hour.

### DNA pulling

Understanding the structural and mechanical properties of biomolecules is essential to mapping their roles in biological systems. Single-molecule techniques have been transformative in probing transient states—such as protein and nucleic acid folding—and in revealing physical properties (for example, stiffness and binding interactions) typically obscured in bulk measurements, thereby illuminating processes like DNA replication, transcription and chromatin organization^27–30^. Single-molecule force spectroscopy experiments specifically reveal how molecular conformations change in response to an applied force. Optical tweezers are especially well suited for these measurements because of their high spatial and temporal resolution. Among the most fundamental of such experiments is DNA pulling, where the extension dynamics of a double-stranded DNA (dsDNA) molecule is measured as force is applied. This experiment helped establish that DNA can be accurately modeled using the extensible worm-like chain (WLC) model^31,32^. Although the details vary across different experiments, the core steps—tethering the molecule between two particles, controlling the distance between them and measuring the force as a function of extension—are shared by many single-molecule experiments, making the DNA pulling assay a typical force spectroscopy measurement.

**Fig. 4:**
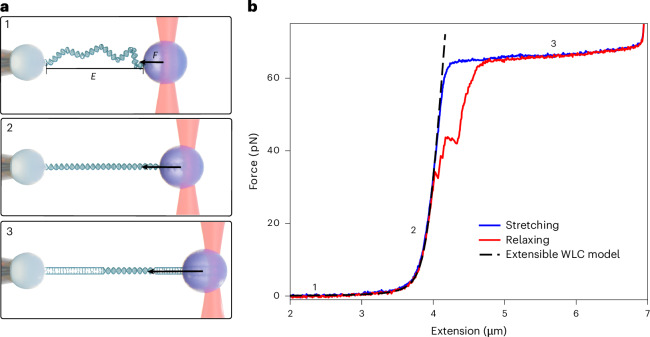
DNA pulling experiment. **a**, Illustration of a DNA pulling experiment. The DNA molecule is attached between two particles. One of the particles is kept fixed by a micropipette, while the other is moved back and forth using an optical trap. **b**, Force–extension curves from the experiment (solid lines) and the extensible WLC model (dashed line). Both the stretching (blue line) and relaxation (red line) of the molecule are shown. The measurement is aligned with the model to have the same extension when the force is 30 pN. The stretching and relaxation mostly overlap, apart from where there is hysteresis showing as an early drop of the force during the relaxation. At small extensions, the molecule exerts a low force and is slightly coiled (panel 1 in **a**). As the extension approaches the contour length of the molecule, the molecule straightens out causing the force to increase sharply with the distance (panel 2 in **a**); this behavior is well described by the extensible WLC model. At approximately 65 pN, the molecule overstretches (panel 3 in **a**). The extension of the molecule is calculated using the optical trap stiffness as outlined in the DNA pulling section of ‘DNA pulling experiment’ in Methods.

A DNA pulling experiment is performed by tethering a DNA molecule (in our experiment, a fragment of a *λ*-DNA molecule with a length of 4 μm) between two particles and measuring the force acting on the DNA molecule while the particles are pulled apart (Fig. 4a). One particle is held still by a micropipette while the optically trapped particle acts like a force probe while also being moved back and forth to stretch the molecule.

The automated DNA pulling experiment starts with a configuration routine executed by the user (step 0 in Fig. 2c). First, the user indicates to the software the positions of the pipette and the side capillaries by manually locating their openings in the chamber. These positions are saved with a single click as motor encoder positions. The user then needs to indicate which channel of the pump is connected to which channel of the fluidics chamber; this tells the system which pump channel to activate to start the flow of particles of a specific type.

Once the initial configuration is finished, the automated DNA pulling experiment consists of six steps, as illustrated in Fig. 2c:

1. **Check pipette and trap:** The algorithm determines whether a particle is present in the optical trap or in the pipette by checking if any particle is located within a prescribed distance of the estimated laser position or the pipette tip, respectively. Assuming that it is the start of the procedure, there will be nothing in either the trap or the pipette.
2. **Trap streptavidin particle:** The program traps a streptavidin-coated particle, which will be held by the pipette. This is done in the same way as in the particle characterization experiment, but importantly the system goes to the capillary connected to the channel with streptavidin-coated particles and brings the trapped particle back to the pipette.
3. **Suck into pipette:** With a streptavidin-coated particle trapped and positioned near the pipette, the next step is to catch it with the pipette. The tip of the pipette is located using the YOLO algorithm. The trapped particle is then approached to the tip, first using the motors for rough alignment, and then the wigglers to finely adjust the laser position. Once the particle is within a few microns from the tip, the pump connected to the pipette is briefly turned on creating a flow into the pipette and sucking the particle firmly into the tip. Thereafter, the program goes back to the saved position near the pipette to ensure that the particle is now in the pipette; if it is not, it will try again.
4. **Trap DNA particle:** Next, the particle with DNA molecules is trapped using the same method as for the first streptavidin-coated particle but instead going to the capillary containing the particles with DNA.
5. **Attach DNA:** Once a streptavidin-coated particle is in the pipette and a DNA particle is trapped near the pipette, it is time to get the DNA to attach to the streptavidin-coated particle. This is done by first positioning the trapped particle above the one in the pipette using the motors and position feedback. Next, the system aligns the focus of the two particles by adjusting the *z*-position of the chambers so that the *z*-positions of the particles, as predicted by the convolutional network, match. Then, they are aligned, also in the *x*-direction (perpendicular to the pipette) with the help of the wigglers, which provide superior precision to the motors.
  After this initial alignment, the particle in the trap is gently approached to the one in the pipette using the wigglers. This is done until there is a weak repulsive force between the particles (about 5 pN), indicating that the trapped particle is being pushed out of the trap by the streptavidin-coated particle in the pipette. Thereafter, the particles are separated, also using the wigglers. If things go well, then one DNA molecule on the trapped particle will have attached to the streptavidin-coated particle in the pipette.
  To check whether a molecule is attached, the two particles are separated and, if there is a force at large particle–particle separation, this indicates that a molecule is attached. For the cut *λ*-DNA molecules, this means a force of at least 60 pN at a distance of 4 μm or more. This is because we expect a force plateau at approximately 65 pN for extensions greater than 4 μm where DNA overstretches^31^. These numbers can be adjusted to better suit other molecules, providing an easy pathway to automate other similar experiments. Also, if the system detects a force along a direction other than the pulling direction (which can occur if the molecule does not bind to the top of the particle in the pipette), it will adjust the particle position to reduce this force and ensure a straight molecule extension.
6. **Perform experiment:** Finally, a pulling protocol is loaded onto the microcontroller. This protocol is a set of instructions that are repeated by the controller: moving the optical trap between two positions at a set speed to pull the molecule. The protocol is run on the microcontroller because it can update the trap position at a rate of 7 kHz, much faster than the communication with the host computer, ensuring a smooth motion of the trap. To find the upper limit of the separation, that is, the maximum distance between the particle in the pipette and the optically trapped particle, an upper force threshold is set, while a minimum separation distance between the particles is set to a specific value. Once this pulling protocol starts, the host computer also starts recording data. The protocol will repeatedly separate the particles to stretch the molecule and thereafter let it relax. This is repeated for a user-specified duration of time, which we have set to 10 min.
  If the molecule, or one of the tethers, is broken before the set time has elapsed, the protocol and data recording will be stopped and the system will revert to trying to attach a molecule. If the molecule does not break, the protocol will stop automatically when the time limit is reached. The particle is then removed by flushing the central chamber with buffer solution for a few seconds and the experiment is repeated with another particle. Because there are no molecules attached to the streptavidin-coated particle, it does not need to be replaced. However, if the flow is sufficiently strong, the streptavidin-coated particle may also be lost, in which case it is replaced by a new streptavidin-coated particle. To reduce the risk of this happening, the pipette pump is turned on while the chamber is being flushed.

Supplementary Video 3 demonstrates how these steps work in practice.

In addition to the general procedure described above, there are also continuous checks to detect if something goes wrong throughout the experiment and autonomous procedures to handle such situations. For example, not all particles have DNA attached, which is why the program will only try to attach the DNA molecules a limited number of times (ten) before trying with a different particle. If instead two DNA molecules get attached, this will show up in the subsequent analysis as a sharper increase in the force than expected, but it does not otherwise change the procedure and is dealt with in the data analysis after the experiment is finished. The presence of more than one particle in the trap is detected by checking if the predicted *z*-position deviates from what is expected from a single particle. The program also checks that the trapped particle is not lost throughout the experiment. These are rare events, which the system handles by returning to the starting state and, in the case of double-trapped particles, by also dropping what is in the trap and flushing the central channel to remove excess particles.

**Extended Data Fig. 5:**
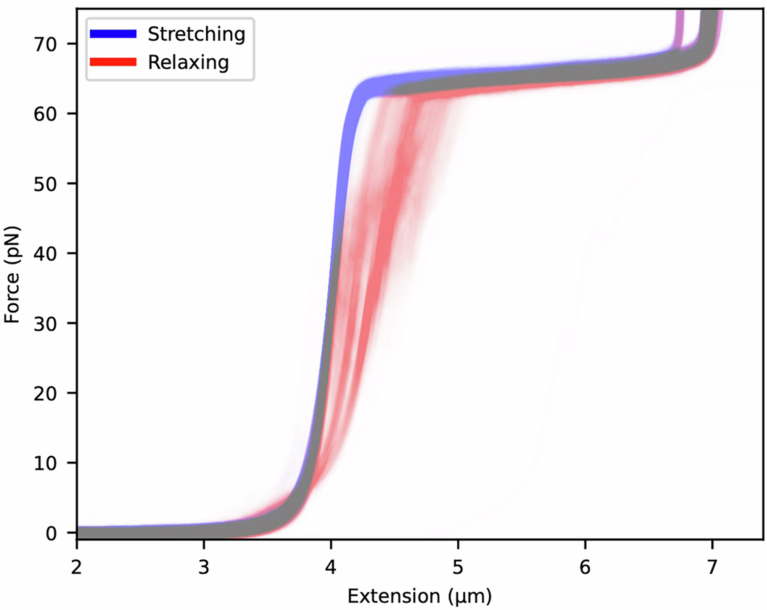
Force-extension curves from autonomous experiments. The blue curves show the stretching and the red ones show the relaxation of a DNA revealing hysteresis due to nicking, especially in the 4 to 5 *μ*m extension range. The curves are plotted with high transparency to enable comparison. In total, there are 565 different curves from 16 different molecules aligned to have the same position when the force is 30 pN. These curves are all obtained from the same experiment session and include the experiments in which a single DNA of length 4 *μ*m is attached and tethers do not break before reaching the overstretching plateau.

The number of experiments performed per hour depends on the specific experiment with the likelihood of molecule attaching, particle concentrations and pulling rate having a large influence. It typically takes around 3 min to release the previous particle, trap a new one, align it, attach a molecule and start performing the pulling protocol. We ran the DNA pulling protocol until failure to test its ability to collect large amounts of data (Supplementary Video 3). We found that it can run for up to 10 h without supervision, mainly limited by the stability of the particles and the evaporation of the objective immersion water. During this time, 29 different molecules were tested. The results of this are shown in Extended Data Fig. 5. The frequency of successful experiments gradually decreased with time, which we attribute primarily to particle stability and to a slow decay of the biomolecules at room temperature.

**Extended Data Fig. 6:**
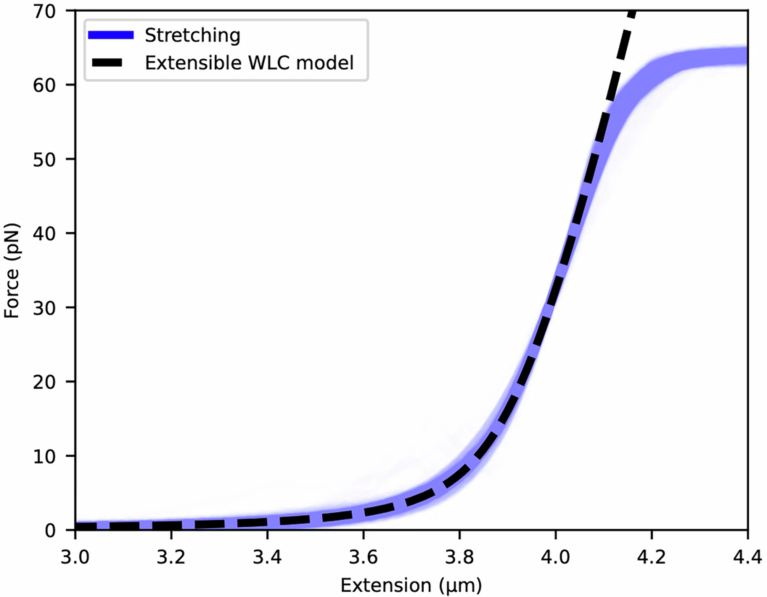
Force-extension curves from autonomous experiments compared to the WLC model. Comparison of the extensible WLC model, with lp = 43 nm and K0 = 1200 pN, and the 565 different stretching curves from Extended Data Fig. 5. The overall agreement is excellent, with minor deviations we attribute to hysteresis caused by nicking of the molecules visible in a few curves also at low forces for which the molecule has not re-annealed before the next pull is initiated.

The experiment yields the force as a function of the extension (Fig. 4b). To estimate the extension, we use the position PSD detectors and the optical trap stiffness (see ‘DNA pulling experiment’ in Methods). Up until the overstretching, the force–extension curve can be modeled by an extensible WLC model (equation (3)). In Fig. 4b, we see that there is excellent agreement between the model and the data. Because the analysis gives the particle’s position rather than molecular extension, and as we do not know the precise molecular attachment point on the fixed pipette particle, our data have been offset to align with the model and give the same extension for a force of 30 pN. There is also some hysteresis when the particles are moved back due to force-induced melting of the molecule. Hysteresis is seen as early drops in the force curve. Lastly, there is a force plateau at about 65 pN where the molecule overstretches. The plateau is approximately 2.8 μm long, corresponding to 70% of the contour length of the molecule, which is in good agreement with previously reported results for *λ*-DNA^31^. The results were also consistent between different molecules as shown in Extended Data Fig. 5 and the comparison to the WLC model in Extended Data Fig. 6, which contain traces from more than 500 curves.

### Stretching of red blood cells

The mechanical properties of red blood cell membranes are vital to their biological function, as these cells must deform to navigate through narrow capillaries and withstand the shear forces present in blood circulation^33^. Changes in membrane stiffness are associated with conditions such as sickle cell disease and malaria^34^. Moreover, because red blood cells lack most internal organelles and cytoskeletal structures, relatively small forces can induce measurable deformation, making them highly sensitive probes for mechanical studies^35,36^. Therefore, understanding how red blood cell membranes respond to force not only provides insight into fundamental biophysical processes but also has clinical implications for diagnosing and treating diseases linked to abnormal cell rigidity.

To stretch red blood cells, we exploit the fact that the momentum of light changes when light passes between two media with different refractive indices, such as the interior of a cell and the surrounding buffer. This gives rise to a force acting on the interface that is directed normally to it and away from the denser media. For a trapped cell, the force is directed away from the cell membrane both when the light enters and exits the cell. By trapping using various laser powers, we are able to see a clear difference in the shape of the cells. The higher the trapping power, the more elongated the cells are along the propagation axis, giving them a smaller cross-section when viewed from the camera. Our approach is similar to that used in the optical stretcher device^36^.

**Fig. 5:**
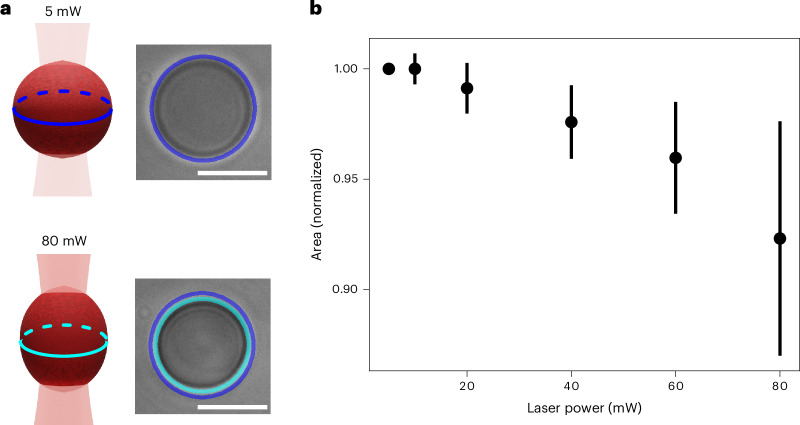
Optical stretching of red blood cells. **a**, Illustrations of a trapped red blood cell. When the trapping power is 5 mW per laser, there is no measurable stretching, and the cell is near spherical due to the low osmotic pressure. To the right, there is an experimental image with the dashed blue line showing the outline of the cell. When the cells are trapped with 80 mW per laser, they stretch along the propagation axis of the laser. The experimental image in the bottom right shows an outline of the same cell as above when trapped in low power, highlighting that the cell has shrunk in the transversal plane. Scale bars, 5 μm. **b**, The cross-sectional area of the cells gradually decreases with increasing trapping power corresponding to their shape becoming more prolate. The error bars show the standard deviation of the relative areas; 18 red blood cells from the same sample were used with an average cross-sectional area of 39 ± 4 μm^2^ as measured when trapped at 5 mW power. The units are normalized by the size of the cells when trapped at 5 mW. The brightness and contrast of the microscopy images have been increased for greater clarity.

For these experiments, human red blood cells are diluted in a low-osmolarity buffer, making them inflate and become nearly spherical. The solution is then flown into the microfluidic chamber of the SmartTrap where the red blood cells are trapped. Initially, the lasers are set to a low power (approximately 5 mW in the sample from each laser). Then, the power of the two traps is changed simultaneously in steps up to 80 mW. Trapping at low power establishes a baseline at which the cells are almost perfectly spherical. At higher laser powers, this force is greater, which stretches the cells more, decreasing their cross-sectional area; this stretching is illustrated in Fig. 5a.

These experiments are comparatively easy to automate since they involve only a single cell and therefore do not make use of the micropipette. The SmartTrap only needs to trap a red blood cell and then record its profile while the trapping power is changed. The procedure can be split into four main steps (Supplementary Video 4):

1. **Flow cells into the chamber:** The flow in the central channel is briefly turned on, with the aim of bringing cells into view and removing cells from previous measurements from the optical trap.
2. **Look for cells:** SmartTrap looks for cells within the field of view. If there are no visible cells, it reverts back to step 1 to flow in more cells.
3. **Trap a cell:** With red blood cells in view, the system proceeds to trap the closest one. The laser power is set to 5 mW so as not to deform the cells during trapping.
4. **Measure cross-section:** The trapping power is changed in steps while the transversal profiles of the cells are recorded in separate videos, one for each power for subsequent analysis. Once the measurement is finished, the system goes back to step 1. By briefly turning on the flow in the central channel, the system guarantees that the same cell is not measured twice.

Performing measurements on a single cell takes 2 to 3 minutes. Because the cells are nearly spherical and homogeneous, they are very similar in appearance to the particles used in the DNA experiment, with the primary differences being a lower refractive index and greater size. Therefore, by just including a few dozen cells in the training data, the YOLO algorithm is able to detect them. To quantify the stretching, the cross-sectional area of the cells is monitored by the camera and measured using standard image analysis techniques, specifically a Gaussian filter followed by a threshold that extracts the area of the cells.

As shown in Fig. 5b, the cells contract markedly with increasing laser power. Our results and those of Guck et al.^36^ are of similar magnitude for the same laser power; a direct comparison between different stretching methods is difficult due to the different trapping geometry. We also note that the cross-sections of the cells decrease continuously with increasing laser power.

### Electrostatic interaction between particles

In colloidal sciences, it is essential to measure the interaction forces between particles (for example, electrostatic, Van der Waals and hydrodynamic interactions) to understand phenomena such as self-assembly, adsorption and aggregation^37,38^. These interactions have broad industrial applications, for instance, in the food industry, pharmaceutical industry and water treatment. There are multiple methods for assessing these interactions, many of which look at bulk solutions, such as dynamic light scattering and electrophoretic mobility measurements. Optical tweezers enable studying these interactions at the single-particle level and on the same particles in different conditions (for example, temperature, salinity, pH). Electrostatic interactions, in particular, are central to stabilizing colloidal suspension by preventing particles from coming close enough to aggregate^37^.

**Fig. 6:**
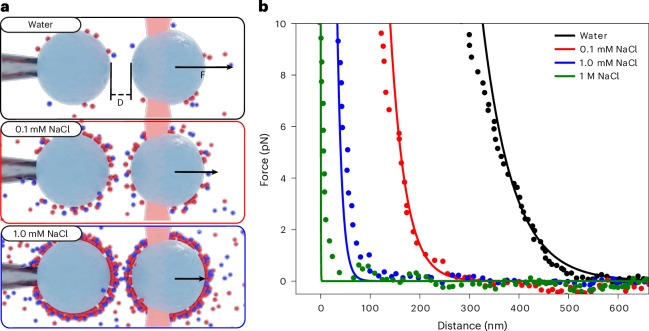
Electrostatic interactions between particles. **a**, Depiction of the electrostatic interaction experiments across varying salt concentrations. As the salt concentration increases, so does the screening that reduces the electrostatic force, illustrated as more ions aggregated around the particles. **b**, Force as a function of particle–particle distance for various salt concentrations. The experimental data (dots) agree well with a fit of the electrostatic force as described by Derjaguin–Landau–Verwey–Overbeek (DLVO) theory (solid lines).

To measure the electrostatic repulsion between particles in an optical tweezers, two particles are brought close to each other, and their positions and the force acting on them are measured simultaneously at varying distances. The configuration we use, as shown in Fig. 6a, is similar to that used in ref. ^39^. Because the particles carry a small stabilizing charge from the sulfate end groups on their surfaces, the electrostatic force will repel the particles from one another at short distances. By repeating the process to measure this repulsive force as a function of distance, we map how this interaction changes across a range of different salt concentrations.

SmartTrap can autonomously measure electrostatic repulsion between multiple particle pairs in a single medium. The protocol used for this is similar to that of the DNA pulling, with steps 1–3 being the same, but with some key differences in the later steps related to the measurements:

1. **Check the pipette and trap:** See step 1 in the DNA pulling protocol.
2. **Trap a particle:** See step 2 in the DNA pulling protocol.
3. **Suck into pipette:** See step 3 in the DNA pulling protocol.
4. **Trap the second particle:** A second particle, of the same type as the first, is trapped and brought next to the particle already in the pipette.
5. **Align the particles:** The trapped particle and the one in the pipette are aligned by ensuring that they are in the same focal plane and have the same *x*-position. The alignment is similar to that performed in the DNA pulling experiment when attaching DNA.
6. **Measure the repulsion:** The particles are pushed together until the force exceeds a certain value, which we set to 10 pN. This is used as the first endpoint of the protocol. The second endpoint is set to a little less than a micron away from the first where the electrostatic force is negligible. Also, the lasers are moved at a speed of less than 10 nm s^−1^ to avoid any hydrodynamic effects from the particles moving close together.
  Once finished, the system will flush the central chamber with a strong flow to clear both the pipette and trap from particles.

Supplementary Video 5 shows this protocol in action. When comparing results across different salt concentrations, it is crucial to use the same particle pair. Variations in particle size and surface charge would otherwise make measurements incomparable, given the short range of electrostatic forces. Manually closing off the channels and replacing the solution without losing the particles ensures the integrity of the experiment. Our autonomous system still plays a vital role in real-time tracking, force measurements and three-dimensional (3D) alignment, while also helping to determine appropriate flow rates for solution replacement.

**Extended Data Fig. 7:**
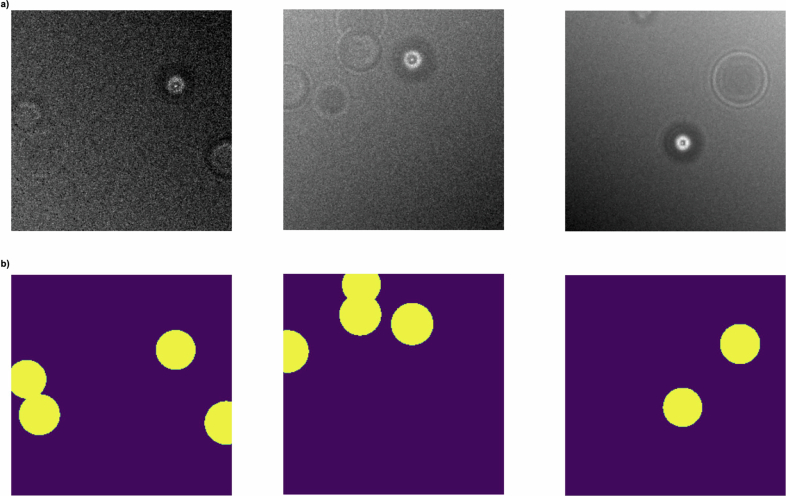
U-Net training data. (**a**) Simulated images generated using the DeepTrack2 Python package and used for training the U-Net. Small particles are added to the background and used as noise to prevent false positives in the output (for example, classifying the pipette as a particle). (**b**) The targets are binary masks with the same size as the images. The U-Net is tasked to predict 1 for pixels containing particles (yellow circles) and 0 otherwise.

We start with distilled water and incrementally increase the salt concentration by flushing the chamber for 30 min at each step. The ions from the salt screen the electrostatic interaction, meaning that for a certain distance the repulsive force will be lower at higher salt concentrations, as illustrated in Fig. 6a. In this experiment, the two particles get very close to one another, a situation that is challenging because the images of the particles overlap, making standard methods fail^40^. We found that the YOLO algorithm yields results that are too noisy in this case. Because of this, the particles are tracked using a U-Net, which has been trained on simulated data (Extended Data Fig. 7) and specifically to handle the case of particles being very close. See ‘U-Net model for accurate particle tracking’ in Methods.

From Fig. 6b, we see good agreement between experiments and theory. It also becomes apparent why it is essential to use the same pair of particles to see the differences; the difference between high and low salinity is largely that the interaction appears at a longer distance and for salt concentrations above around 0.1 mmol this distance is on the order of 100 nm, less than one-twentieth of the particle diameter. The increase in the force is not as sharp as theory predicts for the two highest salt concentrations. We attribute this to the sulfate end groups on the particle surfaces contributing with a slight repulsive force.

## Discussion

Measuring forces and mechanical properties with optical tweezers is still largely a manual process, limiting the amount of data that can be collected and potentially introducing human biases. SmartTrap represents a substantial step toward overcoming these limitations. The experiments presented here cover a wide range of common optical tweezers applications. Particle characterization, although relatively simple in our demonstration, has a wide range of potential applications (for example, selecting certain types of cells on which to perform experiments) that could be readily implemented by modifying the selection criteria from size to another feature. Also, since the force measurement is independent of object shape, it can be used to measure forces on nonspherical objects such as bacteria or plankton. DNA pulling serves as a paradigmatic single-molecule force spectroscopy experiment. With some parameter tuning, it could be adapted to measuring other molecules (for example, proteins, DNA hairpins). It would then be ideal for mapping the energetics of molecular processes across different conditions. One could, for instance, measure DNA nearest-neighbor energies for various temperatures or salinities. This type of high-accuracy experiment is central to understanding molecules and requires a large number of measurements^16,41^. In contrast, the red blood cell experiment proved easier to automate than DNA pulling, yet clearly illustrates that one can, with modest effort, efficiently perform a large number of simple measurements on individual cells or particles. The electrostatic repulsion experiments, on the other hand, exemplify the measurement of interparticle forces. Notably, the algorithm used there is essentially a simplified version of that used for DNA pulling, illustrating how the steps building up a complex protocol can be repurposed for other applications. This makes subsequent automation protocols quicker and easier to implement.

Compared to a human operator, SmartTrap generally matches or exceeds manual operation in both measurements per hour and overall acquired data quality. We attribute this advantage to faster feedback and more precise measurements of relative positions. As the particle characterization experiment demonstrates, when measurements can be performed quickly, the system is capable of conducting a large number of measurements autonomously in a short period. The fact that the autonomous protocols can run reliably for an extended time, up to 10 h as shown by the DNA pulling experiments in Extended Data Figs. 5 and 6, greatly reduces the time needed by researchers to perform experiments. Furthermore, having the instrument working on its own also reduces the risk of human bias from inconsistent operation, which is potentially very important when trying to observe rare events. Still, there are scenarios in which human operators still outperform the system, such as detecting particles that are out of focus or moving very rapidly. These situations benefit from detecting motion by comparing consecutive frames. YOLO operates on a frame-by-frame basis and thus struggles with fast movement and identifying particles moving far from focus, particles which are detectable to humans. It is likely that a more specialized tracking algorithm could outperform humans also in these scenarios.

Many of the challenges faced during automation stem from having one of the particles held in the pipette, which introduces an additional point of failure and adds complexity by requiring precise 3D alignment. This requirement largely explains why preparing a new measurement is faster in the particle characterization experiment than in the DNA pulling experiment, even when the particle in the pipette is not replaced. An alternative approach would be to use two separate traps to ensure both particles remain in the same plane, effectively removing the need for extensive alignment. However, this comes at the cost of increased instrument complexity and would likely require trapping two particles for each subsequent measurement. Nonetheless, extending the automation processes to double-trap optical tweezers or holographic optical tweezers is quite straightforward. Considering that the pipette is a mechanical trap and the SmartTrap already uses two counterpropagating laser beams, adapting from an instrument with an optical and mechanical trap to a system with two optical traps requires no major software modifications.

Despite these constraints, errors during autonomous operation are rare, as demonstrated by the system’s ability to perform both extended measurements and large-scale studies on numerous particles, molecules or cells. This reliability is primarily due to solutions already in place for common problems, such as trapping two particles, losing the particles or failing to attach DNA. When an issue does occur, it usually stems from external experimental factors that require human intervention, such as running out of particles or the pipette clogging. These issues are relatively simple to fix but currently require manual intervention.

Looking further ahead, the trend of handing over repetitive work to computers is likely to continue. As the breadth of the autonomous protocols we have implemented demonstrates, large parts of protocols can often be reused in other protocols, greatly lowering the barriers to automation. Although our system is an optical tweezers, several building blocks of the autonomous functionality could be used to perform other smart microscopy experiments. For instance, the trapping of particles, seemingly highly specific to optical tweezers, works by actively centering particles in the field of view. Meaning that, without modification, it could be used to follow the path of single particles and, by retraining the YOLO network, as we did for the red blood cells, it could instead follow single plankton or bacteria in a sample. As the technique matures, a wider range of experiments will be automated. Furthermore, on our GitHub we provide multiple automation subroutines, such as particle trapping and alignment, which simplifies development of future autonomous protocols^42,43^. Nonetheless, it is unlikely that humans will be entirely removed from the process anytime soon. Especially in the early stages when starting a new experiment and designing protocols, manual operation is still necessary before handing control over to the computer. However, we have found that some of the functions of the autonomous system, such as automatic alignment of particles and real-time tracking in 3D, are very useful, also when manually testing or performing new protocols. It is therefore likely that such hybrid modes of operation will become the norm, assisting researchers in both established and novel experimental endeavors.

## Methods

### Optical tweezers system

The optical tweezers system used is a counterpropagating tweezers inspired by the MiniTweezers design originally developed by the group of C.B. at UC Berkeley^44^. Importantly, we have designed our own custom circuit boards, firmware and GUI to gain full digital control of the instrument while keeping the same optical system as the original design. The instrument was designed for single-molecule force spectroscopy studies and can trap particles down to 0.5 μm in diameter. It has a small footprint of approximately 30 cm across, which allows it to be easily moved and placed within environmentally controlled chambers. Assembly instructions and a component list for the optical tweezers instrument are available from our GitHub and Zenodo repositories^42,43^. Having digital control of all the components of the system in a single software proved essential for automation. In addition, the code and schematics for the electronic used to control the system are available from the repository.

#### Optical paths

The two lasers (both Lumics lasers, model LU0808M250) follow equivalent but mirrored paths to form the counterpropagating optical trap, as shown in Fig. 1b.

Here, we describe the path of laser A. Laser A exits from an optical fiber into wiggler A, which controls the axial position using a piezoelectric actuator. The function of the wiggler is described in detail in the ‘Laser wigglers’ section. Next, a small portion (about 8% of the laser power) is deflected by a pellicle beam splitter and focused onto position detector A. This is a PSD used to measure the laser position. The remainder of the laser light is collimated by a lens and passes through a quarter waveplate before entering the back aperture of the objective. The quarter waveplate ensures that the light is circularly polarized when entering the objective. The objective focuses the laser inside the sample, creating the optical trap. After passing through the sample, the laser is collected and collimated by the second objective before passing a second quarter waveplate, which again linearly polarizes the light. Both objectives are the Olympus UPlanSAPO ×60, water immersion, with an NA of 1.2. Importantly, the laser is now polarized so that it is transmitted through the first polarizing beamsplitter and reflected off the second. Finally, laser A is focused by a lens onto force detector A (also a PSD). This PSD is positioned in a pivot point relative to the center of the sample ensuring that the laser can be moved in the sample without giving a reading on the force sensors. In this way, the SmartTrap achieves a force resolution of 0.04 pN and a distance resolution of 1 nm when using the PSD sensors and about 10 nm when using video microscopy.

There are also an iris and a photodiode that are used to measure the intensity at the center of the beam. When a particle is trapped in a neutral position, the ratio between the light collected by the photodiode and that collected by the PSD is the same as when there is nothing in the trap. If a trapped particle moves along the *z*-direction (left and right in the schematics), the particle will act as a lens. Depending on the direction of the displacement, it will either focus or defocus the laser on the photodiode through the iris. This allows for the measurement of momentum changes in the axial direction, which after calibration can be used to calculate the force along the *z* axis^45^.

Laser B follows an equivalent but mirrored path as laser A.

The sample is imaged using a brightfield configuration with a blue LED and a short-pass filter positioned before the camera to filter out any stray light from the lasers.

#### Force measurements

The primary reason for using counterpropagating beams is that it allows for direct force measurements^21^. This is achieved by monitoring the momentum change of the lasers as they pass through the sample. Any change in momentum of the lasers as they pass through the sample will be caused by objects in the trap. By Newton’s third law, this momentum change gives rise to a force on the trapped object. Importantly, all the scattered laser light needs to be collected to get an accurate reading of the momentum change and thereby the force. This is achieved by not overfilling the back apertures of the objectives (that is, only a small portion of the back aperture is covered by the laser). Because of this, large-angle scattering does not occur, which means that the objectives are able to collect practically all the laser light passing through the sample. Therefore, the deflection of the lasers is directly proportional to the force acting on the particle.

As an additional benefit, using counterpropagating traps allows for lower numerical aperture on the objectives and longer working distances making it easier to work in the bulk of the solution and thereby avoiding surface effects during measurements.

#### Laser wigglers

To move the lasers in the system, a piezoelectric mechanical system called a wiggler is used. The system is described in US patent number 7274451 B2 (ref. ^46^). The wiggler consists of a fiber placed inside two metallic tubes, one inner and one outer slightly longer tube. The outer tube has a metal ball attached to it. The system works by having piezoelectric crystals push on a metal ball, which in turn moves the outer metal tube relative to the stationary inner tube, thus gently bending the optical fiber. This provides a simple yet efficient method for moving both lasers, allowing for accurate positioning of the lasers and fast feedback algorithms for synchronous movement of the two lasers in the sample.

#### Microfluidic chamber

Many experiments require two particles to study interactions. To get two particles, a custom microfluidic chamber with a micropipette is used. The chamber has three channels: one central channel for performing the experiments and two side channels through which the functionalized particles are flowed in. These side channels are connected to the central channel by glass capillaries through which particles can flow. These glass capillaries have an outer diameter of 100 μm with an inner diameter of 25 μm and are cut to the appropriate length using a scalpel. In the illustration shown in Extended Data Fig. 1a, the particles flow from left to right when the pumps are turned on.

The chamber is handmade and consists of two sheets of parafilm and two glass slides. When making a chamber, the two parafilm sheets are first cut into the appropriate size using a laser cutter. Simultaneously, the laser cutter cuts out the channels, giving the parafilm the shape seen in Extended Data Fig. 1a. Holes are also cut with the laser cutter in one of the two glass slides to make inlets and outlets. Next, one of the sheets of parafilm is placed on the glass slide with holes. The holes are aligned with the channels and the capillaries and the micropipette are positioned on the parafilm. The second sheet of parafilm is added on top followed by the second glass slide before the chamber is sealed by warming the parafilm close to its melting point. The micropipette is made from glass capillaries with an outer diameter of 80 μm and an inner diameter of 40 μm using a custom capillary puller. The puller uses a platinum filament to heat the glass and a small weight to apply a consistent force to the capillary. The heating of the filament is tuned to give a pipette opening with a diameter of around 1 μm. Illustrations of the chamber assembly process and instructions for constructing the puller are available in the GitHub and Zenodo repositories^42,43^.

To control the flow in the three channels, a microfluidic pump system is used. The system is an OB1 from Elvesys with three independent pressure-controlled pumps. Each is connected to one of the three channels of the chamber to give dynamic control of the flows. Lastly, a separate pump is connected to the micropipette, which is a one-way air pump (D2028B, SparkFun Electronics) and provides motorized suction.

### Electronics

The electronics has been designed to take advantage of the widespread availability of powerful microcontrollers. An Arduino Portenta H7 Lite is used as a microcontroller unit because it is easy to obtain and program, requiring minimal prior experience and no custom equipment. This makes it straightforward to connect and program, requiring just a USB-C cable. The controller samples the various photodetectors, steers the laser wigglers and controls the motors (Thorlabs Z606 DC Servo Motor) that move the sample. The schematics of the electronics are, like the software, available from our GitHub and Zenodo repositories^42,43^.

#### Sampling of PSDs

All four PSD detectors are handled using the same type of circuit and they are all reverse-biased with 15 V. The signals are amplified in two stages. The first stage acts as a transimpedance amplifier, converting each of the four current signals from the detectors into voltages. The four signals correspond to *X*_1_, *X*_2_, *Y*_1_, *Y*_2_ (two for each axis). In the second stage, the differences *X* = *X*_1_ − *X*_2_ and *Y* = *Y*_1_ − *Y*_2_ as well as the sum of the *y*-axis signals *S* = *Y*_1_ + *Y*_2_ are obtained using standard amplifier subtraction and addition circuits. These three signals are then sampled independently and these give the power and position. The laser power is proportional to the sum signal *S*. The difference signals are proportional to both the displacement and the power of the laser and so is the force, meaning that the *X* and *Y* difference signals are directly proportional to the force along the corresponding axis. To get the laser position, independent of the laser power used, the positions *X*_p_ and *Y*_p_ signals are extracted as ${X}_{{\rm{p}}}=\frac{{X}_{1}-{X}_{2}}{S}$ and ${Y}_{{\rm{p}}}=\frac{{Y}_{1}-{Y}_{2}}{S}$. It is possible to perform this division operation using analog circuitry but in order to limit the complexity of the circuit design it is done digitally in our system. The photodiodes used for measuring the *z*-force are also reverse-biased. Similarly to the PSDs, they generate a current signal that we convert to a voltage signal using a standard transimpedance amplifier circuit and sending the signal directly to the AD converter.

All the signals are sampled using a 16-bit 16-channel analog-to-digital converter (model AD7616BSTZ-RL from ANALOG DEVICES).

For controlling the piezoelectric actuators that steer the lasers, a high-voltage amplifier is used that yields an output in the range of 0–150 V, making 75 V the center position. The amplifiers are controlled by a digital-to-analog converter. The digital-to-analog converter is a four-channel 16-bit model, which is interfaced using the SPI protocol from the microcontroller allowing for fast and accurate control.

A motor driver circuit L293D provides the motors with the driving signal, and their direction is controlled with two digital pins. The Arduino Portenta uses software interrupts that trigger on the movement of the motors, which updates an internal counter, increasing it if the motor moves forward, and otherwise decreasing it. A proportional-integral-derivative feedback algorithm is used to get stable motor speed when moving.

### Software

A custom software suit has been designed and implemented to control the instrument. It can be broadly split into four main parts: the first is the firmware, which is written in C and runs on the microcontroller; the second is the communication software; the third is the GUI; and the fourth is the set of automation algorithms customized for each type of experiment (described in ‘Results’). The communications software, GUI and automation algorithms are all written in Python and run on the host computer.

#### Firmware

The firmware runs on the microcontroller and communicates with the instrument hardware. It reads from the analog-to-digital converter, writes to the digital-to-analog converter and controls the motors. The sampling of the PSDs is triggered every 64 μs by a software interrupt procedure resulting in a sample rate of 15.625 kHz. A USB-serial protocol is used to continuously communicate with the host computer through the built-in USB-C port of the Arduino Portenta.

#### Communications software

The communications software is what enables the host computer to read data from the instrument and send commands to it. Because this needs to happen continuously, it is important that the global interpreter lock of Python does not interrupt the process. Similarly, it is important that the communication does not interrupt any other processes, such as capturing images using the camera. Therefore, the communication is handled by two separate classes, both of which run asynchronously. The first class is run in a separate process (core on the computer) and sends data to and from the instrument using the serial USB protocol. It always sends the most recent command to the instrument and the data being read are continuously placed into a Python queue. The second class runs in a separate thread and processes data from the queue in chunks. First, the data are unpacked by converting from bits to integers and sorting the data depending on which signal it corresponds to. Thereafter, forces and positions are calculated; see ‘Calibration’ for further details.

#### GUI

The GUI is designed to control and monitor the communications with the instrument controller in a user-friendly manner. Enabling features such as clicking and dragging in the live view of the sample to position the lasers and to move the motors. We used a Basler ace a2A5320-23um camera, but any camera from this manufacturer will work without any modifications to the code, as will cameras from Thorlabs (other cameras can be integrated with minor modifications to the code). The GUI also works as a simple oscilloscope, enabling the user to plot and compare signals from the various sensors in real time. This is most readily used to monitor the forces on the particle during an experiment. The plotting tool also allows for real-time data processing such as running an FFT algorithm or averaging the data. The GUI also has features such as real-time tracking in the live feed as well as the ability to draw forces acting on the trapped particle. Examples of the live plotting and drawing of forces are shown in Supplementary Video 3, as well as a screen recording of the experiments. This can, for instance, allow users to identify where the DNA has attached on the particle in the pipette.

#### Automation algorithms

The automation algorithms are run in the background of the GUI in a separate thread taking care of steps 2, 3 and 4 of the automation loop in Fig. 2a. Running in a separate thread prevents other operations (for example, data image capturing) from having to wait for the automation algorithms when, for instance, a particle detection is performed. The user can decide which autonomous algorithm to run from the interface turning them on/off dynamically. Also, single procedures can be toggled individually, such as trapping a particle or alignment of the pipette, to help assist users when manually operating the instrument. The details of the various decision procedures are outlined in the main text.

### Automatic alignment of counterpropagating traps

In order for a counterpropagating optical trap to be stable, the two lasers need to be perfectly aligned in the sample. This can be very challenging if the optical trap is also to be moved, because any small difference in the optical path will cause the lasers to drift apart in the axial position when moved. Therefore, a feedback algorithm is used to keep the two lasers in the same axial position. This algorithm makes use of the fact that if the two lasers are aligned, and a particle is trapped, then they will have the same force reading. In practice, the force PSDs are set to zero in software when there is no particle in the trap. This compensates for the fact that the lasers may not be hitting the exact center of the sensors. Then, when a particle is trapped, if the beams are not perfectly aligned in the *xy* plane, the two beams will be deflected by the particle equally but in opposite directions. This is compensated for by the feedback, which moves the lasers to ensure that the PSDs give the same force reading as described in ref. ^21^. This feedback is run at around 7 kHz, meaning that the lasers follow each other very well also when moved, as during the DNA pulling protocol.

### Calibration

The calibration of the optical trap is essential for performing measurements with the tweezers, because it enables the conversion of voltage readings in bits to physical forces and distances. These conversion factors are given by the proportionality constants for the force PSDs and for the position PSDs. How these proportionality constants are obtained is outlined below. However, before these constants are obtained, a fundamental calibration is performed using a micrometer ruler mounted instead of the microfluidic chamber. This gives a calibration factor relating pixels on the camera to microns in the sample.

To calibrate the force PSDs, we exploit Stokes’ law, which relates the viscous drag on a particle to the flow velocity at a low Reynolds number^47^. Because the experiments are carried out in a microfluidic chamber, we need to account for the distance to the two walls, which is why we use the corrected formula from ref. ^48^ as given by equation (1):

1 $${\mathbf{F}}_{{\rm{d}}}=-6\pi \mu r{\mathbf{v}}\left(1+2\frac{9r}{16d}\right)$$

In this formula, **F**_d_ is the drag force, *μ* is the dynamic viscosity, and **v** is the velocity of the particle. The experiments are carried out in the center of the chamber, which has two walls separated by around 200 μm, which gives a value of *d* = 100 μm. In our case, the viscous drag is generated by moving a trapped particle back and forth using the stage motors. The drag force displaces the particle in the optical trap, which in turn gives readings on the PSD detectors, where *P*_r_ is directly proportional to the force, ${\mathbf{F}}_{{\rm{d}}}\propto {P}_{{\rm{r}}}$. The reading is directly proportional to the force because the change in laser light momentum is the same as the force exerted on the trapped particle, and the dual-lateral PSD measures transverse light momentum directly. Unlike single-beam traps using backfocal-plane interferometers, which cannot collect all the deflected light, our force calibration factor *P*_r_ remains constant regardless of changes in particle size, shape or refractive index^21^.

With the instrument calibrated, the Stokes drag method can be used to measure the size of particles. Then, equation (1) is solved for *r* which gives equation (2):

2 $$r=\frac{-1}{2b}+\sqrt{\frac{| {\mathbf{F}}_{{\rm{d}}}| }{ab}+\frac{1}{4{b}^{2}}},a=6\pi \mu | {\mathbf{v}}| ,b=\frac{18}{16d}$$

In the case of the particle characterization experiments, we use the tabulated value for *μ* for water at room temperature (21 ^∘^C) and 0.9775 mPa^49^.

We exploit the digital camera to calibrate the position readings of the lasers. This is done autonomously with a trapped particle, moving it in a square pattern using the wigglers while recording its position with real-time tracking. Then, the particle position is fitted to the signals, correcting for any differences in alignment or sensitivity of the detectors. It is important to note that the position signal is the laser position, rather than the particle position, and these may differ measurably when there is a force acting on the particle. Because of this, the position, as obtained from the camera, is sometimes preferred over the PSD readings, even though the sampling rate of the PSD signal is much greater than that of the camera. This analysis was performed using Python scripts utilizing the NumPy and SciPy packages.

### Artificial neural networks

Artificial neural networks are computational models that form the foundation of most modern AI algorithms. They get their name from their loose resemblance to biological neural networks^3,50^. There are multiple different artificial neural network models, referred to as architectures, which are adapted to various tasks. Here we provide a short outline of the networks used in the SmartTrap system to enable automation and highly accurate particle tracking. We also highlight that replacing or adapting the tracking methods, for example, to trap bacteria instead of spherical particles, is straightforward. One simply needs to implement the interface provided in our GitHub and Zenodo repositories^42,43^. The networks presented are available on GitHub, and on figshare via 10.6084/m9.figshare.31281967 (ref. ^51^).

### YOLO to predict lateral position of particles and pipette

A YOLO network is employed for object detection, specifically YOLO v5s, which is an updated and smaller, and therefore faster, version of the original YOLO architecture. This network is available as a software package in Python^24^.

YOLO networks are trained to predict rectangles enclosing objects in an image, known as bounding boxes. YOLO networks can be trained to differentiate between a wide range of different object classes. In our case, we are only interested in two classes, namely, ‘pipette’ and ‘particle’.

During training, the network aims to minimize a loss function that contains three components: localization, classification and objectness. The localization loss measures the accuracy of the location of the bounding box (center and size) quantified with mean squared error. The classification loss is used to teach the network which class of objects are present in the image, and it uses binary cross-entropy to evaluate how accurately the class is predicted. Lastly, the objectness (or confidence loss) assesses how confident the network is in its predictions. If a box is supposed to contain an object, the loss penalizes low-confidence scores (low objectness); conversely, if the box does not contain any object, it penalizes high-confidence scores (high objectness). For each training image, the network predicts multiple bounding boxes and assigns confidence scores for object presence and class probabilities.

The network was trained using the stochastic gradient descent algorithm on a combination of simulated and manually annotated images (Extended Data Fig. 2). In the manually annotated images, the location and size of particles and pipette were marked by hand using images from previous experiments performed with the same system in order to be as similar as possible to the autonomous experiments. They were of varying size and illumination to cover a wide range of experimental conditions. However, because the exact position was not known and annotation was done manually, it risked introducing noise in the dataset, which is why we also included simulated images in the training set. Because our dataset of manually annotated images is comparatively small (circa 1,000 images), we trained for only 100 epochs to avoid overfitting.

The network consistently predicts the relative focal position of the particles. The GUI allows for demonstrating this by printing the prediction directly on the screen as shown in Supplementary Video 1 in which a particle in the pipette is moved in and out of focus.

### Convolutional network to predict focus position

To estimate the focal position of the particles, we use PyTorch^52^ to train a convolutional network that predicts the *z*-positions of the particles in the images. By using simulated data, we can circumvent the challenges associated with creating an experimental dataset, something which would require accurately measuring the focal position of a very large number of particles^22,25^. The simulated images are made to be similar to the experimental images, and by including noise they are made more challenging, as shown by the examples in Extended Data Fig. 3. The network is a convolutional neural network with two sets of convolutional layers, each followed by a maximum pooling layer. A dense layer on top of this handles the final prediction and training uses mean square error as the loss function. Even though all the training data are simulated, the network also predicts the position of real particles accurately, as illustrated in Extended Data Fig. 4 and Supplementary Video 1.

### U-Net model for accurate particle tracking

For accurate tracking of particles that are very close to one another, which is the case in the electrostatic experiments, we use a U-Net^53^. U-Nets are widely used in biomedical image analysis for segmentation tasks. In a typical segmentation setting, the network outputs a binary image that is 1 wherever an object of interest appears and 0 otherwise. For multiple classes, the output becomes a stack of images, one for each class. To obtain the positions of individual particles from the U-Net predictions, we apply a threshold to the output and then compute the centers of the resulting connected regions. This postprocessing step yields the coordinates of the particles of interest.

The U-Net architecture gets its name from its characteristic ‘U’ shape: an initial series of downsampling layers progressively reduces the spatial resolution of the input while capturing large-scale features, followed by a corresponding set of upsampling layers that restore the original resolution. Residual (skip) connections bridge matching downsampling and upsampling layers, allowing the network to preserve fine details. Our network has five sets of convolutional downsampling layers with 64, 128, 256, 512 and 1,024 filters in the respective layers and the upsampling path is the same but in reversed order (with 1,024, 512, 256, 128 and 64 filters).

The training data consist solely of simulated images^22,25^ as in this case we need to know the true particle position for accuracy. In the simulated images, the number of particles is large so that they often overlap (Extended Data Fig. 7). Furthermore, the size of the particles is tuned to resemble that of the experiment. Very small particles are used in the background of the images to simulate structured noise. This was found to eliminate the risk of the network treating the pipette as a particle.

Despite its higher accuracy, there are two reasons not to use the U-Net in the real-time automation. First, it is much more computationally demanding than YOLO, which would increase the processing time. Reducing the size of the U-Net would help, but this would also reduce its accuracy. Second, to achieve reliable segmentation of the pipette, we would need to include manually annotated experimental data. Consequently, the accuracy would likely not exceed that of YOLO, except when particles are close to overlapping or when there are many particles in view, a situation in which YOLO may struggle, although this was not the case in our experiments.

### Experimental details

All experiments were performed in the same system using the same type of microfluidics. However, each different experiment requires specific preparation of the sample and analysis of data, as outlined below.

#### DNA fragment preparation

The single-molecule force spectroscopy experiments were performed on fragments of *λ*-phage DNA, produced by cutting the full DNA into three different strands and labeling these. The reagents needed for the procedure are listed in Table 1.

**Table 1:** List of reagents used in the *λ*-phage DNA preparation

Description	Company	Material number
Digoxigenin-11-dUTP, 25 nmol	Roche	11093088910
Deoxynucleotide solution set	NEB	N0446S
EagI restriction enzyme	NEB	R3505S
*λ*-DNA, 250ug	NEB	N3011S
Klenow fragment (${3}^{{\prime} }- > {5}^{{\prime} }$ exo-)	NEB	M0212S
Quick ligation kit, 30 reactions	NEB	M2200S
Taq DNA polymerase	NEB	M0273S
Antarctic phosphatase	NEB	M0289S
Biotin-14-dCTP, 50 nmol	Invitrogen	19518-018
PCR primers (see below)	IDT	-

Cutting *λ*-phage DNA with *EagI* yields three fragments: F1 = 19.9 kb, F2 = 16.7 kb and F3 = 11.8 kb.

Biotin end-labeling was done by letting Klenow fragment polymerase fill in the COS sites of *λ*-DNA with biotin-dCTP, which gives up to 6(F1) or 4(F3) biotins at the ends. The F2 fragment did not get biotinylated as we did the Klenow fragment step before cutting with *EagI*.

Digoxigenin modification was done by PCR over an *EagI* cut site on *λ*-DNA. Here, dig-dUTP was incorporated during the PCR reaction. We chose to amplify the region around the cut site at 19944, starting at 224 bases upstream and 266 bases downstream.

Forward primer: 5’-GAAAGCCAGA CGTAACAGCA

Backward primer: 5’-GGCACTTTTT TCCGCTTCAG

Twenty cycles of PCR yielded a 490-base-pair fragment. Digestion with *EagI* gave back two fragments of 224 bases and 266 bases, each containing digoxigenin, and each with a cut overhang (5’-GGCCG) complimentary to the cut ends of *λ* fragments F1 and F3. Those digoxigenin handles were then 5′ dephosphorylated with phosphatase to create an engineered nick in the final dsDNA. Such a nick allows the molecule to change its twist (unwind) during the overstretch transition. Finally, the biotinylated *λ*-DNA fragments and the digoxigenin handles were joined together using an NEB Quick Ligation Kit. Long-term storage of the product (>17 years) has been successful in a buffer of 100 mM EDTA pH 8 and 50% glycerol held in a −20 °C freezer. Free samples are available from S.B.S. (via steveatalice@gmail.com). Further description of the process and its development can be found in the ‘Documents’ section of the TweezersLab website^54^.

#### DNA particle preparation

Polystyrene particles coated with streptavidin were purchased from Spherotech (SVP-20-5; 2.0 mm to 2.9 mm in diameter). Anti-digoxigenin particles were prepared from Protein-G-coated polystyrene particles (Spherotech, PGP-30-5; 3.0 mm to 3.4 mm in diameter).

A total of 1.0 ml of Protein-G-coated particles was washed twice with linking buffer (100 mM Na_2_HPO_4_, 100 mM NaCl, pH 8.5) and incubated with 120 μl of anti-digoxigenin polyclonal antibodies (Roche, 11333089001) and 60 μl of cross-linker dimethyl pimelimidate (Thermo Fisher, 21666). After incubation, the anti-digoxigenin-coated particles were washed twice with PBS buffer (140 mM NaCl, 2.7 mM KCl, 61 mM K_2_HPO_4_, 39 mM KH_2_PO_4_, 0.02% NaN_3_, pH 7.0) and resuspended in 1 ml of PBS buffer. The dsDNA fragments were incubated with digoxigenin-coated particles for 20 min at room temperature and diluted in 2.0 ml of experimental buffer. The streptavidin-coated particles were washed twice with PBS buffer pH 7.4 (140 mM NaCl, 2,7 mM KCl, 80.2 mM K_2_HPO_4_, 20 mM KH_2_PO_4_, 0.02% sodium azide) and diluted in experimental buffer before use.

#### DNA pulling experiment

The experiments presented are performed on the *λ*-DNA F3 fragments (11.8 kb), which has a contour length of 4.0 μm. The two ends of this fragment are labeled with digoxigenin and biotin respectively, which enables them to bind specifically to anti-digoxigenin-coated and streptavidin-coated particles. The anti-digoxigenin particles are around 3.4 μm in diameter and the streptavidin particles are around 2.2 μm, making it possible to visually distinguish the particles. Importantly, only one strand at each DNA end is anchored, resulting in a lower overstretching force than would occur if both strands were immobilized^55^. Also, the F1 (19.9 kb) fragment is labeled in the preparation and approximately 10% of the attached molecules which attach during experiments are F1. The experiments were performed in a high-salt buffer (1 M NaCl, 1 mM EDTA, 10 mM Tris-HCl, components purchased from Sigma-Aldrich). Before the experiment, the DNA is attached to the anti-digoxigenin-coated particles as described above. During the experiment the biotin end will attach to the streptavidin-coated particle held in the micropipette.

The elastic behavior of DNA can be modeled using the WLC model and, in particular, the extensible WLC model works well until the molecule starts to overstretch^56^. The extensible WLC model can be approximated according to equation (3):

3 $$F(x)=\frac{{k}_{{\rm{b}}}T}{{L}_{{\rm{p}}}}\left[\left(\frac{1}{4{(1-x/{L}_{0}+F/{K}_{0})}^{2}}-\frac{1}{4}+\frac{x}{{L}_{0}}-\frac{F}{{K}_{0}}\right.\right]$$

where *k*_b_ is the Boltzmann constant, *x* is the extension of the molecule, *L*_p_ is the persistence length, *T* is the temperature, *K*_0_ is the stretch modulus and *L*_0_ is the length of the molecule. For the curve in Fig. 4, we use a persistence length of 43 nm and a stretch modulus of 1,200 pN, typical values for dsDNA^10^. We solve equation (3) numerically to find the force for a given extension using Python (version 3.13) and the SciPy (version 1.16.1) solver newton.

To determine the particle position, and thus the molecular extension, we use real-time tracking. However, since YOLO is noisier and slower than the PSDs, we use YOLO only to estimate the optical trap stiffness. The stiffness of the optical trap depends on the size of the particles (which varies from particle to particle) and is required for the force–extension analysis. Specifically, we compare the force–extension signal when using the particle position (obtained from YOLO) to the force–extension signal when using the trap position (obtained from the PSDs). The difference between these two signals reflects the displacement of the particle relative to the center of the trap, which is caused by the molecule pulling on the particle. This displacement is linear with the applied force in the force range of the experiment. The linearity of the trap up to around 80 pN was verified separately by trapping particles of similar size and gradually increasing the flow while monitoring their displacement. By fitting the force to the difference between the trap position and the position of the particle, we can determine the stiffness of the trap, without having to perform additional calibration measurements on each particle before each experiment, thus obtaining the molecular extension from the PSD signals. The fitting was performed on the stretching part of the curves in the force range between 14 pN and 55 pN because the sharp increase in the force in this range provides clear cutoffs. This was done using Python (3.13.5) script and specifically the NumPy function polyfit.

### Red blood cell preparation

This study was performed in accordance with the Declaration of Helsinki and was approved by the Work Environment Committee at the Department of Physics at the University of Gothenburg, which identified no further need for ethical evaluation. Written informed consent was also obtained from the sole healthy participant (one of the authors), before initiating the work, and this was the only person handling the samples minimizing the risk of contamination. Human red blood cells are prepared at room temperature immediately before each experiment by finger-pricking using a sterile single-use needle. Approximately 5 μl of blood is extracted and diluted in 2 ml of buffer solution consisting of PBS-10× concentrated buffer (Dulbecco’s PBS 10×, X0520-500) diluted by a factor of 25 in Milli-Q water to which 10 mM of glucose is added. This creates an environment of low osmotic pressure, which makes the cells inflate, becoming almost spherical. During the experiments, the two objectives are placed around 3 μm closer than during normal trapping. There are two main reasons for this small displacement of the objectives: having the two foci of the counterpropagating lasers in different positions reduces heating, while also slightly increasing the stretching. Samples were disposed of immediately after the experiments finished, and the waste treated as potentially contagious and handled using the host universities standard procedures.

### Electrostatic repulsion measurements

The two particles used in the electrostatic experiments are of the same type. They are polystyrene particles with a mean diameter of 4.24 μm ± 0.1 μm (MicroParticles PS-R 4.2, PS/Q-R-B1198).

Electrostatic interactions between colloidal particles can be described by the DLVO theory^57,58^. DLVO theory provides a framework for understanding the interactions at play between the particles, including the van der Waals attraction, the electrostatic repulsion and the screening of the repulsion by dissolved ions. For distances longer than a few nanometers, the interaction is dominated by the electrostatic repulsion. The electrostatic force between two charged particles can be described by equation (4):

4 $$F(D)=\frac{{(eZ)}^{2}}{4\pi {\varepsilon }_{0}{\varepsilon }_{r}}\left(\frac{(1+\kappa (D+2R))\exp (-\kappa D)}{{(1+\kappa R)}^{2}{(D+2R)}^{2}}\right)$$

where *e* is the elementary charge, *Z* is the number of charge groups per particle, *κ* is the Debye length, *D* is the surface-to-surface distance between the particles and *R* is the particles radius^39^. *ε*_0_ and *ε*_*r*_ are the permeability of free space and the relative permeability, respectively. For sufficiently high salinity, the electrostatic repulsion becomes small enough that it can be easily overcome by thermal fluctuations, which enables the particles to come into contact. We exploit this to find the particle radii by performing a final measurement in which the salinity is so high that the particles are brought into contact. This is how we determined a radii of 2.11 μm on average for our particle pair, which is very close to the value of 2.12 μm specified by the manufacturer. From the fit shown in Fig. 6, the number of charge groups was found to be *Z* ≈ 480,000. We performed the fit only on the 0.1 mM dataset because it has a smaller relative error in the distance measurement than the 1 mM dataset, and a smaller relative error in *κ* (due to uncertainty in the salt concentration of our milli-Q water) than the measurement in water without added salt. The fit was performed using Python (version 3.13) and the least_squares function of the SciPy package (version 1.16.1).

### Reporting summary

Further information on research design is available in the Nature Portfolio Reporting Summary linked to this article.

## Online content

Any methods, additional references, Nature Portfolio reporting summaries, source data, extended data, supplementary information, acknowledgements, peer review information; details of author contributions and competing interests; and statements of data and code availability are available at 10.1038/s41592-026-03129-3.

## Supplementary information

Reporting Summary

Peer Review File

Supplementary Video 1 The video is a screen recording of the camera view in the user interface with real-time tracking visualization turned on. The video shows where the program detects the pipette and the particle. This is done while simultaneously moving the pipette with the particle in and out of focus, estimating the particle focal position.

Supplementary Video 2 The video shows the autonomous particle characterization being performed. The video starts with the instrument moving to a capillary to trap a particle. The first particle trapped is one of the target particles, which is determined by the autonomous algorithm based on it being larger than a certain threshold. Since it is one of the target particles, the hydrodynamic radius is measured by moving it between two fixed positions in the sample while recording the forces and motor movement. Once the measurement is finished, the microfluidic pump connected to the central chamber is turned on creating a flow, which removes the trapped particle. Next, another particle is trapped. By chance this is a small particle, which should not be characterized; therefore, it is immediately removed, again using the pump connected to the central chamber. Next, a situation in which multiple particles are trapped is shown. In this case the software detects that the focal position is offset compared to what is expected from a single particle and, therefore, the particles are removed even though the profile size matches that of the target particles. Lastly, several experiments are displayed at a high speed with a timer added to illustrate how the system can run for extended times characterizing dozens of particles. The recording is performed using the interface itself rather than recording the screen because this gives higher video quality than a screen recording.

Supplementary Video 3 The video illustrates the autonomous DNA pulling. First, a recording of a single autonomous pulling is shown starting with an empty chamber, without particles in either trap or pipette. This part of the video is commented. It is a recorded camera feed showing all the different steps of the autonomous pulling process. It starts by checking the pipette. Then the streptavidin particle is trapped and positioned in the pipette. This is followed by the trapping of a particle with DNA, which is then attached to the particle in the pipette where the experiment measurement is performed. After this first pulling, the video shows the full GUI while the program performs a large number of pullings autonomously. This part of the video is shown at increased speed (to limit the duration and size of the video) and also the real-time plotting is shown. At the end, a recording from a 10-h continuous experiment is shown. This is shown at 600 times the normal speed (10 min shown in 1 s) to show long-term operation.

Supplementary Video 4 The video shows how the red blood cell experiments are performed by the instrument. The video is a screen recording of the GUI and also includes a timer. After a cell has been trapped, initially at low power, the cell profile is recorded in a video. Then, the trapping power is briefly increased and the cell profile at higher power is recorded in another video. Thereafter, the trapping power is reduced and the process repeated four more times at increasingly high powers. Thereafter, the cell is removed by a flow. Since there are cells dispersed in the medium, the flow is also used to bring new cells into view, which are trapped and measured. If by chance there are no cells in view after flowing new medium, then the flow is briefly turned on again. The video speed is increased to show more experiments.

Supplementary Video 5 The video shows how electrostatic repulsion can be measured autonomously. When the video starts, there is nothing in either trap or pipette. The program first focuses the pipette and checks its content. Next, it moves to the capillary, turns on the microfluidic pump, and traps a particle. The trapped particle is then placed in the pipette. After confirming that the particle has been successfully transferred to the pipette, a second particle is trapped and brought to the pipette. The particle in the trap is aligned to the particle in the pipette. The trapped particle is pushed toward the particle in the pipette by moving the trap to find appropriate limits for the measurement protocol. Next, the protocol and recording of data (both force and video) are started. At this stage, the program zooms in on the two particles to limit the size of the videos. Once the measurement is completed, a strong flow removes both particles, resetting the experiment and preparing the system for another measurement.

## Data Availability

The data presented in the figures are available in figshare at 10.6084/m9.figshare.31281967 (ref. ^51^). Due to the size of the raw data files and the datasets used for training the neural networks, these are available from the authors upon reasonable request. The repository also contains an unedited, compressed video of a 10-h experimental run recorded from the instrument.

## Code availability

The source code used for the SmartTrap system is available on Zenodo. This and future software releases are available from our GitHub and Zenodo repositories^42,43^. We highlight that the various artificial networks presented are available from Zenodo, and on figshare via 10.6084/m9.figshare.31281967 (ref. ^51^). The networks were run using Python version 3.13, PyTorch version 2.8.0+cu126, ultralytics version 8.3.196 and yolov5 version 7.0.14.

## References

[1] Lu, C. et al. Towards end-to-end automation of AI research. *Nature* **651**, 914–919 (2026). 41882133 10.1038/s41586-026-10265-5 PMC13017497

[2] Jumper, J. et al. Highly accurate protein structure prediction with AlphaFold. *Nature* **596**, 583–589 (2021). 34265844 10.1038/s41586-021-03819-2 PMC8371605

[3] Volpe, G. et al. *Deep Learning Crash Course* (No Starch Press, 2026).

[4] Holland, I. & Davies, J. A. Automation in the life science research laboratory. *Front. Bioeng. Biotechnol.* **8**, 571777 (2020). 33282848 10.3389/fbioe.2020.571777 PMC7691657

[5] Ashkin, A. Acceleration and trapping of particles by radiation pressure. *Phys. Rev. Lett.* **24**, 156 (1970).

[6] Ashkin, A., Dziedzic, J. M., Bjorkholm, J. E. & Chu, S. Observation of a single-beam gradient force optical trap for dielectric particles. *Opt. Lett.* **11**, 288–290 (1986). 19730608 10.1364/ol.11.000288

[7] Volpe, G. et al. Roadmap for optical tweezers. *J. Phys. Photonics* **5**, 022501 (2023).

[8] Magazzù, A. & Marcuello, C. Investigation of soft matter nanomechanics by atomic force microscopy and optical tweezers: a comprehensive review. *Nanomaterials* **13**, 963 (2023). 36985857 10.3390/nano13060963 PMC10053849

[9] Turlier, H. et al. Equilibrium physics breakdown reveals the active nature of red blood cell flickering. *Nat. Phys.* **12**, 513–519 (2016).

[10] Bustamante, C. J., Chemla, Y. R., Liu, S. & Wang, M. D. Optical tweezers in single-molecule biophysics. *Nat. Rev. Methods Primers* **1**, 25 (2021). 34849486 10.1038/s43586-021-00021-6 PMC8629167

[11] Liu, S., Chistol, G. & Bustamante, C. Mechanical operation and intersubunit coordination of ring-shaped molecular motors: insights from single-molecule studies. *Biophys. J.* **106**, 1844–1858 (2014). 24806916 10.1016/j.bpj.2014.03.029 PMC4017299

[12] Zhang, H. & Liu, K. -K. Optical tweezers for single cells. *J. R. Soc. Interface* **5**, 671–690 (2008). 18381254 10.1098/rsif.2008.0052 PMC2408388

[13] Grier, D. G. Optical tweezers in colloid and interface science. *Curr. Opin. Colloid Interface Sci.* **2**, 264–270 (1997).

[14] Hertlein, C., Helden, L., Gambassi, A., Dietrich, S. & Bechinger, C. Direct measurement of critical Casimir forces. *Nature* **451**, 172–175 (2008). 18185584 10.1038/nature06443

[15] Crocker, J. C., Matteo, J. A., Dinsmore, A. D. & Yodh, A. G. Entropic attraction and repulsion in binary colloids probed with a line optical tweezer. *Phys. Rev. Lett.* **82**, 4352 (1999).

[16] Rissone, P., Rico-Pasto, M., Smith, S. B. & Ritort, F. Dna calorimetric force spectroscopy at single base pair resolution. *Nat. Commun.* **16**, 2688 (2025). 40108107 10.1038/s41467-025-57340-5 PMC11923082

[17] Cichos, F., Gustavsson, K., Mehlig, B. & Volpe, G. Machine learning for active matter. *Nat. Mach. Intell.* **2**, 94–103 (2020).

[18] Ciarlo, A. et al. Deep learning for optical tweezers. *Nanophotonics* **13**, 3017–3035 (2024). 39634937 10.1515/nanoph-2024-0013 PMC11502085

[19] Melzer, J. E. & McLeod, E. Assembly of multicomponent structures from hundreds of micron-scale building blocks using optical tweezers. *Microsyst. Nanoeng.* **7**, 45 (2021). 34567758 10.1038/s41378-021-00272-z PMC8433220

[20] Teixeira, J. et al. Autonomous and intelligent optical tweezers for improving the reliability and throughput of single particle analysis. *Meas. Sci. Technol.* **35**, 025208 (2023).

[21] Smith, S. B., Cui, Y. & Bustamante, C. Optical-trap force transducer that operates by direct measurement of light momentum. *Methods Enzymol.* 134–162 (2003). 10.1016/s0076-6879(03)61009-8 12624910

[22] Midtvedt, B. et al. Quantitative digital microscopy with deep learning. *Appl. Phys. Rev*. **8**, 011310 (2021).

[23] Redmon, J., Divvala, S., Girshick, R. & Farhadi, A. You Only Look Once: unified, real-time object detection. In *Proc. IEEE Conference on Computer Vision and Pattern Recognition* 779–788 (IEEE, 2016).

[24] Jocher, G. Ultralytics yolov5. *GitHub* https://github.com/ultralytics/yolov5 (2020).

[25] Team, D. Deeptrack 2.0. *GitHub* https://github.com/deepTrackAI/deeptrack2 (2025).

[26] Woods, D. L., Wyma, J. M., Yund, E. W., Herron, T. J. & Reed, B. Factors influencing the latency of simple reaction time. *Front. Hum. Neurosci.* **9**, 131 (2015). 25859198 10.3389/fnhum.2015.00131 PMC4374455

[27] Heller, I., Hoekstra, T. P., King, G. A., Peterman, E. J. & Wuite, G. J. Optical tweezers analysis of DNA–protein complexes. *Chem. Rev.* **114**, 3087–3119 (2014). 24443844 10.1021/cr4003006

[28] Neuman, K. C. & Nagy, A. Single-molecule force spectroscopy: optical tweezers, magnetic tweezers and atomic force microscopy. *Nat. Methods* **5**, 491–505 (2008). 18511917 10.1038/nmeth.1218 PMC3397402

[29] Dame, R. T., Noom, M. C. & Wuite, G. J. Bacterial chromatin organization by h-ns protein unravelled using dual DNA manipulation. *Nature* **444**, 387–390 (2006). 17108966 10.1038/nature05283

[30] Bustamante, C., Bryant, Z. & Smith, S. B. Ten years of tension: single-molecule DNA mechanics. *Nature* **421**, 423–427 (2003). 12540915 10.1038/nature01405

[31] Smith, S. B., Cui, Y. & Bustamante, C. Overstretching b-DNA: the elastic response of individual double-stranded and single-stranded DNA molecules. *Science* **271**, 795–799 (1996). 8628994 10.1126/science.271.5250.795

[32] Bustamante, C., Marko, J. F., Siggia, E. D. & Smith, S. Entropic elasticity of *λ*-phage DNA. *Science* **265**, 1599–1600 (1994). 8079175 10.1126/science.8079175

[33] Omori, T. et al. Tension of red blood cell membrane in simple shear flow. *Phys. Rev. E* **86**, 056321 (2012). 10.1103/PhysRevE.86.056321 23214889

[34] Diez-Silva, M., Dao, M., Han, J., Lim, C. -T. & Suresh, S. Shape and biomechanical characteristics of human red blood cells in health and disease. *MRS Bull.* **35**, 382–388 (2010). 21151848 10.1557/mrs2010.571 PMC2998922

[35] Guo, Q. et al. Microfluidic analysis of red blood cell deformability. *J. Biomech.* **47**, 1767–1776 (2014). 24767871 10.1016/j.jbiomech.2014.03.038

[36] Guck, J. et al. The optical stretcher: a novel laser tool to micromanipulate cells. *Biophys. J.* **81**, 767–784 (2001). 11463624 10.1016/S0006-3495(01)75740-2 PMC1301552

[37] Cosgrove, T. *Colloid Science: Principles, Methods and Applications* (John Wiley & Sons, 2010).

[38] Adamczyk, Z. & Warszyński, P. Role of electrostatic interactions in particle adsorption. *Adv. Colloid Interface Sci.* **63**, 41–149 (1996).

[39] Gutsche, C., Keyser, U., Kegler, K., Kremer, F. & Linse, P. Forces between single pairs of charged colloids in aqueous salt solutions. *Phys. Rev. E* **76**, 031403 (2007). 10.1103/PhysRevE.76.031403 17930243

[40] Baumgartl, J. & Bechinger, C. On the limits of digital video microscopy. *Europhys. Lett.* **71**, 487 (2005).

[41] Huguet, J. M. et al. Single-molecule derivation of salt dependent base-pair free energies in DNA. *Proc. Natl Acad. Sci. USA* **107**, 15431–15436 (2010). 20716688 10.1073/pnas.1001454107 PMC2932562

[42] Selin, M. et al. SmartTrap. *GitHub* https://github.com/softmatterlab/SmartTrap (2026).

[43] Selin, M. et al. SmartTrap. *Zenodo*10.5281/zenodo.18507341 (2026).

[44] Smith, S. B. & Rivetti, C. TweezersLAB. http://tweezerslab.unipr.it/cgi-bin/home.pl (2009).

[45] Bustamante, C. J. & Smith, S. B. Light-force sensor and method for measuring axial optical-trap forces from changes in light momentum along an optic axis. US Patent Number: 7133132 B2 https://patents.google.com/patent/US7133132B2/en (2006).

[46] Bustamante, C. J. & Smith, S. B. Optical beam translation device and method utilizing a pivoting optical fiber. US Patent Number: 7274451 B2 https://patents.google.com/patent/US7274451B2/en (2007).

[47] Stokes, G. G. On the effect of the internal friction of fluids on the motion of pendulums. *Trans. Camb. Philos. Soc.* **9**, 8–106 (1851).

[48] Smith, S. B., Finzi, L. & Bustamante, C. Direct mechanical measurements of the elasticity of single DNA molecules by using magnetic beads. *Science* **258**, 1122–1126 (1992). 1439819 10.1126/science.1439819

[49] Linstrom, P. & Mallard, W. E. NIST Chemistry WebBook, NIST Standard Reference Database Number 69. https://webbook.nist.gov/ (2025).

[50] Goodfellow, I., Bengio, Y. & Courville, A. *Deep Learning* (MIT Press, 2016).

[51] Selin, M. et al. Supporting data SmartTrap (NMETH-A61532A). *figshare*10.6084/m9.figshare.31281967.v1 (2026).

[52] Paszke, A. et al. PyTorch: an imperative style, high-performance deep learning library. Preprint at https://arxiv.org/abs/1912.01703 (2019).

[53] Ronneberger, O., Fischer, P. & Brox, T. U-net: convolutional networks for biomedical image segmentation. In *Medical Image Computing and Computer-assisted Intervention–MICCAI 2015* 234–241 (Springer, 2015).

[54] Bosaeus, N. & Smith, S. B. How to make 1/3 lambda DNA for the minitweezers. http://tweezerslab.unipr.it/documents/att/19cc.file.01037.pdf (2009).

[55] van Mameren, J. et al. Unraveling the structure of DNA during overstretching by using multicolor, single-molecule fluorescence imaging. *Proc. Natl Acad. Sci. USA* **106**, 18231–18236 (2009). 19841258 10.1073/pnas.0904322106 PMC2775282

[56] Wang, M. D., Yin, H., Landick, R., Gelles, J. & Block, S. M. Stretching DNA with optical tweezers. *Biophys. J.* **72**, 1335–1346 (1997). 9138579 10.1016/S0006-3495(97)78780-0 PMC1184516

[57] Derjaguin, B. & Landau, L. D. Theory of the stability of strongly charged lyophobic sol and of the adhesion of strongly charged particles in solutions of electrolytes. *Acta Physicochim. URSS* **14**, 633–662 (1941).

[58] Verwey, E. J. W. Theory of the stability of lyophobic colloids. *J. Phys. Chem.* **51**, 631–636 (1947). 10.1021/j150453a001 20238663

