# Supplementary material for: SmartTrap: automated precision experiments with optical tweezers
Source: Nat Methods. 2026 Jun 18;23(7):1368–78. doi: 10.1038/s41592-026-03129-3 (PMC13346115; doi:10.1038/s41592-026-03129-3)
Supplement: Supplementary file 1 — Reporting Summary [file 41592_2026_3129_MOESM1_ESM.pdf]

Corresponding author(s): Giovanni Volpe

Last updated by author(s): Feb 8, 2026

Reporting Summary

Nature Portfolio wishes to improve the reproducibility of the work that we publish. This form provides structure for consistency and transparency in reporting. For further information on Nature Portfolio policies, see our Editorial Policies and the Editorial Policy Checklist.

Statistics

For all statistical analyses, confirm that the following items are present in the figure legend, table legend, main text, or Methods section.

n/a	Confirmed	
☐	☒	The exact sample size (n) for each experimental group/condition, given as a discrete number and unit of measurement
☐	☒	A statement on whether measurements were taken from distinct samples or whether the same sample was measured repeatedly
☒	☐	The statistical test(s) used AND whether they are one- or two-sided<br>*Only common tests should be described solely by name; describe more complex techniques in the Methods section.*
☒	☐	A description of all covariates tested
☒	☐	A description of any assumptions or corrections, such as tests of normality and adjustment for multiple comparisons
☐	☒	A full description of the statistical parameters including central tendency (e.g. means) or other basic estimates (e.g. regression coefficient) AND variation (e.g. standard deviation) or associated estimates of uncertainty (e.g. confidence intervals)
☒	☐	For null hypothesis testing, the test statistic (e.g. F, t, r) with confidence intervals, effect sizes, degrees of freedom and P value noted<br>*Give P values as exact values whenever suitable.*
☒	☐	For Bayesian analysis, information on the choice of priors and Markov chain Monte Carlo settings
☒	☐	For hierarchical and complex designs, identification of the appropriate level for tests and full reporting of outcomes
☒	☐	Estimates of effect sizes (e.g. Cohen's d, Pearson's r), indicating how they were calculated

Our web collection on statistics for biologists contains articles on many of the points above.

Software and code

Policy information about availability of computer code

Data collection	The source code used for the SmartTrap system is available on Zenodo https://doi.org/10.5281/zenodo.18507341, this and future software releases are available from our GitHub https://github.com/softmatterlab/SmartTrap and Zenodo repositories. Data acquisition and experimental control were performed using custom open-source SmartTrap software (v1.0) implemented in Python (v3.13.5). The software controlled optical trapping, micropipette positioning, imaging, and signal acquisition, and enabled real-time feedback during experiments. Real-time image-based object detection during data acquisition was performed using YOLOv5 (v5.0) convolutional neural networks, executed using PyTorch (v2) with CUDA (v12) support on an NVIDIA RTX 3090 GPU.
Data analysis	Data analysis was performed using custom Python scripts (Python v3.13.5) within the SmartTrap framework. Image analysis employed YOLOv5 (v5.0) and custom convolutional neural networks, including U-Net architectures, trained using synthetic datasets generated with DeepTrack2 (v2). Numerical data analysis and curve fitting were performed using NumPy (v2) and SciPy (v1.16). The networks were run using Python Version 3.13, pytorch version 2.8.0+cu126, ultralytics version 8.3.196 and yolov5 version 7.0.14.

For manuscripts utilizing custom algorithms or software that are central to the research but not yet described in published literature, software must be made available to editors and reviewers. We strongly encourage code deposition in a community repository (e.g. GitHub). See the Nature Portfolio guidelines for submitting code & software for further information.

Data

Policy information about availability of data

All manuscripts must include a data availability statement. This statement should provide the following information, where applicable:

- Accession codes, unique identifiers, or web links for publicly available datasets
- A description of any restrictions on data availability
- For clinical datasets or third party data, please ensure that the statement adheres to our policy

The data presented in the figures are available in FigShare https://figshare.com/s/9e6621b85b1287ce8757. Due to the size of the raw data files, these are available from the authors upon reasonable request. The repository also contains an unedited video of a 10 hour experimental run recorded from the instrument which has been compressed to limit the file size.

Research involving human participants, their data, or biological material

Policy information about studies with human participants or human data. See also policy information about sex, gender (identity/presentation), and sexual orientation and race, ethnicity and racism.

Reporting on sex and gender	not relevant for this study
Reporting on race, ethnicity, or other socially relevant groupings	not relevant for this study
Population characteristics	not relevant for this study
Recruitment	not relevant for this study
Ethics oversight	not relevant for this study

Note that full information on the approval of the study protocol must also be provided in the manuscript.

Field-specific reporting

Please select the one below that is the best fit for your research. If you are not sure, read the appropriate sections before making your selection.

☒ Life sciences ☐ Behavioural & social sciences ☐ Ecological, evolutionary & environmental sciences

For a reference copy of the document with all sections, see nature.com/documents/nr-reporting-summary-flat.pdf

Life sciences study design

All studies must disclose on these points even when the disclosure is negative.

Sample size	Sample sizes reflect the typical number of measurements which can be performed on a single sample (e.g. batch of particles with DNA attached) before depletion. This choice was made to showcase that the system can operate for a long time independently and to provide an estimate of how many measurements one can expect per hour of operation.
Data exclusions	The autonomous system saves all measurements, also when technical issues occur during measurement (e.g., unintended trapping of a second particle or molecular tether breakage). Such events lead the system to terminate the current measurement and initiate a new one, resulting in certain incomplete datasets that require exclusion prior to analysis. The specific criteria for exclusion varied between experiments:<br>DNA pulling experiments: Measurements were excluded if the force exceeded 70 pN without observable overstretching or if the molecular tether prematurely ruptured. Also, as stated in the methods section, we only show data from the f3 DNA fragments.<br>Particle characterization experiments: Data from measurements where particles were unintentionally dropped before completion were excluded due to incompleteness.<br>Red blood cell experiments: Only cells that completed the entire measurement cycle were included; data from prematurely lost cells were excluded.<br>Repulsion experiments: No datasets were excluded, although analyses focused exclusively on the experimentally relevant timeframes.
Replication	The reproducibility of the results is shown by the autonomous system's ability to independently perform multiple measurements with near identical results but on different molecules, particles or cells.
Randomization	Explicit randomization was not applicable, as the autonomous system selects samples (particles, cells, and DNA molecules) without human intervention, minimizing selection bias.
Blinding	Blinding is not applicable. All measurements were autonomously performed by the instrument, generating quantifiable data without room for subjective interpretation.

Reporting for specific materials, systems and methods

We require information from authors about some types of materials, experimental systems and methods used in many studies. Here, indicate whether each material, system or method listed is relevant to your study. If you are not sure if a list item applies to your research, read the appropriate section before selecting a response.

Materials & experimental systems

n/a	Involved in the study
☒	☐ Antibodies
☒	☐ Eukaryotic cell lines
☒	☐ Palaeontology and archaeology
☒	☐ Animals and other organisms
☒	☐ Clinical data
☒	☐ Dual use research of concern
☒	☐ Plants

Methods

n/a	Involved in the study
☒	☐ ChIP-seq
☒	☐ Flow cytometry
☒	☐ MRI-based neuroimaging

Plants

Seed stocks	not relevant for this study
Novel plant genotypes	not relevant for this study
Authentication	not relevant for this study
