# Supplementary material for: SmartTrap: automated precision experiments with optical tweezers
Source: Nat Methods. 2026 Jun 18;23(7):1368–78. doi: 10.1038/s41592-026-03129-3 (PMC13346115; doi:10.1038/s41592-026-03129-3)
Supplement: Supplementary file 2 — Peer Review File [file 41592_2026_3129_MOESM2_ESM.pdf]

nature portfolio

Peer Review File

SmartTrap: Automated Precision Experiments with Optical Tweezers

Corresponding Author: Professor Giovanni Volpe

This file contains all editorial decision letters in order by version, followed by all author rebuttals in order by version.

Version 0:

Decision Letter:

28th Jul 2025

Dear Giovanni,

Your Article, "SmartTrap: Automated Precision Experiments with Optical Tweezers", has now been seen by three reviewers. As you will see from their comments below, although the reviewers find your work of considerable potential interest, they have raised a number of concerns. We continue to be interested in potentially publishing your manuscript in Nature Methods, but would like to evaluate your response to these concerns before we reach a final decision on publication. We therefore invite you to revise your manuscript to address these concerns.

We ask that you focus your revision on addressing the concerns raise by reviewers 2 and 3 on general applicability of your approach beyond a specific instrument. We also ask that you address issues of data quality and address technical questions. Regarding concerns of novelty raised by reviewer 1, we ask that you clarify that the optical trapping instrument itself is not new, but rather the software tools enabling automated trapping experiments. Please do provide the requested specifications of the instrumentation so the reviewers can assess the data appropriately.

We are committed to providing a fair and constructive peer-review process. Do not hesitate to contact us if there are specific requests from the reviewers that you believe are technically impossible or unlikely to yield a meaningful outcome.

When revising your paper:

* include a point-by-point response to the reviewers and to any editorial suggestions

* please underline/highlight any additions to the text or areas with other significant changes to facilitate review of the revised manuscript

* address the points listed described below to conform to our open science requirements

* ensure it complies with our general format requirements as set out in our guide to authors at www.nature.com/naturemethods

* resubmit all the necessary files by using the link below to access your home page

Link Redacted

Note: This URL links to your confidential account and associated information about manuscripts you may have submitted, or that you are reviewing for us. If you wish to forward this email to co-authors, please delete this link.

We hope to receive your revised paper within two months. If you are substantially delayed, please let us know. In this event, we will still be happy to reconsider your paper at a later date so long as nothing similar has been accepted for publication at Nature Methods or published elsewhere.

OPEN SCIENCE REQUIREMENTS

REPORTING SUMMARY
When revising your manuscript, please update your reporting summary.

Reporting summary: https://www.nature.com/documents/nr-reporting-summary.zip

If your paper includes custom software, we also ask you to complete a supplemental reporting summary.

Software supplement: https://www.nature.com/documents/nr-software-policy.pdf

Please submit these with your revised manuscript. They will be available to reviewers to aid in their evaluation if the paper is re-reviewed. If you have any questions about the checklist, please see http://www.nature.com/authors/policies/availability.html or contact me.

Please note that these forms are dynamic 'smart pdfs' and must therefore be downloaded and completed in Adobe Reader. We will then flatten them for ease of use by the reviewers. If you would like to reference the guidance text as you complete the template, please access these flattened versions at http://www.nature.com/authors/policies/availability.html.

For any revision that includes light microscopy data, we ask our authors to please include a completed light microscopy reporting table [https://www.nature.com/documents/Light_microscopy_reporting_table.xlsx] to ensure the methods are described thoroughly. The table will be available to reviewers and ultimately published should the manuscript be accepted at the journal.

IMAGE INTEGRITY
When submitting the revised version of your manuscript, please pay close attention to our Digital Image Integrity Guidelines and to the following points below:

-- that unprocessed scans are clearly labelled and match the gels and western blots presented in figures.
-- that control panels for gels and western blots are appropriately described as loading on sample processing controls
-- all images in the paper are checked for duplication of panels and for splicing of gel lanes.

Finally, please ensure that you retain unprocessed data and metadata files after publication, ideally archiving data in perpetuity, as these may be requested during the peer review and production process or after publication if any issues arise.

EXTENDED DATA FIGURES
When re-submitting your manuscript, please ensure that any supplementary figures and tables that are crucial to the manuscript's conclusions are converted into Extended Data figures and tables to increase visibility of these data. Extended Data figures and tables are online-only (present in the online PDF and full-text HTML versions of the paper), peer-reviewed display items that provide essential background to the article but are not included in the main article due to space constraints. A maximum of ten Extended Data display items (figures and tables) is permitted.

DATA AVAILABILITY
We strongly encourage you to deposit all new data associated with the paper in a persistent repository where they can be freely and enduringly accessed. We recommend submitting the data to discipline-specific and community-recognized repositories; a list of repositories is provided here: http://www.nature.com/sdata/policies/repositories

All novel DNA and RNA sequencing data, protein sequences, genetic polymorphisms, linked genotype and phenotype data, gene expression data, macromolecular structures, and proteomics data must be deposited in a publicly accessible database, and accession codes and associated hyperlinks must be provided in the "Data Availability" section.

For papers containing bioimaging data, we strongly recommend depositing the data to Bioimage Archive (https://www.ebi.ac.uk/bioimage-archive/). Associated accession codes and hyperlinks should be provided in the "Data Availability" section.

Refer to our data policies here: https://www.nature.com/nature-research/editorial-policies/reporting-standards#availability-of-data

To further increase transparency, we encourage you to provide, in tabular form, the data underlying the graphical representations used in your figures. This is in addition to our data-deposition policy for specific types of experiments and large datasets. For readers, the source data will be made accessible directly from the figure legend. Spreadsheets can be submitted in .xls, .xlsx or .csv formats. Only one (1) file per figure is permitted: thus if there is a multi-paneled figure the source data for each panel should be clearly labeled in the csv/Excel file; alternately the data for a figure can be included in multiple, clearly labeled sheets in an Excel file. File sizes of up to 30 MB are permitted. When submitting source data files with your manuscript

please select the Source Data file type and use the Title field in the File Description tab to indicate which figure the source data pertains to.

Please include a “Data availability” subsection in the Online Methods. This section should inform readers about the availability of the data used to support the conclusions of your study, including accession codes to public repositories, references to source data that may be published alongside the paper, unique identifiers such as URLs to data repository entries, or data set DOIs, and any other statement about data availability. At a minimum, you should include the following statement: “The data that support the findings of this study are available from the corresponding author upon request”, describing which data is available upon request and mentioning any restrictions on availability. If DOIs are provided, please include these in the Reference list (authors, title, publisher (repository name), identifier, year). For more guidance on how to write this section please see: http://www.nature.com/authors/policies/data/data-availability-statements-data-citations.pdf

CODE AVAILABILITY
Please include a “Code Availability” subsection in the Online Methods which details how your custom code is made available. Only in rare cases (where code is not central to the main conclusions of the paper) is the statement “available upon request” allowed (and reasons should be specified).

We request that you deposit code in a DOI-minting repository such as Zenodo, Gigantum or Code Ocean and cite the DOI in the Reference list. We also request that you use code versioning and provide a license.

For more information on our code sharing policy and requirements, please see: https://www.nature.com/nature-research/editorial-policies/reporting-standards#availability-of-computer-code

MATERIALS AVAILABILITY
As a condition of publication in Nature Methods, authors are required to make unique materials promptly available to others without undue qualifications.

Authors reporting new chemical compounds must provide chemical structure, synthesis and characterization details. Authors reporting mutant strains and cell lines are strongly encouraged to use established public repositories.

More details about our materials availability policy can be found at https://www.nature.com/nature-portfolio/editorial-policies/reporting-standards#availability-of-materials

ORCID
Nature Methods is committed to improving transparency in authorship. As part of our efforts in this direction, we are now requesting that all authors identified as ‘corresponding author’ on published papers create and link their Open Researcher and Contributor Identifier (ORCID) with their account on the Manuscript Tracking System (MTS), prior to acceptance. This applies to primary research papers only. ORCID helps the scientific community achieve unambiguous attribution of all scholarly contributions. You can create and link your ORCID from the home page of the MTS by clicking on ‘Modify my Springer Nature account’. For more information please visit please visit www.springernature.com/orcid.

Please do not hesitate to contact me if you have any questions or would like to discuss these revisions further. We look forward to seeing your revised manuscript and thank you for the opportunity to consider your work.

Best regards,
Rita

Rita Strack, Ph.D.
Senior Editor
Nature Methods

Reviewers' Comments:

Reviewer #1 (Remarks to the Author):

This paper presents a custom experimental setup utilizing optical tweezers and associated software. The setup is engineered to automate measurements. For this purpose, it incorporates object detection routines that enable the identification of specific particles and the determination of their three-dimensional positions. Several examples of potential applications and measurement protocols are provided, including the automated measurement of force-extension data for DNA strands. The presented setup constitutes an advanced tool to obtain quantitative information about forces acting on biological samples on the micro and nanoscale.

The Nature Methods journal imposes stringent requirements regarding novelty. However, I encounter difficulties in pinpointing the precise and groundbreaking novel contribution of this work. The manuscript appears to be somewhat unclear on this aspect. The optical setup itself appears to be a reimplementation of an earlier design, but only a schematic figure is provided. In my opinion, the description is insufficiently comprehensive and omits crucial details about the setup. A significantly more detailed technical description would be necessary if this were the primary achievement. Also, the benefits and drawbacks of some design decisions need to be discussed in more detail, e.g. I expect that dual trap optical tweezers would show an improved stability of the relative position compared to the presented design, thus relaxing the requirements on the mechanical stability of the setup. Another issue I see is that the presented setup is highly customized, which makes it difficult to replicate, also given the lack of technical details in this manuscript. This limits the broad interest. The basic capabilities of the presented setup compete e.g. with commercially available optical tweezers setups from Impetux or JPK/Bruker with similar features. In which aspects does the presented setup and methods excel, where does it lag behind?

The demonstration experiments presented, while showcasing the impressive flexibility of photonic force microscopy, appear to lack novelty. The measurements of drag forces, force-extension data of DNA strands, cell deformation in an optical trap, and electrostatic repulsion between particles, although well-executed, do not introduce new methods.

In my assessment, the primary contribution lies in the integration of object detection and position tracking capabilities from camera images into the experiment control system. I commend the software's promise of public availability. This enhancement enables automatic particle capture and monitoring of single particles in optical traps, presenting a valuable feature to the control software. It facilitates the automation of a process traditionally requiring human intervention.
On the other hand, object detection is a prevalent task in computer vision. Notably, the software utilizes a widely available model (Ultralytics YOLO) for this purpose. While this provides robust particle detection, it appears somewhat over-engineered. Simpler standard computer vision algorithms could have achieved the same task with enhanced computational efficiency, particularly for the presented applications where only spherical particles were utilized.
Alternative setups employ microfluidics to deliver particles to the optical trap, eliminating the need for advanced image analysis. The performance of the specialized artificial neural networks employed for 3D position tracking remains unclear and warrants further characterization. How does it compare in performance e.g. to fitting Lorenz-Mie theory to inline holograms, as e.g. demonstrated by David Grier's group.

The authors claim that the full automatization allows for large-scale studies. However, for specific tasks there exist optimized alternative methods that allow throughput orders of magnitudes higher than with single beam optical tweezers, such as microfluidics for measuring cell mechanical properties (see e.g. https://doi.org/10.1016/j.bpj.2015.05.029) or using magnetic or acoustic forces for DNA stretching. This is not properly discussed.

The presentation of the methods requires refinement. The fundamental details of the setup are inadequately described. For instance, the authors refer to "the motors" in multiple instances within the text, yet the essential information about their purpose in translating the sample is concealed within the supplemental information. No information regarding the specific type and performance of these motors is given. Another example is found in the description of the DNA pulling experiment, where it is stated that „The micro-controller provides
feedback at 7 kHz, much faster than the communication from the host computer, which is why
this protocol is run directly on the micro-controller rather than the host offering smoother motion of the lasers.“ However, since feedback from image analysis is also mentioned, it becomes unclear which task precisely is performed by the micro-controller and which by the host computer. Furthermore, the method employed to measure the extension data of Fig. 4 remains unclear.

In addition, I have reviewed the SmartTrap control software, which provides a comprehensive and user-friendly platform for experiment control. However, from a software engineering perspective, its limited modularization and lack of abstraction layers present challenges regarding its reusability for diverse purposes beyond its initial design. For example, the software integrates user interface management, low-level hardware control, and control logic at the same level, which impedes its adaptability. Furthermore, its close coupling to specific hardware restricts its broader applicability.

In my assessment, while the manuscript possesses certain notable features, it falls short of meeting the essential criteria for publication in Nature Methods. Specifically, it lacks the broad interest, novelty, and comprehensive technical description that are crucial for this esteemed journal.

Reviewer #2 (Remarks to the Author):

The authors describe the SmartTrap, an integrated, open-source optical tweezers platform that combines deep learning–based 3D particle tracking, microfluidics, and custom electronics to automate single-cell and single-molecule assays (e.g., such as DNA force-extension measurements and red blood cell deformation). The text and associated videos demonstrate autonomous execution with fast hardware responses and multi-hour continuous operation

To start, I would like to say that I appreciate the motivation behind the manuscript – single-molecule and single-cell optical tweezers measurements could indeed greatly benefit from increased throughput. I also very much appreciate the open-source approach presented here and think that if it is broadly adopted, SmartTrap could indeed push forward the next generation of optical tweezers research. The integration of advanced AI-based tracking with fast feedback loops and custom microfluidics and its successful demonstration using some standard protocols (DNA pulling, red blood cell stretching, measurement of electrostatic interactions between two particles) shows that SmartTrap goes beyond simply being an incremental development in the field.

Taking this into account, I found the described technique to be suitable for publication in Nature Methods, but I do have some points that I would like the authors to address

Major points:
1. The manuscript does not currently include a “Conflict of Interest” statement. I note that one of the co authors, Steven B. Smith, is known to make and sell optical tweezers systems. This commercial tie should be explicitly disclosed according to Nature Methods guidelines, or otherwise commented upon if deemed irrelevant.

2. A key limitation of the SmartTrap is its strict dependence on a specific optical-tweezers configuration. Both the experimental protocols and deep-learning models are calibrated and trained exclusively on this setup. Thus, labs with even slight variations in optics, cameras, microfluidics, or electronics would need substantial retraining and reconfiguration before the platform functions, limiting its immediate generalisability. It would be appropriate to highlight this point, clarifying that while technically impressive, SmartTrap currently addresses a small community of labs and may require significant resources and custom integration to be adopted by others. I would encourage the authors to discuss how much effort (e.g., both in terms of model training and actual experimental setup) would be necessary to adapt the platform to other hardware, or whether the idea is rather that labs purchase their own SmartTraps (this is related to point 1, above).

3. The data shown in Figure S5 and Figure 4 of the DNA stretching seem to show quite some noise/deviation from the WLC and the stretching and refolding curves do not match up perfectly, as I would expect them to, in Figure 4. The data in Figure S5 are very zoomed out so it is difficult to judge the quality of the data well. Can the authors comment on the force and distance resolution of the instrument (in the manuscript) and provide a close-up of the data in S5 in the distance range of 3 – 4.5 m (for my own inspection)? It seems important to mention that many single-molecule experiments performed using the optical tweezers require excellent, repeatable high-resolution data and that knowledge of the limitations of the instrument is important to assess how broadly it can be applied for studies of DNA-protein interactions, RNA and protein folding. The manuscript should outline where SmartTrap may not yet be optimal, such as trapping small biomolecules (proteins, RNA), environmental stability requirements, or interaction with complex fluids/media.

4. While the authors do a good job of showcasing the platform with some straightforward standard measurements, to be a real benchmark: (i) it could be valuable to perform a comparison with the output of the same system under manual control, for example, for a few different experienced and less experienced users and obtain information about throughput, error, drift, and variability between operators, and (ii) related to the point above, to compare the resolution of the instrument and other typical performance benchmarks with existing commercial and custom-built setups.

5. How does the system deal with non-spherical targets (e.g. non-spherical cells)? Would this require re-training? It would be good to mention this as a possible limitation or benefit of the setup (depending on how it performs with this).

6. While the open source approach is highly commendable, for publication in Nature Methods, I expect that immediate accessibility is required. I thus recommend that the authors include a GitHub link (with DOI), hardware/electronics schematics, bill-of-materials, software installation instructions, and microfluidic chamber designs with fabrication notes (e.g., 3D printing, PDMS soft lithography) in order to make the entire platform fully open source. If that is not the intention, and the open source aspects are more to allow users to understand what the instrument is doing and maybe be able to make their own modifications to the software elements only, that should be stated in the manuscript.

Minor points:
1. The authors state: “Together, lasers A and B form a single trap where the scattering forces from the two beams cancel out creating a counter-propagating optical trap that is more stable than what a single, highly-focused beam trap would achieve at the same power.”. While this is well established, I recommend providing a citation to support this statement.

2. The abbreviation YOLO is not defined.

3. There is a typographical error: “Examples images from the training dataset, inlcuding the targets, are shown in Suppl. Fig. S2.” should read “Example images.. including”

4. There is a typographical error: “The results were also consistent between different molecules as showed in Suppl. Fig. S5.” Should say “.. as shown in..”

5. In general, the manuscript should be proof-read to ensure that there is consistency in the writing style and grammar. There are parts of the manuscript written in the past tense and parts written in the present tense, for example “We test SmartTrap with this protocol for a sample and have it run continuously for 4.5 h. During this time 938 particles were trapped, 159 of which were large.” Should read “We tested the SmartTrap with this protocol.. and had it run…” to match the rest of the text.

Reviewer #3 (Remarks to the Author):

The authors of “SmartTrap: Automated Precision Experiments with Optical Tweezers” design and build an optical tweezers

platform, which they use to demonstrate automation and repeated execution of various experiments commonly performed with optical traps.

I was overall very impressed with the amount of work that went into this manuscript. The authors developed custom software and hardware for the setup, proposed and implemented multiple automation protocols for different experiments, and made their codes open source. The manuscript is well written, easy to follow, and enough detail is provided for the readers to recreate or adapt the proposed methods. I believe that this work is a very big step towards making optical tweezers more suitable for large scale experiments which require lots of repetitions for data analysis and would be of interest in many different disciplines. I would therefore recommend publication and only have a couple of minor questions/suggestions.

The main limitation, in my view, is that the described setup has only one optical trap. I would like to see some discussion about how easy it would be to extend the automation protocols to e.g. a holographic optical tweezers platform.

The only aspect that I feel I haven't quite understood was that of determining the axial location of the particle. I don't think it's mentioned anywhere how the ground-truth for the axial position was evaluated, if it was evaluated at all. It just wasn't immediately clear to me how well one can trust the predictions made by the neural network. Could the authors provide some information/comments on that?

Version 1:

Decision Letter:

Our ref: NMETH-A61532A

15th Dec 2025

Dear Giovanni,

Thank you for email clarifying the remaining points raised by the reviewers of your revised manuscript "SmartTrap: Automated Precision Experiments with Optical Tweezers" (NMETH-A61532A). We'll be happy in principle to publish it in Nature Methods, pending minor revisions to satisfy the referees' final requests and to comply with our editorial and formatting guidelines.

We are now performing detailed checks on your paper and will send you a checklist detailing our editorial and formatting requirements within two weeks or so. Please do not upload the final materials and make any revisions until you receive this additional information from us.

TRANSPARENT PEER REVIEW
Nature Methods offers a transparent peer review option for new original research manuscripts submitted from 17th February 2021. We encourage increased transparency in peer review by publishing the reviewer comments, author rebuttal letters and editorial decision letters if the authors agree. Such peer review material is made available as a supplementary peer review file. **Please state in the cover letter 'I wish to participate in transparent peer review' if you want to opt in, or 'I do not wish to participate in transparent peer review' if you don't.** Failure to state your preference will result in delays in accepting your manuscript for publication.

Please note: we allow redactions to authors' rebuttal and reviewer comments in the interest of confidentiality. If you are concerned about the release of confidential data, please let us know specifically what information you would like to have removed. Please note that we cannot incorporate redactions for any other reasons. Reviewer names will be published in the peer review files if the reviewer signed the comments to authors, or if reviewers explicitly agree to release their name. For more information, please refer to our FAQ page.

ORCID
IMPORTANT: Non-corresponding authors do not have to link their ORCIDs but are encouraged to do so. Please note that it will not be possible to add/modify ORCIDs at proof. Thus, please let your co-authors know that if they wish to have their ORCID added to the paper they must follow the procedure described in the following link prior to acceptance:
https://www.springernature.com/gp/researchers/orcid/orcid-for-nature-research

Author names using non-Roman characters
Nature Portfolio journals can support presentation of author names using non-Roman characters in the HTML version of the article. If you wish to, please include author names in parentheses after the Roman-character spelling; see example online here. Currently supported scripts are: Arabic, Chinese, Cyrillic, Devanagari, Greek, Hebrew, Hangul, Japanese and Persian. You will be asked to verify the rendering is correct at proof stage.

Thank you again for your interest in Nature Methods. Please do not hesitate to contact me if you have any questions. We will be in touch again soon.

Sincerely yours,
Allison

Allison Doerr, Ph.D.
Chief Editor
Nature Methods

Reviewer #1 (Remarks to the Author):

appreciate that the authors released detailed information about the experimental setup on GitHub. This resolves the issue about missing technical information. However, the license under which the information is published needs to be clarified. An explicit license is missing, both for the control software and the technical drawings. Without an explicit license, the standard rules for copyright apply, which actually forbid that someone else uses, redistributes, or modifies this information. I also noticed that some of the technical drawings mention ‚Adam Karcz‘ as the author, who is not on the list of authors. His contribution should be clarified.

In my opinion, several other issues have not been addressed in a sufficiently satisfactory manner:
A major issue raised in the previous round is the unclear novelty. In the rebuttal, the authors clarified that their main claim on novelty is about the full automatization of the experimental procedures. In my opinion, this makes only a weak point on novelty. Nowadays, where computer-controlled operation of experiments has become standard, this is not an outstanding feature. As an example of prior art, the ‚C-Trap‘ setup from Lumicks provides similar features as the setup presented in the manuscript. Similarly, their ‚bluelake‘ control software has comparable capabilities (but with less fancy video object recognition, not needed for a dual beam setup). Fully automated operation is possible to increase throughput, see https://lumicks.github.io/bluelake-api/2.8.2/index.html
The claim that „… to date, precision measurements, such as experiments with single molecules, single cells, and individual colloidal interactions, have been out of reach for autonomous procedures because of their complexity.“ cannot be sustained.

Another issue raised in the previous round is that the presented implementation of automatized control is tightly bound to a specific custom-made optics and electronic devices, which requires large efforts to replicate. Hence I expect that only a few groups are interested in this innovation and are able to replicate it. The presented setup competes with commercial systems, which are likely more expensive, but actually offer a lower barrier for researchers not experienced with setting up an optical tweezers setup from scratch. In my opinion the broad interest is not given. In this aspect the authors have only given rather unspecific arguments. It seems that if the SmartTrap control software is used without the hardware it belongs to, only few parts can be reused; the object detection and position tracking being the largest part that is independent of the hardware, but also would some effort (retraining) to adapt it to different objects or imaging conditions. On the other side, the custom-made hardware, in particular the optical setup, has rather limited flexibility and it would be difficult to implement signicant modifications. For example, when changing it to a dual beam setup, I see no obvious way to integrate the modified optics within the existing setup without replacing most parts. Any claims related to a broad applicability needs to be more specific. Not everything that can be done is reasonable to do, and specific contribution of this work to possible future developments need to be clearly stated.

I disagree with some arguments that have been newly added to the manuscript: unlike stated (Section ‚The optical tweezers system‘), counterpropagating optical tweezers are not a necessity for light-momentum force detection, nor underfilling of the objective lens aperture. Counter examples are e.g. systems from Impetux, where the only key requirement is high-NA detection of the transmitted light.

Another topic that in my opinion lacks proper discussion is how the presented approach compares with other methods offering higher throughput. This is an important point because the authors emphasize that their setup is well suited for large-scale studies.

For example for DNA/single molecule studies using magnetic fields (with magnetic beads) or acoustic fields (‚acoustic force spectroscopy‘) are alternative methods, that allow parallized experiments for thousands of samples simultaneously, thus offering a much higher throughput than a fully automatized optical tweezers setup ever can reach. Accuracy of these alternative methods is limited by the thermal motion of the particles, there is no fundamental benefit when using optical tweezers. Furthermore, for situations where data quality is limited by individual variations of the samples, often a main limitation in life-sciences, or strong fluctuations due to slow, random processes, e.g. for protein binding studies, accuracy of a single measurement is not of importance, but large sample size is needed to obtain reliable data. The general claim that measurement accuracies of optical tweezers exceed alternative high-throughput approaches is poorly supported and needs more in-depth discussion, explicitly stating which alternative methods exist.

Presentation still needs careful editing required to make the manuscript amenable to readers who are not acquainted with the details of the experimental setup. As mentionen in previous round, the authors talk several times about „the motors“ in the main text without explaining that they are used to translate the microscope stage. This information is still hidden deep in the supplementary information. A reader would need to first read all of the SI to be able to grasp the main manuscript.

In summary, the manuscript and the accompanying released software and technical documents provide very valuable information about years of development, but due to its weak novelty and specialized scope I think that it does not fully meet the requirements for publication in Nature Methods. In any case, the questionable claims need to be tempered and supported with evidence.

Reviewer #2 (Remarks to the Author):

I would like to thank the authors for their thorough rebuttal. Overall, I find that the revisions, clarifications, and substantial expansions to the documentation and software repository address nearly all of my major concerns.

The authors have now added a conflict-of-interest statement acknowledging that one co-author commercially manufactures optical traps. This resolves this particular aspect of the issue of disclosure.

My primary concern was that SmartTrap is only applicable to a specific optical tweezers implementation. The authors have significantly expanded the GitHub repository, including device interfaces, configuration files, example notebooks, and designs. While the platform will still require some effort to adapt to different sample types (e.g. different types and sizes of objects being trapped), the new documentation and training guidance reduce the barrier somewhat.

The authors have made additions to the text which adequately address my concerns regarding performance benchmarking with specific samples. Although the authors cannot provide cross-user benchmarks during this revision cycle, they clarified that SmartTrap performs comparably to experienced users.

Regarding the details I requested for making the setup accessible to the scientific community, the authors now include hardware schematics, bills of materials and extensive software documentation in the GitHub repository. This is an important improvement.

Typographical corrections and definitional clarifications have been made as requested.

Reviewer #3 (Remarks to the Author):

I am satisfied with the Authors' reply to my and other reviewers' comments, and would recommend publication.

Version 2:

Decision Letter:

15th May 2026

Dear Giovanni,

I am pleased to inform you that your Article, "SmartTrap: Automated Precision Experiments with Optical Tweezers", has now been accepted for publication in Nature Methods. The received and accepted dates will be 17 June 2025 and 15 May 2026. This note is intended to let you know what to expect from us over the next month or so, and to let you know where to address any further questions.

Over the next few weeks, your paper will be copyedited to ensure that it conforms to Nature Methods style. Once your paper is typeset, you will receive an email with a link to choose the appropriate publishing options for your paper and our Author Services team will be in touch regarding any additional information that may be required. It is extremely important that you let us know now whether you will be difficult to contact over the next month. If this is the case, we ask that you send us the contact information (email, phone and fax) of someone who will be able to check the proofs and deal with any last-minute problems.

After the grant of rights is completed, you will receive a link to your electronic proof via email with a request to make any corrections within 48 hours. If, when you receive your proof, you cannot meet this deadline, please inform us at rjsproduction@springernature.com immediately.

Authors may need to take specific actions to achieve compliance with funder and institutional open access mandates. If your research is supported by a funder that requires immediate open access (e.g. according to Plan S principles or the NIH public access policy) then you should select the gold OA route, and we will direct you to the compliant route where possible. Because authors warrant under our subscription licensing terms that they haven't committed to licensing any version of their article under a licence inconsistent with the terms of our agreement – including the applicable embargo period – publication under the subscription model isn't suitable for authors whose funders require no embargo.

If you have any questions about our publishing options, costs, Open Access requirements, or our legal forms, please contact ASJournals@springernature.com

If you have posted a preprint on any preprint server, please ensure that the preprint details are updated with a publication reference, including the DOI and a URL to the published version of the article on the journal website.

You may wish to make your media relations office aware of your accepted publication, in case they consider it appropriate to

organize some internal or external publicity. Once your paper has been scheduled you will receive an email confirming the publication details. This is normally 3-4 working days in advance of publication. If you need additional notice of the date and time of publication, please let the production team know when you receive the proof of your article to ensure there is sufficient time to coordinate. Further information on our embargo policies can be found here:
https://www.nature.com/authors/policies/embargo.html

To assist our authors in disseminating their research to the broader community, our SharedIt initiative provides you with a unique shareable link that will allow anyone (with or without a subscription) to read the published article. Recipients of the link with a subscription will also be able to download and print the PDF.

As soon as your article is published, you will receive an automated email with your shareable link.

If you are active on Twitter/X or Bluesky, please e-mail me your and your coauthors' handles so that we may tag you when the paper is published.

You can now use a single sign-on for all your accounts, view the status of all your manuscript submissions and reviews, access usage statistics for your published articles and download a record of your refereeing activity for the Nature journals.

Please note that you and any of your coauthors will be able to order reprints and single copies of the issue containing your article through Nature Portfolio's reprint website, which is located at http://www.nature.com/reprints/author-reprints.html. If there are any questions about reprints please send an email to author-reprints@nature.com and someone will assist you.

Please feel free to contact me if you have questions about any of these points.

Best regards,
Allison

Allison Doerr, Ph.D.
Chief Editor
Nature Methods

** Visit the Springer Nature Editorial and Publishing website at www.springernature.com/editorial-and-publishing-jobs for more information about our career opportunities. If you have any questions please click here.**

Open Access This Peer Review File is licensed under a Creative Commons Attribution 4.0 International License, which permits use, sharing, adaptation, distribution and reproduction in any medium or format, as long as you give appropriate credit to the original author(s) and the source, provide a link to the Creative Commons license, and indicate if changes were made. In cases where reviewers are anonymous, credit should be given to 'Anonymous Referee' and the source.
The images or other third party material in this Peer Review File are included in the article's Creative Commons license, unless indicated otherwise in a credit line to the material. If material is not included in the article's Creative Commons license

and your intended use is not permitted by statutory regulation or exceeds the permitted use, you will need to obtain permission directly from the copyright holder.
To view a copy of this license, visit https://creativecommons.org/licenses/by/4.0/

Reviewer #1 (Remarks to the Author):

This paper presents a custom experimental setup utilizing optical tweezers and associated software. The setup is engineered to automate measurements. For this purpose, it incorporates object detection routines that enable the identification of specific particles and the determination of their three-dimensional positions. Several examples of potential applications and measurement protocols are provided, including the automated measurement of force-extension data for DNA strands. The presented setup constitutes an advanced tool to obtain quantitative information about forces acting on biological samples on the micro and nanoscale.

1. The Nature Methods journal imposes stringent requirements regarding novelty. However, I encounter difficulties in pinpointing the precise and groundbreaking novel contribution of this work. The manuscript appears to be somewhat unclear on this aspect. The optical setup itself appears to be a reimplementation of an earlier design, but only a schematic figure is provided. In my opinion, the description is insufficiently comprehensive and omits crucial details about the setup. A significantly more detailed technical description would be necessary if this were the primary achievement. Also, the benefits and drawbacks of some design decisions need to be discussed in more detail, e.g. I expect that dual trap optical tweezers would show an improved stability of the relative position compared to the presented design, thus relaxing the requirements on the mechanical stability of the setup. Another issue I see is that the presented setup is highly customized, which makes it difficult to replicate, also given the lack of technical details in this manuscript. This limits the broad interest. The basic capabilities of the presented setup compete e.g. with commercially available optical tweezers setups from Impetux or JPK/Bruker with similar features. In which aspects does the presented setup and methods excel, where does it lag behind?

ANSWER: The groundbreaking contribution of SmartTrap lies not in introducing a new optical configuration, but in demonstrating for the first time a fully automated optical tweezers platform capable of independently executing a variety of single-molecule and single-cell experiments without user intervention. This represents a paradigm shift: while both commercial and custom-built optical tweezers rely on manual operation and expert oversight, SmartTrap enables continuous, unattended experiments for many hours, dramatically increasing throughput and reproducibility. Equally important, by releasing all hardware designs, electronics, and software as open-source, SmartTrap democratizes access to advanced optical tweezers technology, lowering the barrier for adoption and customization in laboratories worldwide. Together, automation and open accessibility position SmartTrap as a transformative tool that extends the reach of optical tweezers beyond expert labs, making them practical for large-scale, systematic studies.

The opto-mechanical configuration of the instrument is an straightforward adaptation of the MiniTweezers instrument developed by the Bustamante lab (references [20, 42] in the manuscript). This is an advantage because there are already many research groups that use

this system, which is both a testament to tweezers instruments performance and means there are many who could directly benefit from the SmartTrap developments. We have clarified that it is the same optical tweezers instrument in the Supplementary which now states:

"Importantly, we have designed our own custom circuit boards, firmware, and graphical user interface to get full digital control of the instrument while keeping the same optical system as the original design [20, 42]. Assembly instructions and a component list for the optical tweezers instrument are available from our GitHub repository [43]."

In addition to the original MiniTweezers documentation (reference [42]), we now provide technical drawings, assembly instructions, and full component lists in our GitHub page which makes the system reproducible and accessible to the community. While, as the Reviewer notes, optical tweezers are often customized, our open-source approach (supplying all hardware designs, software, and protocols) lowers the barrier for other laboratories to construct and adapt their own instruments. This is central to the democratizing aspect of SmartTrap.

We also highlight the design decisions that make our setup robust and versatile. The use of counterpropagating optical tweezers enables light-momentum force detection, which directly measures forces acting on the trapped object. This has several advantages over alternative approaches: the force measurement is independent of the particle's exact position relative to the laser focus, robust against variations in experimental conditions, and valid for objects of different shapes (e.g., non-spherical particles or biological cells). The trade-off is a reduction in gradient force, which would make single-beam trapping unstable, but the counterpropagating geometry ensures stable trapping even under these conditions. We have clarified this point in the manuscript as follows:

"Lasers A and B together form a counterpropagating optical tweezers that stably traps particles while directly measuring the trapping force [21]. In this configuration, the laser beam diameters are smaller than the back apertures of the objective lenses. This ensures that all the light passes through the lenses and enables the light-momentum approach to force measurement. Conversely, the gradient force is weaker than when the laser beam is larger than the back aperture, a condition that does not permit trapping with a single beam."

Compared to commercial systems, our platform provides two decisive advantages. First, openness and adaptability: users have full access to the firmware, hardware schematics, and modular software, allowing straightforward customization. For example, one co-author has already adapted the system by integrating a heating laser to study the temperature dependence of DNA elasticity [Rico-Pasto & Ritort, Biophysical Reports 2.3 (2022)]. Second, autonomy: the SmartTrap can execute experimental protocols independently, without user intervention, for extended periods. In our recent tests, it ran continuously for up to 10 hours, limited primarily by particle stability and the evaporation of the objective water (using immersion oil with the same refractive index as water would solve the latter of these two

problems). This capacity for sustained and unsupervised operation is a breakthrough in throughput and reproducibility.

2. The demonstration experiments presented, while showcasing the impressive flexibility of photonic force microscopy, appear to lack novelty. The measurements of drag forces, force-extension data of DNA strands, cell deformation in an optical trap, and electrostatic repulsion between particles, although well-executed, do not introduce new methods.

ANSWER: The reviewer is right: the experiments presented in the manuscript do not introduce new results per se. The novelty lies in the automatization of these experiments rather than in specific results. For this reason, and for showing the robustness of the automatization process, we have chosen well-known experiments as a benchmark, whose methods are well established, and for which it is known what results to expect. By demonstrating many different types of experiments, we aim to demonstrate the versatility of the system. SmartTrap is a high-throughput force spectroscopy tool suitable for characterizing the thermodynamics and kinetics of heterogeneous ensembles of molecular populations. To clarify this point, we have added the following sentence in the introduction of the main text:

"We demonstrate the versatility of SmartTrap across four paradigmatic experiments, which are well-known and widely studied in the literature, but have never been fully automated:"

3. In my assessment, the primary contribution lies in the integration of object detection and position tracking capabilities from camera images into the experiment control system. I commend the software's promise of public availability. This enhancement enables automatic particle capture and monitoring of single particles in optical traps, presenting a valuable feature to the control software. It facilitates the automation of a process traditionally requiring human intervention.
   On the other hand, object detection is a prevalent task in computer vision. Notably, the software utilizes a widely available model (Ultralytics YOLO) for this purpose. While this provides robust particle detection, it appears somewhat over-engineered. Simpler standard computer vision algorithms could have achieved the same task with enhanced computational efficiency, particularly for the presented applications where only spherical particles were utilized.
   Alternative setups employ microfluidics to deliver particles to the optical trap, eliminating the need for advanced image analysis. The performance of the specialized artificial neural networks employed for 3D position tracking remains unclear and warrants further characterization. How does it compare in performance e.g. to fitting Lorenz-Mie theory to inline holograms, as e.g. demonstrated by David Grier's group.

ANSWER: We would like to stress that object detection and position tracking, while essential components of the SmartTrap, are not the central contribution of our work. They should be seen as enabling tools within a larger system whose novelty lies in achieving full automation of optical tweezers experiments through the integration of optics, electronics, software, and control algorithms. Without this synergy, reliable unattended operation for hours would not

be possible. Nevertheless, we emphasize that any tracking and analysis method preferred by the user can now easily be implemented in the SmartTrap framework. For this reason, we have now streamlined the adaption of other methods by providing a specific User Interface for the implementation of custom tracking algorithms. We have added the following sentence to the end of the "Real-Time Image Analysis" section:

"Finally, we would like to highlight that users can easily implement their own real-time image analysis method in the SmartTrap software with an easy-to-use User Interface while maintaining fully automated processes."

Regarding the choice of YOLO, we note that simpler algorithms can indeed detect spherical particles under ideal conditions. However, our system must also detect the micropipette reliably, even when it is out of focus or partially obscured. This is something we found that alternative approaches, such as edge detection or U-Nets, could not achieve consistently. We selected YOLO primarily because it is fast enough for real-time operation while remaining robust under suboptimal imaging conditions. Thus, it was the most practical solution to meet the speed and reliability requirements of automation. To clarify this point, we added the following sentence in the section "Real time image analysis":

"We chose YOLO because it is the best approach we have tried for detecting both the micropipette and the particles."

We also emphasize that particle delivery in SmartTrap cannot rely on microfluidics alone: in experiments where one particle is held in a micropipette, the system must actively detect, track, and align both particles. Advanced image analysis is therefore indispensable.

Finally, regarding 3D tracking, our convolutional network is used only for relative z-alignment of particles into the same focal plane, not for absolute position measurement. For this reason, extensive benchmarking against holographic methods is not necessary, as these serve very different purposes and would require fundamentally different imaging modalities.

4. The authors claim that the full automatization allows for large-scale studies. However, for specific tasks there exist optimized alternative methods that allow throughput orders of magnitudes higher than with single beam optical tweezers, such as microfluidics for measuring cell mechanical properties (see e.g. https://doi.org/10.1016/j.bpj.2015.05.029) or using magnetic or acoustic forces for DNA stretching. This is not properly discussed.

ANSWER: The effort of our paper is to achieve high-throughput with optical tweezers to keep all the advantages of a single-molecule/cell/particle technique that intrinsically high-throughput techniques cannot achieve, such as high spatial and temporal resolution. This high resolution makes optical tweezers the most accurate force spectroscopy technique and is a necessity for characterizing energy processes and thermodynamics at the single molecule level (see reference [40] in main text). Accuracy of about 0.1 kcal/mol is within the range of optical tweezers but beyond that of high-throughput microfluidics and other techniques. To make this point clearer, we have made the following modifications to the introduction section:

“However, optical tweezers' focus on manipulating single objects to achieve high specificity, accuracy, and resolution, an advantage that high-throughput techniques do not have, also represents a significant limitation, as it results in an inherently low throughput.”

“Optical tweezers’ excel at manipulating single objects with high specificity, accuracy, and resolution. This makes them the most precise force spectroscopy technique available and is what enables the direct characterization of energy processes and thermodynamics at the single-molecule level, with measurement accuracies reaching about 0.1 kcal/mol—far exceeding those of alternative high-throughput approaches [16]. However, this inherent focus on single-object manipulation limits throughput and represents one of the main drawbacks of the technique.”

5. The presentation of the methods requires refinement. The fundamental details of the setup are inadequately described. For instance, the authors refer to "the motors" in multiple instances within the text, yet the essential information about their purpose in translating the sample is concealed within the supplemental information. No information regarding the specific type and performance of these motors is given. Another example is found in the description of the DNA pulling experiment, where it is stated that „The micro-controller provides feedback at 7 kHz, much faster than the communication from the host computer, which is why this protocol is run directly on the micro-controller rather than the host offering smoother motion of the lasers.“ However, since feedback from image analysis is also mentioned, it becomes unclear which task precisely is performed by the micro-controller and which by the host computer. Furthermore, the method employed to measure the extension data of Fig. 4 remains unclear.

ANSWER: We thank the reviewer for pointing this out. We have now clarified these points in the revised text.

The motors are Thorlabs dc servo motors z606, which we have clarified in the “Electronics” section of the Supplementary Information in the following sentence:

“The controller samples the various photodetectors, steers the laser wigglers, and controls the motors (Thorlabs Z606 DC Servo Motor) that move the sample.”

Technical details on which hardware and electronic components are used are now provided in the GitHub page (reference [43]).

To avoid confusion about which task is performed by the computer and which by the microcontroller, we clarify that the image analysis is done by the host computer and used by it with the other data (laser position, force, etc.) to decide what is the next step to perform. After this, the host computer sends the list of commands to the microcontroller to perform, such as moving the lasers. We have modified the main text to clarify this:

“This enables us to implement simple feedback algorithms directly on the controller, for example to move the trap at constant speed until a certain force value is reached.”

“In order to automate the experimental procedures, it is essential to analyze the video feed from the camera. We implemented this analysis on the host computer using deep learning because of its precision, versatility, and speed [21].”

“The processed information is used by the host computer to detect events (e.g., a particle entering the trap)”

In addition, we would like to clarify that both the host computer and the microcontroller use feedback. However, the microcontroller only performs simple feedback tasks, such as the “automatic alignment of counter-propagating traps” described in the supplementary information and the movement of the lasers at constant speed during a DNA pulling experiment. More advanced feedback processes, such as the one described above, are handled by the host computer. To clarify this, we have also modified the following paragraph in the supplementary section “DNA pulling”:

“Finally, a pulling protocol is loaded onto the micro-controller. This protocol is a set of simple instructions that are repeated by the controller: moving the optical trap between two positions at a set speed to pull the molecule. The protocol is run on the micro-controller because it can update the trap position at a rate of 7 kHz, much faster than the communication with the host computer, ensuring a smooth motion of the trap. To find the upper limit of the separation, i.e., the maximum distance between the particle in the pipette and the particle trapped by laser, an upper force threshold is set, while a minimum separation distance between the particles is set to a specific value. Once this pulling protocol starts, the host computer also starts recording data. The protocol will repeatedly separate the particles to stretch the molecule and thereafter let it relax. This is repeated for a user specified duration of time, which we have set to 3 minutes.”

Regarding the last point of this comment, the extension data of Fig. 4 shows the molecule extension, which equals the particle position plus an offset coming from the laser position being measured relative to the center of the detectors. The particle position is derived from the measurement of the laser position using the position detectors and then combining this with an optical trap stiffness measurement. As stated in the caption of Fig. 4 and in the main text, the trace is aligned with the WLC model to have the same extension at a force of 30 pN, accounting for the position offset. The length of the DNA fragments is known from the sample preparation processes. We have added the following paragraph in the supplementary information to explain how the extension is calculated.

“To determine the particle position, and thus the molecular extension, we use real-time tracking. However, since YOLO is noisier and slower than the position-sensitive detectors (PSDs), we employ YOLO only to estimate the optical trap stiffness. The stiffness of the optical trap depends on the size of the particles (which varies from particle to particle) and is required for the force-extension analysis. Specifically, we compare the force-extension signal when using the particle position (obtained from YOLO) to the force-extension signal when using the trap position (obtained from the PSDs). The difference between these two signals reflects the

displacement of the particle relative to the center of the trap, which is caused by the molecule pulling on the particle. This displacement is linear with the applied force in the force range of the experiment. The linearity of the trap up to ca 80 pN was verified separately by trapping particles of similar size and gradually increasing the flow while monitoring their displacement. By fitting the force to the difference between the trap position and the position of the particle, we can determine the stiffness of the trap, without having to perform additional calibration measurements on each particle before each experiment, thus obtaining the molecular extension from the PSD signals."

6. In addition, I have reviewed the SmartTrap control software, which provides a comprehensive and user-friendly platform for experiment control. However, from a software engineering perspective, its limited modularization and lack of abstraction layers present challenges regarding its reusability for diverse purposes beyond its initial design. For example, the software integrates user interface management, low-level hardware control, and control logic at the same level, which impedes its adaptability. Furthermore, its close coupling to specific hardware restricts its broader applicability.

ANSWER: We thank the Reviewer for raising these issues. We have proceeded to restructure the code and structure of the GitHub page to address these issues and make the code more developer-friendly and adaptable to other systems. Specifically, we have:

- Separated control of the different devices and processes into independent classes.
- Introduced interfaces for the various hardware devices (camera, laser power supply, microfluidics, etc.) as well as for the object tracking.
- Extended the documentation.
- Updated the GitHub page to contain more thorough descriptions of the various parts of the software and with instructions on what needs to be changed to accommodate different updates.
- Added several example notebooks which demonstrate how the different devices can be used, also without the main program.
- Introduced a configuration file that helps users to set up the software in accordance with their hardware.
- Added a 3D animated video to show clearly how the setup is composed and of which elements.
- Simplified installation by providing an installation script which can be run to install the necessary python packages.

Together these updates simplify the adaptation of the program to other hardware, e.g., cameras or microfluidics pumps from different manufacturers and make it easier for others to build on the software with additional functionality. Below is also a screenshot from the relevant part of the updated GitHub main page.

Controllers and drivers

The SmartTrap system combines a central tweezers controller with several independent devices, each with its own hardware controller and corresponding Python driver. All drivers follow a Python protocol, so replacing hardware requires only implementing the correct interface and updating the create_devices function in the smarttrap_config.py file. Further details can be found in the Software folder.

- **Optical tweezers instrument** - The instrument is controlled using the custom controller. This provides also control of the motors, laser movement and sensor readings.
  - Hardware: Custom electronics controller. Controls the motors, laser positioning and reads the various photosensors.
  - Driver: Communicates with the host computer with serial USB. The different subsystems, sample stage motors, laser actuators and photosensors, are each controlled by separate classes allowing them to be easily replaced without replacing the entire controller if need be.
- **Camera** - Camera is monitored directly in the user interface and from this one can also record videos and the camera feed is used in the autonomous protocols.
  - Hardware: Two options currently implemented: Thorlabs scientific cameras and Basler cameras.
  - Driver: Connects automatically to the camera from the chosen suppliers. Other cameras can be implemented using the interface in camera_controls. The Basler cameras requires the PyPylon package while the Thorlabs cameras require installing the thorlabs SDK
- **Laser power control** - Enables adjusting the power of the two lasers directly from the user interface as well as during autonomous protocols
  - Hardware: Uses an OSTech laser diode driver and TEC controller ds11-la0.5v14-pa02v14-t8545-691
  - Driver: Uses serial communications, integrated in the OSTech laser
- **Microfluidics** - The microfluidics is used to deliver particles into the system and digital control is necessary for autonomous procedures.
  - Hardware: Pump and valves used are from ElveFlow, in particular the OB1 microfluidics pump and the Mux Wire V3 valve controller.
  - Driver: Requires ElveFlows proprietary software as well as the the LabView runtime engine
- **Micropipette** The suction of the micropipette can be toggled by activating the pump connected to it.
  - Hardware: Uses a pump powered by a dc motor, D2028B from SparkFun Electronics. This is controlled by a separate power supply, TENMA 72-2540
  - Driver: Python driver with the ability to set the power of the pump and toggle the suction on and off.
- **Motorized objective movement**- This is an optional addition and allows the user to move the objectives from the user interface for adjusting focus.
  - Hardware: Is an arduino UNO. The program for it can be found in the firmware folder.
  - Driver: Python driver communicating using serial USB. Allows for moving the objective in either long or short steps closer to or away from the chamber.
- **Pipette puller** A separate device used to make the micropipettes by heating and pulling on glass microcapillaries.
  - Hardware: Custom desgined, see pipette puller section. Powered by a TENMA 72-2540 power supply.
  - Driver: Runs in a separate graphical user interface in which the parameters of the heating can be tuned to get suitable size of the pipettes.

In my assessment, while the manuscript possesses certain notable features, it falls short of meeting the essential criteria for publication in Nature Methods. Specifically, it lacks the broad interest, novelty, and comprehensive technical description that are crucial for this esteemed journal.

ANSWER: The revised the manuscript makes it clear how SmartTrap provides both novelty, through the first demonstration of fully automated execution of multiple optical tweezers experiments, and broad interest by enabling researchers from diverse fields (single-molecule biophysics, cell mechanics, colloidal science) to access autonomous high-resolution force measurements. We have now substantially expanded the technical description by adding details on the optical setup, electronics, and microcontroller/host computer tasks, while also restructuring and documenting the software to make it more modular and adaptable. Together, these additions clarify the uniqueness of our approach and ensure reproducibility. Equally important, we now show the capacity of the system to operate for long times and gather large amounts of data. In this way, we have developed a system that can overcome the challenge of optical tweezers in collecting large datasets improving the most accurate force

spectroscopy method so that it is also high throughput. Therefore, the revised manuscript now addresses the reviewer’s concerns and we believe it meets the criteria for publication in *Nature Methods*.

Reviewer #2 (Remarks to the Author):

The authors describe the SmartTrap, an integrated, open-source optical tweezers platform that combines deep learning–based 3D particle tracking, microfluidics, and custom electronics to automate single-cell and single-molecule assays (e.g., such as DNA force-extension measurements and red blood cell deformation). The text and associated videos demonstrate autonomous execution with fast hardware responses and multi-hour continuous operation

To start, I would like to say that I appreciate the motivation behind the manuscript – single-molecule and single-cell optical tweezers measurements could indeed greatly benefit from increased throughput. I also very much appreciate the open-source approach presented here and think that if it is broadly adopted, SmartTrap could indeed push forward the next generation of optical tweezers research. The integration of advanced AI-based tracking with fast feedback loops and custom microfluidics and its successful demonstration using some standard protocols (DNA pulling, red blood cell stretching, measurement of electrostatic interactions between two particles) shows that SmartTrap goes beyond simply being an incremental development in the field.

ANSWER: We thank the Reviewer for recognizing that SmartTrap "goes beyond simply being an incremental development in the field" and that it can have a significant impact in the field by increasing experimental throughput thanks to its automation and by democratizing access to advanced tools thanks to its open-access nature.

Taking this into account, I found the described technique to be suitable for publication in Nature Methods, but I do have some points that I would like the authors to address.

ANSWER: We thank the Reviewer for finding the SmartTrap suitable for publication. We added detailed answers to the raised points below.

Major points:

1. *The manuscript does not currently include a "Conflict of Interest" statement. I note that one of the co authors, Steven B. Smith, is known to make and sell optical tweezers systems. This commercial tie should be explicitly disclosed according to Nature Methods guidelines, or otherwise commented upon if deemed irrelevant.*

ANSWER: We thank the referee for raising this important point. For the sake of transparency, we have added the following conflict of interest statement:

"Steven B. Smith makes and sells optical traps. The authors declare no other conflicts of interest."

Nevertheless, we note that, while Steven B. Smith is involved in the commercial development and sale of optical tweezers systems, these systems do not incorporate the features or capabilities presented in this manuscript, namely, the SmartTrap electronics and automation technology.

2. *A key limitation of the SmartTrap is its strict dependence on a specific optical-tweezers configuration. Both the experimental protocols and deep-learning models are calibrated and trained exclusively on this setup. Thus, labs with even slight variations in optics, cameras, microfluidics, or electronics would need substantial retraining and reconfiguration before the platform functions, limiting its immediate generalisability. It would be appropriate to highlight this point, clarifying that while technically impressive, SmartTrap currently addresses a small community of labs and may require significant resources and custom integration to be adopted by others. I would encourage the authors to discuss how much effort (e.g., both in terms of model training and actual experimental setup) would be necessary to adapt the platform to other hardware, or whether the idea is rather that labs purchase their own SmartTraps (this is related to point 1, above).*

ANSWER: As the Reviewer correctly points out, the SmartTrap software is not "plug-and-play" for other optical tweezers systems as it was developed for a specific setup, which means that other users need to adapt the software in order to gain access to its advanced functionalities. To mitigate this issue, in the revised version, we have made major updates to the GitHub repository, to provide all details for how to build and operate the optical setup, as well as to the code to make adaptation of the software to other systems as smoothly as possible.

To make it easier for others to build their own system or take inspiration from ours, we now also provide component lists, assembly instructions, and detailed descriptions of the hardware on the GitHub page. Furthermore, a 3D animated video is provided to show where the parts are in the instrument.

Regarding the code, we have extensively revised it to make it more modular, better documented, and more easily adapted to other experimental setups. Specifically, we have:

- Separated the control of the different devices and processes into independent classes.
- Introduced interfaces for the various hardware devices (camera, laser power supply, microfluidics, etc.) with test example codes to check that they are working correctly.
- Extended the documentation with docstrings describing the various classes and their functionality.
- Updated the GitHub page to contain more thorough descriptions of the various parts of the software and added instructions on what needs to be changed to accommodate different updates.
- Added several example notebooks which demonstrate how the different devices can be used, also without the main program.
- Simplified installation by providing an installation script that can be run to install the necessary Python packages.
- Introduced a configuration file that helps users to set up the software in accordance with their hardware.
- Introduced a "testmode" which allows users to run the interface without any device connected to test various features and user interface elements.

With these updates, adapting the software to other systems requires only minor efforts. Specifically, the user needs to implement the interfaces for their hardware and update the software configuration file. Then, the user can run our software and its graphical user interface (GUI), in combination with its features (real-time plotting of forces and positions, particle tracking, automatization), even if their hardware differs drastically from the SmartTrap. Moreover, the user can relatively easily generalize the automatization processes, since we have extracted autonomous protocols into separate classes (one per experiment type) and introduced callable functions for the different sub-processes, such as trapping. In this way, also the adaptation to new experimental configurations, such as a system that does not use a pipette or that uses multiple optical traps, is simplified.

Regarding the model training, the deep learning algorithms, especially YOLO, are quite resilient to changes in the optical configuration (e.g., how the image of the particle appears) and re-training is required only if the optical configuration of the setup strongly differs (e.g., if the new system uses holographic video microscopy instead of normal bright-field or dark-field) from the one that we present in the manuscript. To demonstrate this resiliency, we have used the model with other cameras (different field of view and dynamics) and different kinds of particles (silica, polystyrene, melamine resin): the tracking still works well but is slightly less accurate. The script used for this demonstration is available on our GitHub page under Software/Examples, allowing users to test the performance of the tracking for their own applications without installing the entire software. Below are two example images from completely different systems. The first image is from a holographic optical tweezers trapping multiple silica particles (ca 4 microns in diameter), along with some of the z-position predictions. The second image shows MF particles (ca 2 microns in diameter).

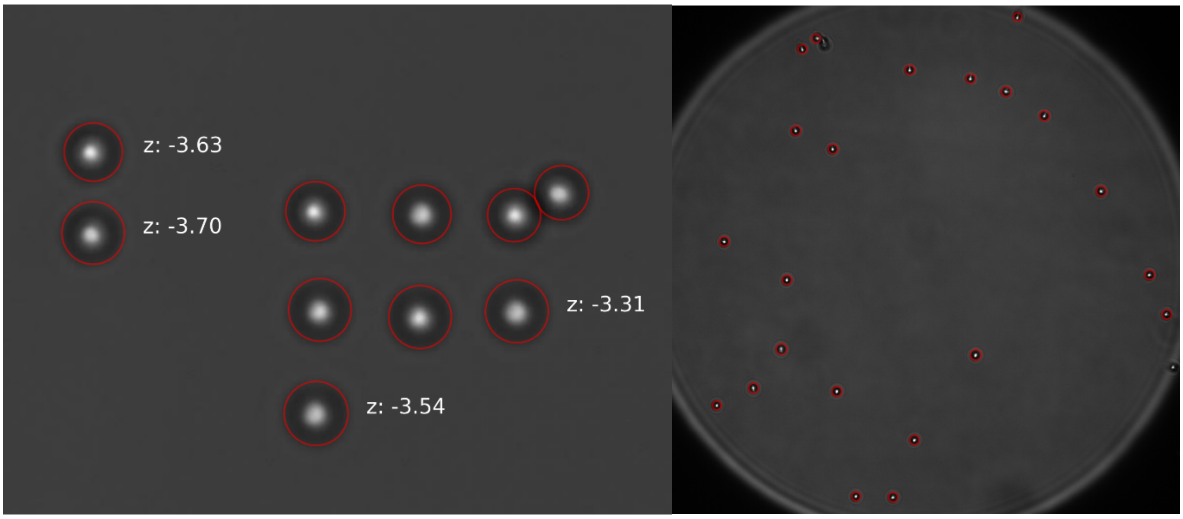

If high accuracy is needed, or the user wants to track complex objects (e.g., non-spherical particles, bacteria, cells), the user needs to retrain the model with experimental images from their system. In our experience, the whole retraining requires a few days of effort, most of which is spent acquiring experimental images, while the computational time of the network

training only takes a few hours. Also, we have provided a quick guide to how to do this (with YOLO) on the GitHub page under Software/NeuralNetworks. Finally, in case the user already has a finished tracking algorithm, they can use their own tracking methods by simply implementing the provided interface, making them near plug and play.

We have updated the supplementary section Artificial Neural Networks to make readers aware of this adaptability.

"We would also like to highlight that replacing or adapting the tracking methods, e.g. to trap bacteria instead of spherical particles, is straightforward. One simply needs to implement the interface provided in our GitHub repository [43]."

3. *The data shown in Figure S5 and Figure 4 of the DNA stretching seem to show quite some noise/deviation from the WLC and the stretching and refolding curves do not match up perfectly, as I would expect them to, in Figure 4. The data in Figure S5 are very zoomed out so it is difficult to judge the quality of the data well. Can the authors comment on the force and distance resolution of the instrument (in the manuscript) and provide a close-up of the data in S5 in the distance range of 3 – 4.5 μm (for my own inspection)? It seems important to mention that many single-molecule experiments performed using the optical tweezers require excellent, repeatable high-resolution data and that knowledge of the limitations of the instrument is important to assess how broadly it can be applied for studies of DNA-protein interactions, RNA and protein folding. The manuscript should outline where SmartTrap may not yet be optimal, such as trapping small biomolecules (proteins, RNA), environmental stability requirements, or interaction with complex fluids/media.*

ANSWER: The force resolution of the instrument is 0.04 pN, while the distance resolution is 1 nm when using the PSD sensors and about 10 nm when using video microscopy (this value also depends on the properties of the particle to be tracked). To clarify this point, we have added the following sentence in the supplementary section "Optical Tweezers System":

"In this way, the SmartTrap achieves a force resolution of 0.04 pN and a distance resolution of 1 nm when using the PSD sensors and about 10 nm when using video microscopy."

As requested by the reviewer, we attached here a zoom-in of Figure S-5 in the range 3-4.5 µm. We have also added this graph to the manuscript in the supplementary information.[1] Below is

[1] As part of the review process, we ran the experiment again and tested how long the instrument could operate continuously, which is why we now have new data in the figures. Previously, we used the YOLO algorithm to directly extract the particle position, as it runs in real-time. Even if this is very convenient to fast check the experimental outcomes, it can be noisy, especially when looking at individual detections. Therefore, we now use the PSD position detectors instead and use the real-time tracking to calibrate the tweezers for each particle. Specifically, we ensure that the force–extension curve derived from the PSDs matches that obtained from tracking, assuming a linear relationship between the applied force and the particle's displacement from the trap center—an assumption we verified by trapping particles and gradually increasing the force through controlled flow. Furthermore, we slightly updated the autonomous pulling algorithm to account for the fact that the molecule may not attach at the top of the particle in the pipette. It does so by first stretching the molecule up

a zoomed-in version of Figure S-5, which we have also added to the manuscript. We would also like to point out that S-5 now contains over 500 different stretching traces from 17 different molecules, all collected in the same continuous and autonomous run of the experiment.

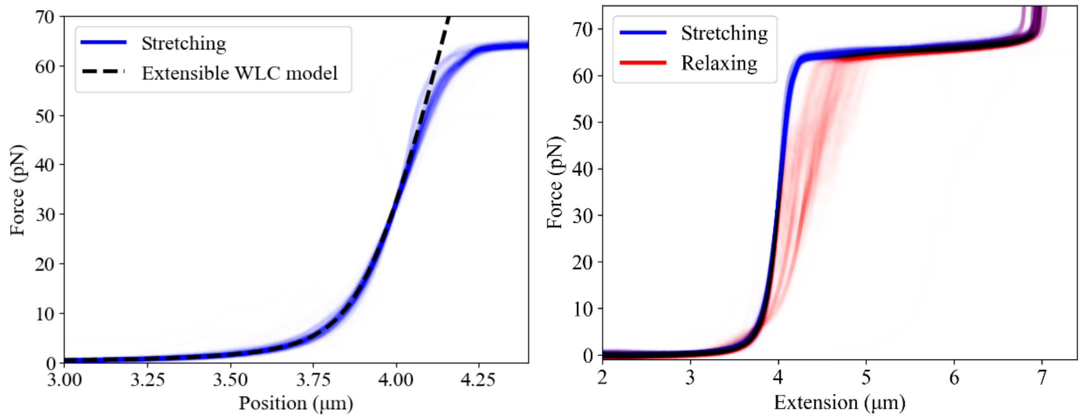

The stretching and relaxing curves mostly align well. However, there is hysteresis, caused by fraying of the double helix at places where the chains are nicked. Usually, this nicking occurs during the preparation of the DNA and not during the actual pulling. This causes some deviations between the stretching and relaxing curves, on occasion, also at low forces. The nicking can also affect the stretching if the nicks do not re-anneal before the next pull is initiated. Below is also the updated graph of Fig. 4, which uses the PSDs instead of the real-time tracking.

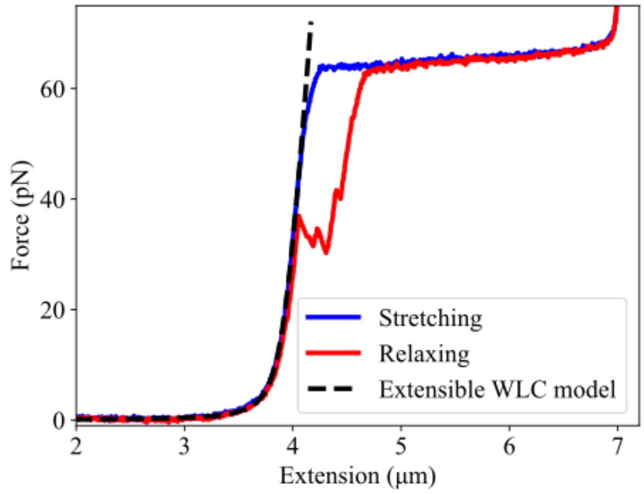

To improve the data quality, one solution is to trap smaller particles to improve the performance of the *automatic alignment of counter-propagating traps*. However, smaller particles are trapped less firmly, generating noise that reduces the quality of the data much more than the protocol benefits.

to a force of ca 60 pN and then adjusting the particle position to reduce the force readings along the axes perpendicular to the pulling direction.

Finally, we have not quantified the environmental stability requirements. The minitweezers instrument on which the SmartTrap is based is well-known, characterized, and widely used in literature for many different single-molecule experiments. Furthermore, since the optical tweezers instrument is rather small, it can easily be moved and placed in an environmentally controlled chamber. Indeed, Steven B. Smith and Felix Ritort, who have the same optical tweezers system, have placed the entire tweezers inside a fridge to conduct multiple experiments at temperatures ranging from 5°C–50°C, see: De Lorenzo, S., Ribezzi-Crivellari, M., Arias-Gonzalez, J. R., Smith, S. B., & Ritort, F. (2015). A temperature-jump optical trap for single-molecule manipulation. *Biophysical journal*, *108*(12), 2854-2864 and Rico-Pasto, Marc, and Felix Ritort. "Temperature-dependent elastic properties of DNA." *Biophysical Reports* 2.3 (2022). To underline this, and the fact that the optical tweezers instrument was designed for single-molecule force spectroscopy studies rather than direct trapping of single molecules or very small particles, we have added the following in the supplementary section Optical Tweezers System:

"The instrument was designed for single-molecule force spectroscopy studies and can trap particles down to ca 0.5 µm in diameter. It has a small footprint of ca. 30 cm across, which allows it to be easily moved and placed within environmentally controlled chambers."

4. *While the authors do a good job of showcasing the platform with some straightforward standard measurements, to be a real benchmark: (i) it could be valuable to perform a comparison with the output of the same system under manual control, for example, for a few different experienced and less experienced users and obtain information about throughput, error, drift, and variability between operators, and (ii) related to the point above, to compare the resolution of the instrument and other typical performance benchmarks with existing commercial and custom-built setups.*

ANSWER: We thank the Reviewer for this important comment. We have indeed compared the system performance with the "manual" performance of several of the authors who are very experienced using the same setup. SmartTrap performs as well as a well-trained human repeating the experiments always in the same way, but it does so tirelessly. (Anecdotally, we also started relying on the autonomous procedures as soon as they were implemented. For instance, when working on the procedure for autonomously attaching DNA, we used the already implemented trapping protocol to collect new particles to test, finding this process to be faster than collecting them manually.)

Furthermore, during the review process, we tested how long the system could run independently and found that it could operate for over 10 hours. What limits the operation is primarily the evaporation of the water used for the immersion objectives and the aggregation and sedimentation of the particles (biomolecules may also degrade at room temperature).

We plan on performing benchmarks with different users, especially with unexperienced users. Unfortunately, this is a very lengthy and time-consuming process that requires the use of the

instrument for long periods of time and the availability of many users to be successfully and meaningfully performed. Therefore, we cannot provide this data within this review process.

5. *How does the system deal with non-spherical targets (e.g. non-spherical cells)? Would this require re-training? It would be good to mention this as a possible limitation or benefit of the setup (depending on how it performs with this).*

ANSWER: The SmartTrap can handle non-spherical particles without any practical issues. However, retraining the tracking models may be necessary to enable the SmartTrap to handle the new properties of the particles autonomously, particularly during the phase when the SmartTrap collects the particles for experimentation. Apart from this point, the automation processes do not require further modification to work. YOLO can also be trained to detect and differentiate multiple different particles or objects as demonstrated by the use of the same network to detect both particles and pipette. To clarify this point we added the following sentence in the Real Time Image Analysis section:

"Thanks to the versatility of this approach, the SmartTrap can easily be made to handle also non-spherical particles or objects (as illustrated by the pipette detection), requiring only a quick retraining of the YOLO network."

Importantly, since the SmartTrap measures the force acting on the trapped particle directly, the force measurement is independent of the particle shape, making the system well-suited for measurements of non-spherical particles. One could, for instance, use it to measure the swimming force of bacteria. We now clarify this advantage with the following addition to the discussion:

"Also, since the force measurement is independent of object shape, it can be used to measure forces on non-spherical objects such as bacteria or plankton."

6. *While the open source approach is highly commendable, for publication in Nature Methods, I expect that immediate accessibility is required. I thus recommend that the authors include a GitHub link (with DOI), hardware/electronics schematics, bill-of-materials, software installation instructions, and microfluidic chamber designs with fabrication notes (e.g., 3D printing, PDMS soft lithography) in order to make the entire platform fully open source. If that is not the intention, and the open source aspects are more to allow users to understand what the instrument is doing and maybe be able to make their own modifications to the software elements only, that should be stated in the manuscript.*

ANSWER: We thank the Reviewer for raising this important point. The intention is indeed to make the entire platform open-source and accessible to the scientific community. We have therefore revised and polished the GitHub repository accordingly, accessible at https://github.com/softmatterlab/SmartTrap (already referenced in the manuscript as Reference [43]).

We have added information in the GitHub repository to make the SmartTrap hardware easy for others to replicate. Specifically, we have updated the repository to further describe the hardware by including the following:

- Lists of components of the instrument (including bill-of-materials for the controllers).
- Simplified assembly instructions for the instrument with an animated 3D video that shows where the parts are located and installed.
- Assembly instructions for the custom pipette puller, including component list and usage instructions.
- Assembly and installation instructions for the microfluidics chamber, with pictures to guide users through the various steps (see screenshot below for the first few steps).
- Extended the documentation of the software and how to adapt it to other hardware.

Finally, to make the reference to the original MiniTweezers webpage clearer, we have modified it from "[42] S. B. Smith and C. Rivetti, Tweezerslab, (Accesed 2025)" to "[42] S. B. Smith and C. Rivetti, Tweezerslab, http://tweezerslab.unipr.it/cgi-bin/home.pl"

Minor points:

1. *The authors state: "Together, lasers A and B form a single trap where the scattering forces from the two beams cancel out creating a counter-propagating optical trap that is more stable than what a single, highly-focused beam trap would achieve at the same power.". While this is well established, I recommend providing a citation to support this statement.*

ANSWER: We thank the reviewer for this comment. Unfortunately, there are no published comparisons for the trapping stability of counter-propagating traps vs. single-beam traps. However, there is a useful parameter called "trapping efficiency Q" which relates the escape force F (maximum holding force, either axial or transverse) to the laser beam power W, speed of light c, and the surrounding fluid refractive index n. From W. H. Wright, G. J. Sonek, M. W. Berns,"Parametric study of the forces on microspheres held by optical tweezers", Applied Optics 33: 1735-1748 (1994), the escape force F is related to the trapping efficiency Q in this way:

$F = Q\, n\, W / c$.

Therefore, Q helps to quantify how strong the trap is without caring about the optical configuration: a high Q value means a stronger trap for a given laser power.

Table 4 of Wright et al. reports the average transverse Q for a 5-um polystyrene bead in a single-beam trap as 0.20, but Table 1 of Smith et al (2003) shows a transverse Q of 0.39 for the same size bead in a counter-propagating trap. The total power W was taken as a sum of both beams. The difference in Q values arises from the greater aligned momentum of low NA trap beam rays compared with a higher NA converging beam, where most rays sit at a high angle to the optic axis.

We modified the sentence to clarify this point and we added the reference [20] (S.B. Smith, Y. Cui, C. Bustamante, "Optical-trap force transducer that operates by direct measurement of light momentum" Methods in Enzymology 361:134-162 (2003), reference already cited in the manuscript):

“Lasers A and B together form a counterpropagating optical tweezers that stably trap particles while directly measuring the trapping force [21]. In this configuration, the laser beam diameters are smaller than the back apertures of the objective lenses. This ensures that all the light passes through the lenses and enables the light-momentum approach to force measurement. Conversely, the gradient force is weaker than when the laser beam is larger than the back aperture, a condition that does not permit trapping with a single beam.”

2. *The abbreviation YOLO is not defined.*

ANSWER: We thank the Reviewer for pointing this out. We added the YOLO abbreviation to the main text the first time we are referencing to it, specifically in this sentence:

“This is done using an artificial neural network that utilizes the YOLO, You Only Look Once, object detection framework [22] and, in particular, YOLO V5 [23].”

3. *There is a typographical error: “Examples images from the training dataset, inlcuding the targets, are shown in Suppl. Fig. S2.” should read “Example images.. including”*

ANSWER: We thank the Reviewer. We have now fixed this typo.

4. *There is a typographical error: “The results were also consistent between different molecules as showed in Suppl. Fig. S5.” Should say “.. as shown in..”*

ANSWER: We thank the Reviewer and we fixed this error.

5. *In general, the manuscript should be proof-read to ensure that there is consistency in the writing style and grammar. There are parts of the manuscript written in the past tense and parts written in the present tense, for example “We test SmartTrap with this protocol for a sample and have it run continuously for 4.5 h. During this time 938 particles were trapped, 159 of which were large.” Should read “We tested the SmartTrap with this protocol.. and had it run…” to match the rest of the text.*

We *thank the Reviewer for pointing this out, and we have proceeded to make the writing style and grammar consistent.*

Reviewer #3 (Remarks to the Author):

The authors of "SmartTrap: Automated Precision Experiments with Optical Tweezers" design and build an optical tweezers platform, which they use to demonstrate automation and repeated execution of various experiments commonly performed with optical traps.

I was overall very impressed with the amount of work that went into this manuscript. The authors developed custom software and hardware for the setup, proposed and implemented multiple automation protocols for different experiments, and made their codes open source. The manuscript is well written, easy to follow, and enough detail is provided for the readers to recreate or adapt the proposed methods. I believe that this work is a very big step towards making optical tweezers more suitable for large scale experiments which require lots of repetitions for data analysis and would be of interest in many different disciplines. I would therefore recommend publication and only have a couple of minor questions/suggestions.

The main limitation, in my view, is that the described setup has only one optical trap. I would like to see some discussion about how easy it would be to extend the automation protocols to e.g. a holographic optical tweezers platform.

ANSWER: We thank the Reviewer for emphasizing the significance of our efforts to adapt optical tweezers for large-scale experiments.

Regarding the automation of multiple-trap optical tweezers, we do not foresee any conceptual issues or major obstacles. Our software can be adapted for multiple-trap optical tweezers, and controlling the diffractive optical element can be implemented without issue. The automation protocols of the SmartTrap already address two trapped particles: one in the pipette (mechanical trap) and one in the optical trap. Therefore, extending these protocols to two optical traps is quite straightforward. Moreover, as a counterpropagating tweezers system, the SmartTrap already has two independently controlled lasers, which easily adapts to a dual trap system from a software point of view. Indeed, this was tried in the labs of Felix Ritort with this optical tweezers system for investigating fluctuation relations by trapping two silica particles, one per laser, see Ribezzi-Crivellari, M., & Ritort, F. (2014). Free-energy inference from partial work measurements in small systems. *Proceedings of the National Academy of Sciences*, *111*(33), E3386-E3394. The use of silica particles is necessary because the usage of direct force measurement prevents overfilling of the objectives. By using silica particles instead of polystyrene, one can reduce the scattering force, which otherwise prevents stable trapping by a single low-NA laser. To clarify this point, we added the following sentence to the Discussion section:

"Nonetheless, extending the automation processes to double-trap optical tweezers or holographic optical tweezers is quite straightforward. Considering that the pipette is a mechanical trap and the SmartTrap already uses two counterpropagating laser beams, adapting from an instrument with an optical and mechanical trap to a system with two optical traps requires no major software modifications."

In terms of automation, using multiple traps removes the need to align in the z-direction when performing measurements with two or more particles, which simplifies automation of certain experiments. Also, many parts of the autonomous process remain the same also in multi-trap systems, such as trapping of particles and the attachment and detection of molecules, one just needs to have the "second particle" in another optical trap instead of on top of the micropipette. For another project unrelated to this paper, we already used some of the same algorithms of the SmartTrap to automate trapping in holographic optical tweezers.

The only aspect that I feel I haven't quite understood was that of determining the axial location of the particle. I don't think it's mentioned anywhere how the ground-truth for the axial position was evaluated, if it was evaluated at all. It just wasn't immediately clear to me how well one can trust the predictions made by the neural network. Could the authors provide some information/comments on that?

ANSWER: We thank the Reviewer for making us aware that this point was unclear in the manuscript. Since the main purpose of the algorithm is to align particles so that they are on the same z-plane, the ground truth with the absolute z position is not needed. For z- alignment, one only needs to know if the particles are in the same plane and, if they are not, in which direction they should be moved.

Obtaining the absolute position is an interesting feature, but complex and not needed for our current purposes and the experiment performed. Moreover, we would like to point out that we have not yet implemented absolute z-position detection because, even if very interesting and potentially useful, we would need a more precise method for moving along the z-axis (e.g. a piezo actuator) to verify the accuracy.

We have clarified this aspect of the tracking by adding the following sentence in the supplementary section "Real time image analysis":

"The network predicts the position of the particles in arbitrary units allowing the alignment feedback algorithm to match the predictions of the particle in the trap to that held by the pipette by moving the sample along the z-direction."

Outline of Reply to Reviewers (NMETH-A61532A)

We are grateful that Reviewers #2 and #3 found our revisions and clarifications to be satisfactory and that they consider the manuscript ready for publication. In the following pages, we provide a structured, point-by-point response to the remaining concerns, mostly raised by Reviewer #1.

Reviewer #1:

Remarks to the Author:
appreciate that the authors released detailed information about the experimental setup on GitHub. This resolves the issue about missing technical information. However, the license under which the information is published needs to be clarified. An explicit license is missing, both for the control software and the technical drawings. Without an explicit license, the standard rules for copyright apply, which actually forbid that someone else uses, redistributes, or modifies this information. I also noticed that some of the technical drawings mention ‚Adam Karcz‘ as the author, who is not on the list of authors. His contribution should be clarified.

We release the entire SmartTrap repository on Zenodo under an MIT License, a highly permissive open-source license chosen specifically to maximize community reuse, modification, and adoption. The license is now added to the GitHub repository and the repository is released on Zenodo.

Regarding the drawings attributed to Adam Karcz, we apologize for the lack of clarity. His name appears on several technical schematics because he assisted with their preparation as part of his technical role in the laboratory of Prof. Carlos Bustamante (who is a co-author of the work) that originally developed the setup hardware. His contributions were limited to drafting these drawings and did not involve conceptual or scientific contributions to the development of the SmartTrap platform. Still, we have removed these files and replaced them with updated CAD models.

As a side note, we appreciate that Reviewer #1 agrees that the missing technical information is now available.

In my opinion, several other issues have not been addressed in a sufficiently satisfactory manner:
A major issue raised in the previous round is the unclear novelty. In the rebuttal, the authors clarified that their main claim on novelty is about the full automatization of the experimental procedures. In my opinion, this makes only a weak point on novelty. Nowadays, where computer-controlled operation of experiments has become standard, this is not an outstanding feature. As an example of prior art, the ‚C-Trap‘ setup from Lumicks provides similar features as the setup presented in the manuscript. Similarly, their ‚bluelake‘ control software has comparable capabilities (but with less fancy video object recognition, not needed for a dual beam setup). Fully automated operation is possible to increase throughput, see https://lumicks.github.io/bluelake-api/2.8.2/index.html
The claim that „… to date, precision measurements, such as experiments with single

molecules, single cells, and individual colloidal interactions, have been out of reach for autonomous procedures because of their complexity." cannot be sustained.

We respectfully maintain that the primary novelty of our work lies in enabling fully autonomous, long-duration, precision measurements with optical tweezers (enabled by the development of new electronics and software), a capability for which we have not found any peer-reviewed, published demonstrations in the literature. To the best of our knowledge, no existing work has shown autonomous operation that can reliably manage the full experimental workflow, adapt to contingencies during execution, and maintain stable performance over many hours.

Although the novelty does not lie in the basic optical layout,[1] it is important to stress that the counterpropagating instrument we use enables a level of precision that is, in practice, not attainable with widely used commercial systems or with magnetic/acoustic tweezers. Counterpropagating traps allow tether constructs of only a few tens of nanometres long with rigid linkers, without interference between the deflected beams. This regime has been essential for previous single-molecule thermodynamic measurements (e.g., DNA base pairing, ssDNA stacking energies and temperature-dependent determinations of entropy, enthalpy and heat capacity, performed in the laboratory of Prof. Felix Ritort, a co-author of this work). Also, the instrument has been used to test DNA molecules less than 100 bp long subjected to overstretching to demonstrate that S-DNA is indeed a different state than denatured DNA (see Bosaeus et al., 2012. Proceedings of the National Academy of Sciences, 109(38), pp.15179-15184 done in the laboratory of Prof. Bustamante, also a co-author of this work). None of these experiments could have been done with a Lumicks/Impetux-type instrument or with magnetic tweezers because of limitations in force accuracy, bandwidth and short length of the tethers.

Although our intention is not to position SmartTrap as an alternative to the Lumicks instrument, we address this comparison because Reviewer #1 raises it explicitly. Publicly available information about the C-Trap and its "bluelake" software suggests that their "automation" features differ fundamentally from what we demonstrate, both in scope and in robustness. *We also remark that a Lumicks C-Trap is available in the laboratory of Prof. Carlos Bustamante, which means that we have some direct experience with it.*

Regarding the Lumicks system mentioned by Reviewer #1, we have studied in depth the information that the company made public. As we already knew, this system is not comparable to a fully autonomous platform. Indeed, its autonomous operation is shown only for 30 minutes (see screenshot from Lumicks website, on Fig. 1), whereas ours operated for 10 hours. This makes us suspect that their automation is more of a linear set of instructions than a fully autonomous protocol, and that it cannot handle many (if any) problems that could arise during operation. The code that Lumicks published, which shows the automation, is very simple. In some cases, simplicity could be beneficial; however, it does not seem to handle errors. For instance, their "DNA fishing", which is meant to attach DNA molecules, does not even consider the possibility that a particle may not have DNA attached. This means that a single particle without DNA could leave their autonomous system stuck in an infinite loop. We suspect that there are many other issues not addressed by their implementation, e.g., double trapping of particles, multiple DNA molecules attaching, and inconsistent stretching distances (this

[1] We also remark that we have chosen to build our electronics and software for this specific hardware developed in the laboratory of Prof. Carlos Bustamante (a co-author of this article) more than 20 years ago mainly by Dr. Steven Smith (a co-author as well), because this hardware has been used for over 20 years by tens of laboratories around the world (including the laboratories of co-authors Prof Bustamante, Prof. Felix Ritort and Prof. Marcus Wilhemson). This is comparable with the number of Lumicks or Impetux instruments sold to date.

suspicion is reinforced by the quick analysis of the force–extension curves they provide on their website, see below). This may explain why their system only runs for 30 minutes and why they only show a single kind of autonomous experiment.

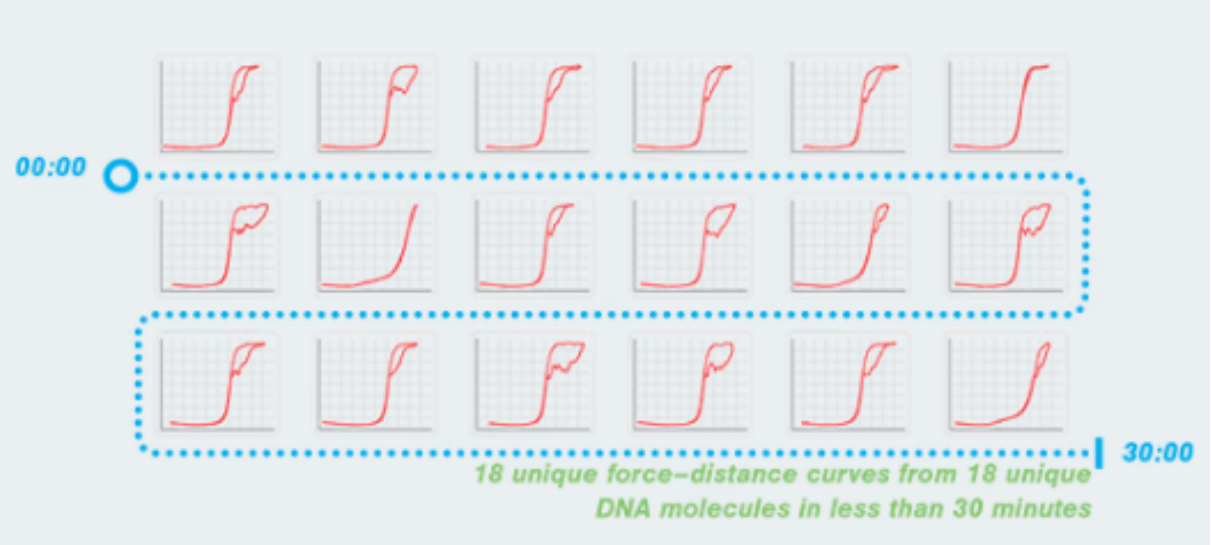

Figure 1: Sceenshot from Lumicks website: https://www.lumicks.com/dynamic-single-molecule/solution/c-trap

They offer no more than a set of curves to support their claim and the quality of this data is questionable. There is a lack of comparison to a theoretical model. Large and obvious differences between the curves at different stages of the protocol are not explained. Many of the curves seem to show an unphysical increase rather than a decrease in the force for short separations.[2] These factors lead us to question whether they can reasonably claim to have achieved autonomous precision measurements.

Furthermore, the "bluelake" control software mentioned is not transferable to other systems. So, even if it were possible to implement certain autonomous protocols using "bluelake", it would be of little to no use to anyone other than Lumicks' own customers (a desirable customer lock-in effect for a startup, but not ideal for the democratization and diffusion of open-science instruments and practices).

In addition, SmartTrap addresses a very practical but fundamental limitation of single-molecule experiments: only a small fraction of bead–molecule constructs are usable, and many must be rejected because they are empty, bear multiple tethers, contain damaged molecules, have weak attachments, or involve extra beads accidentally trapped. Traditionally, an expert operator spends many hours manually sampling and discarding such "bad" molecules in order to find a few good ones, which ultimately limits statistics and throughput. SmartTrap can explicitly automate these screening and validation steps, allowing the instrument itself to carry out the repetitive search for good tethers and to start recording data only when the appropriate conditions are met, while alerting the user if intervention is needed.

Another issue raised in the previous round is that the presented implementation of automatized control is tightly bound to a specific custom-made optics and electronic devices,

[2] On closer consideration, it is likely a case of two or more DNA molecules attaching, which do happen also in our system. However, the increase in force they show at low distances is very strange. It is possibly due to them doing the experiment in an external flow, but we would then expect it to be more consistent among different iterations of the experiment. In any case, we believe that a discussion on the intrinsic quality of the Lumicks C-trap is beyond the scope of our work.

which requires large efforts to replicate. Hence I expect that only a few groups are interested in this innovation and are able to replicate it. The presented setup competes with commercial systems, which are likely more expensive, but actually offer a lower barrier for researchers not experienced with setting up an optical tweezers setup from scratch. In my opinion the broad interest is not given. In this aspect the authors have only given rather unspecific arguments. It seems that if the SmartTrap control software is used without the hardware it belongs to, only few parts can be reused; the object detection and position tracking being the largest part that is independent of the hardware, but also would some effort (retraining) to adapt it to different objects or imaging conditions. On the other side, the custom-made hardware, in particular the optical setup, has rather limited flexibility and it would be difficult to implement signicant modifications. For example, when changing it to a dual beam setup, I see no obvious way to integrate the modified optics within the existing setup without replacing most parts. Any claims related to a broad applicability needs to be more specific. Not everything that can be done is reasonable to do, and specific contribution of this work to possible future developments need to be clearly stated.

We disagree that only a few parts of the SmartTrap control software can be reused. Indeed, in our lab, we already use it with a single-beam standard optical tweezers to perform other kinds of experiments as well as with a holographic optical tweezers setup. The adaptation of the software to these setups took only a few days of work.

Moreover, we would like to emphasize that our platform is not in direct competition with commercial systems. In fact, our platform is a much more robust, flexible, and automated platform than any other system on the market (even without considering its substantially lower cost for the final users).

As Reviewer #1 points out, assuming one has the required funding (>$500,000 for the commercial system Lumicks C-trap system previously mentioned by the reviewer), it is easier to start conducting experiments with commercial systems. Nonetheless, this amount of funding is not always accessible to all research groups. By comparison, components for a custom system based on the SmartTrap cost only about $80,000 and can be implemented following the instructions we provide in any laboratory (again, we remark that the hardware of the SmartTrap has been in use in tens of labs around the World for more than 20 years, giving confidence in its broad usability). Very importantly, this allows for much greater customization, which is necessary for certain applications. One important motivation for our design is precisely to overcome limitations we have encountered with existing commercial systems. For instance, commercial traps are generally optimized for long tethers and are not well-suited to the very short, rigid constructs required for the highest-precision thermodynamic measurements, whereas light-momentum systems such as Impetux, while powerful, are not yet configured for this type of single-molecule manipulation. A counterpropagating instrument combined with SmartTrap's autonomous control therefore fills a gap that is not currently covered by commercial solutions.

We see the most interest in our software from other users in the automation and control of such custom systems. Compared to developing one's own control software from scratch, which is what users with custom systems otherwise must do, adapting the interfaces we provide is very simple. It gives users a single piece of automation-ready software to control their entire system immediately. It provides not only implementations of image analysis, but code for displaying the camera feed, saving it as videos, plotting and saving various signals in real-time, moving the sample stage, controlling microfluidics, and measuring distances, among other valuable

functions. Add to this the autonomous protocols and it becomes apparent that our software fills an important gap.

Finally, even if the image analysis benefits from being tuned to the system in which it is used, our networks are quite robust, especially for detection. As we demonstrate in our tutorial notebook, they can detect different particles in different systems without retraining, see: https://github.com/softmatterlab/SmartTrap/blob/main/Software/Examples/test_SmartTrap_tracker.ipynb

I disagree with some arguments that have been newly added to the manuscript: unlike stated (Section ‚The optical tweezers system'), counterpropagating optical tweezers are not a necessity for light-momentum force detection, nor underfilling of the objective lens aperture. Counter examples are e.g. systems from Impetux, where the only key requirement is high-NA detection of the transmitted light.

It is true that a counterpropagating configuration is not required for direct force measurements (and we do not claim that this is the case). Rather, we claim that a counterpropagating arrangement allows for direct force measurements and makes it easier to work far from surfaces. As stated in the section "The optical tweezers system", under the "Direct force measurement" subsection:

"The primary reason for using counterpropagating beams is that it allows for direct force measurements. […] As an additional benefit, using counterpropagating traps allows for lower NA on the objectives and longer working distances making it easier to work in the bulk of the solution and thereby avoiding surface effects during measurements."

Indeed, working close to a surface makes the Stokes' drag measurements less reliable since the proximity of the beads or trapped objects to the surface increases undesirably their hydrodynamic coupling. Direct force measurement requires high NA in the input and output which limits the space available for the sample. Systems based on light-momentum detection in a co-propagating geometry, such as Impetux, clearly demonstrate that this principle can be implemented in other layouts; however, to our knowledge these instruments are not currently designed for the type of short-tether single-molecule manipulation that our counterpropagating configuration makes routine.

Another topic that in my opinion lacks proper discussion is how the presented approach compares with other methods offering higher throughput. This is an important point because the authors emphasize that their setup is well suited for large-scale studies.

For example for DNA/single molecule studies using magnetic fields (with magnetic beads) or acoustic fields (‚acoustic force spectroscopy') are alternative methods, that allow parallized experiments for thousands of samples simultaneously, thus offering a much higher throughput than a fully automatized optical tweezers setup ever can reach. Accuracy of these alternative methods is limited by the thermal motion of the particles, there is no fundamental benefit when using optical tweezers. Furthermore, for situations where data quality is limited by individual variations of the samples, often a main limitation in life-sciences, or strong fluctuations due to slow, random processes, e.g. for protein binding studies, accuracy of a single measurement is not of importance, but large sample size is needed to obtain reliable data. The general claim that measurement accuracies of optical tweezers exceed alternative

high-throughput approaches is poorly supported and needs more in-depth discussion, explicitly stating which alternative methods exist.

As mentioned in our previous response, there are indeed high-throughput methods that achieve much higher parallelization in specific experiments, such as DNA pulling with magnetic or acoustic tweezers, and they are very valuable when large ensemble statistics are the primary goal. However, optical tweezers offer complementary strengths: higher spatial and temporal resolution, precise force control and the ability to work with very short, rigid tethers, which in turn increases both the spatial and the temporal resolution of the experiments. This has enabled, for example, detailed thermodynamic studies of nucleic acids at the single-molecule level (including DNA base-pairing, ssDNA stacking and temperature-dependent measurements of entropy, enthalpy and heat capacity), which would not be feasible with magnetic tweezers or current commercial systems because they do not provide the necessary force accuracy in this regime, nor the spatial and temporal resolution. Moreover, the high bandwidth of optical tweezers (up to ~1 MHz) has been crucial in precision measurements of Brownian motion and tests of kinetic theory (e.g. in work by Laszlo, Franosch, Raizen and collaborators), underscoring that this class of instrument occupies a distinct niche that goes beyond single-molecule biology.

A more detailed comparison to other methods is unfeasible since we do not have the instruments required to perform direct comparisons, but also unnecessary in our opinion because our goal is not to claim superiority over all high-throughput methods, but rather to show that SmartTrap makes it practical to perform large-scale optical-tweezers studies in precisely those contexts where the unique precision of this technique is scientifically required.

Presentation still needs careful editing required to make the manuscript amenable to readers who are not acquainted with the details of the experimental setup. As mentionen in previous round, the authors talk several times about „the motors" in the main text without explaining that they are used to translate the microscope stage. This information is still hidden deep in the supplementary information. A reader would need to first read all of the SI to be able to grasp the main manuscript.

The main point of the manuscript is to demonstrate the autonomous procedures. Although the details of the instrument are interesting for someone who wishes to replicate the system, they are not the key novelty. It is standard procedure to have a motorized sample stage in optical tweezers, which is why we do not stress this point explicitly in the main manuscript. Still, we have updated the schematics of the instrument shown in Fig. 1b to clarify that the sample stage is motorized.

In summary, the manuscript and the accompanying released software and technical documents provide very valuable information about years of development, but due to its weak novelty and specialized scope I think that it does not fully meet the requirements for publication in Nature Methods. In any case, the questionable claims need to be tempered and supported with evidence.

Reviewer #2:

Remarks to the Author:

I would like to thank the authors for their thorough rebuttal. Overall, I find that the revisions, clarifications, and substantial expansions to the documentation and software repository address nearly all of my major concerns.

We are glad that the Reviewer find our revision substantial and that we managed to directly address most of the concerns.

The authors have now added a conflict-of-interest statement acknowledging that one co-author commercially manufactures optical traps. This resolves this particular aspect of the issue of disclosure.

My primary concern was that SmartTrap is only applicable to a specific optical tweezers implementation. The authors have significantly expanded the GitHub repository, including device interfaces, configuration files, example notebooks, and designs. While the platform will still require some effort to adapt to different sample types (e.g. different types and sizes of objects being trapped), the new documentation and training guidance reduce the barrier somewhat.

We thank the Reviewer for appreciating that we reduced the barriers to implementing and adapting our platform to different systems and sample types.

Clearly, offering full compatibility with all possible systems is not feasible, but we do want to provide a platform that is as easily adaptable as possible to ensure adoption. For this reason, we outline the steps required to adapt the software. We see this software primarily used in other custom built setups rather than commercial ones, so the effort and advantages gained should be compared to that of users developing a software from scratch. Compared to this, the effort of adapting the SmartTrap is minimal and the gains are substantial, not least because the building blocks for autonomous procedures are already implemented. As we and other users adapt the code to other systems, more implementations of things like compatible drivers for third party motors and cameras will become available, which will further lower the barriers to adoption.

Indeed, in our lab, we already use it with a single-beam standard optical tweezers to perform other kind of experiments, as well as with a holographic optical tweezers setup. The adaptation of the software to these setups took only a few days of work.

The authors have made additions to the text which adequately address my concerns regarding performance benchmarking with specific samples. Although the authors cannot provide cross-user benchmarks during this revision cycle, they clarified that SmartTrap performs comparably to experienced users.

Regarding the details I requested for making the setup accessible to the scientific community, the authors now include hardware schematics, bills of materials and extensive software documentation in the GitHub repository. This is an important improvement.

Typographical corrections and definitional clarifications have been made as requested.

We appreciate that the Reviewer found our response on these points satisfying.

Reviewer #3:

Remarks to the Author:

I am satisfied with the Authors' reply to my and other reviewers' comments, and would recommend publication.

We appreciate that the Reviewer found our response satisfying.
